# Taxonomic revision of *Hopliancistrus* Isbrücker & Nijssen, 1989 (Siluriformes, Loricariidae) with redescription of *Hopliancistrus tricornis* and description of four new species

**DOI:** 10.1371/journal.pone.0244894

**Published:** 2021-01-20

**Authors:** Renildo Ribeiro de Oliveira, Jansen Zuanon, Lucia H. Rapp Py-Daniel, José L. O. Birindelli, Leandro M. Sousa

**Affiliations:** 1 Instituto Nacional de Pesquisas da Amazônia (INPA), Coordenação de Biodiversidade (COBIO), Petrópolis, Manaus, AM, Brazil; 2 Departamento de Biologia Animal e Vegetal, Universidade Estadual de Londrina, Londrina, PR, Brazil; 3 Laboratório de Ictiologia de Altamira, Universidade Federal do Pará, Altamira, PA, Brazil; Fundacion Miguel Lillo, ARGENTINA

## Abstract

*Hopliancistrus* is an Ancistrini genus diagnosed by having few and very strong cheek odontodes on interopercular area, and a patch of strong and stiff odontodes on the antero-lateral border of the snout. The type species is herein redescribed based on types and recently collected specimens. In addition, four new congeneric species are described based on specimens collected in other parts of the Rio Xingu and Rio Tapajós basins. *Hopliancistrus tricornis* is distributed in the lower Rio Tapajós and is diagnosed by the possession of four branched anal-fin rays and relatively large white to yellow spots on trunk and pectoral and pelvic fins, and dark brown spots on dorsal, caudal and anal fins. *Hopliancistrus munduruku* is described based on specimens from Rio Jamanxim (Rio Tapajós basin) and Rio Curuá (Rio Xingu basin) and is diagnosed by the possession of five branched anal-fin rays and large yellow blotches on trunk and dark brown to black spots over the fins. *Hopliancistrus wolverine* is distributed in the rapids of the lower and middle Rio Xingu and is diagnosed by the possession of five branched anal-fin rays and conspicuous small yellow dots on head, trunk and fins. *Hopliancistrus xikrin* is distributed in medium- to small-sized tributaries of the lower portion of Rio Xingu basin, and is diagnosed by absence of contact between the transverse process of the first dorsal-fin pterygiophore and the transverse process of the second pterygiophore. *Hopliancistrus xavante* is distributed in the tributaries of upper Rio Xingu basin, and is diagnosed by having a thick skin covering the nuchal plate; by having large white spots on trunk and fins; and by the possession of five branched anal-fin rays. An osteological description and a key for species identification are also provided.

## Introduction

Loricariidae is the fifth largest family of vertebrates in the world and the second largest in the Neotropics [1,2], with more than 1,000 valid species, of which approximately 22% were described only in the last ten years [3]. Among loricariids, Ancistrini is the most diverse tribe of Hypostominae, with approximately 260 valid species. These numbers indicate that freshwater fish diversity is still underestimated, especially in highly species-rich areas such as the Brazilian Amazon.

*Hopliancistrus tricornis* was described as a new species and new genus [4] belonging to a new tribe (Hopliancistrini) within the former Ancistrinae (sensu [4]). Despite being well-known in the aquarium trade (code L212; [5]), the only known preserved specimens of *Hopliancistrus* available in collections at the time of its original description were from the lower Rio Tapajós (collected by the Expedição Permanente da Amazônia in 1970), and from Cachoeira von Martius in upper Rio Xingu basin (collected by Jean-Pierre Gosse in 1964).

During recent expeditions in the main channel and tributaries of the Rio Xingu and Rio Tapajós, several specimens of *Hopliancistrus* were collected and are now available in distinct fish collections. The aim of this paper is to review the diversity and geographic distribution of *Hopliancistrus*, which resulted in the description of four new species. In addition, new diagnoses, a key to species identification and osteological descriptions are provided.

## Material & methods

Most specimens analysed in this study were obtained from museum collections. Recent collected fish were immediately anesthetized using water containing a lethal dose of eugenol (clove oil), and then fixed in 10% formalin, following the guidelines of the Brazilian Society of Ichthyology Lucena et al. [6]. No experimentation was conducted on live specimens. All material was collected in accordance with Brazilian law, under scientific collection licence (SISBIO 31089–2). This study is part of the project number 033/2012 approved by Brazilian ethics committee Comissão de Ética no Uso de Animais (CEUA) of Instituto Nacional de Pesquisas da Amazônia (INPA). Morphological measurements were taken as point-to-point linear distances with a digital caliper to the nearest 0.1 mm following Boeseman [7] and Fisch-Muller et al. [8]. Standard length (SL) is expressed in millimeters and all other measurements are expressed as percents of standard length or head length (HL). Measurements were recorded exclusively in specimens above 60.0 mm SL. Specimens were preserved in alcohol (alc); prepared as cleared and counterstained (cs) for bone and cartilage according to methods of Taylor & van Dyke [9]. Additional osteological observations were made from skeletons prepared from formalin-fixed specimens according to the methods of Bemis et al. [10] and is quoted as “skel” in the list of examined material. Vertebral counts include Weberian Apparatus (five) and ural complexes (one), following Lundberg & Baskin [11]. The nomenclature of the dermal plates on the lateral series follows Schaefer [12]. Osteological nomenclature follows Schaefer [13]. In the color pattern description, we standardized the use of the term “dot” for minute and well-defined round marks, smaller than pupil diameter; and “spot” for marks that are larger than pupil diameter, sometimes irregular in shape, and with blurred contour. Some specimens were excluded from the type series and listed as non-types, for their poor state of preservation, or due to an excessive number of specimens. Museum abbreviations include: ANSP, Academy of Natural Sciences of Philadelphia, Philadelphia; IFPB, Instituto Federal da Paraíba, João Pessoa; INPA, Instituto Nacional de Pesquisas da Amazônia, Manaus; IRSNB, Institut Royal des Sciences Naturelles de Belgique, Bruxelles; LIA, Laboratório de Ictiologia de Altamira, Altamira; MCP, Museu de Ciências e Tecnologia da PUCRS, Porto Alegre; MNRJ, Museu Nacional, Rio de Janeiro; MPEG, Museu Paraense Emílio Goeldi, Belém; MZUEL, Museu de Zoologia da Universidade Estadual de Londrina, Londrina; MZUSP, Museu de Zoologia da Universidade de São Paulo, São Paulo; NUP, Núcleo de Pesquisas em Limnologia, Ictiologia e Aquicultura da Universidade Estadual de Maringá, Maringá; UFPA, Universidade Federal do Pará, Belém; UFRO-I, Universidade Federal de Rondônia, Porto Velho; ZMA, Zoölogisch Museum, Universiteit van Amsterdam, Amsterdam; ZUEC, Museu de Zoologia da Universidade Estadual de Campinas "Adão José Cardoso", Campinas.

### Nomenclatural acts

The electronic edition of this article conforms to the requirements of the amended International Code of Zoological Nomenclature, and hence the new names contained herein are available under that Code from the electronic edition of this article. This published work and the nomenclatural acts it contains have been registered in ZooBank, the online registration system for the ICZN. The ZooBank LSIDs (Life Science Identifiers) can be resolved and the associated information viewed through any standard web browser by appending the LSID to the prefix “http://zoobank.org/”. The LSID for this publication is: urn:lsid:zoobank.org:pub:CB15E2DF-A52F-4569-B52F-41E3C181E33A. The electronic edition of this work was published in a journal with an ISSN, and has been archived and is available from the following digital repositories: PubMed Central, LOCKSS.

## Results

### *Hopliancistrus* Isbrücker & Nijssen, 1989

*Hopliancistrus* Isbrücker & Nijssen [4]: 543 (Type species: *Hopliancistrus tricornis* Isbrücker & Nijssen [4], type by original designation, gender: masculine).

#### Diagnosis

**Fig 1:**
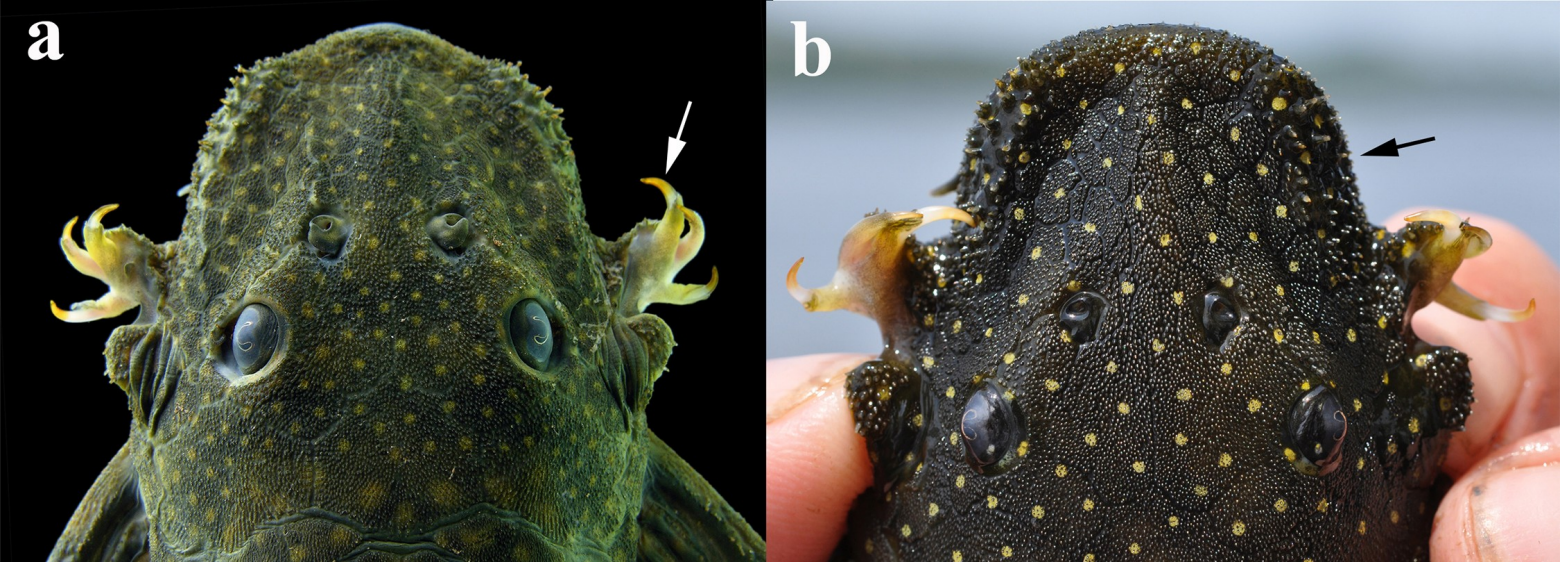
Dorsal view of head of *Hopliancistrus munduruku* and *H*. *wolverine*. Showing the synapomorphies of the genus: Three hypertrophied odontodes on cheek plates (white arrow) and stiff odontodes on antero-lateral border of snout (black arrow). Photographed by M. Sabaj.

**Fig 2:**
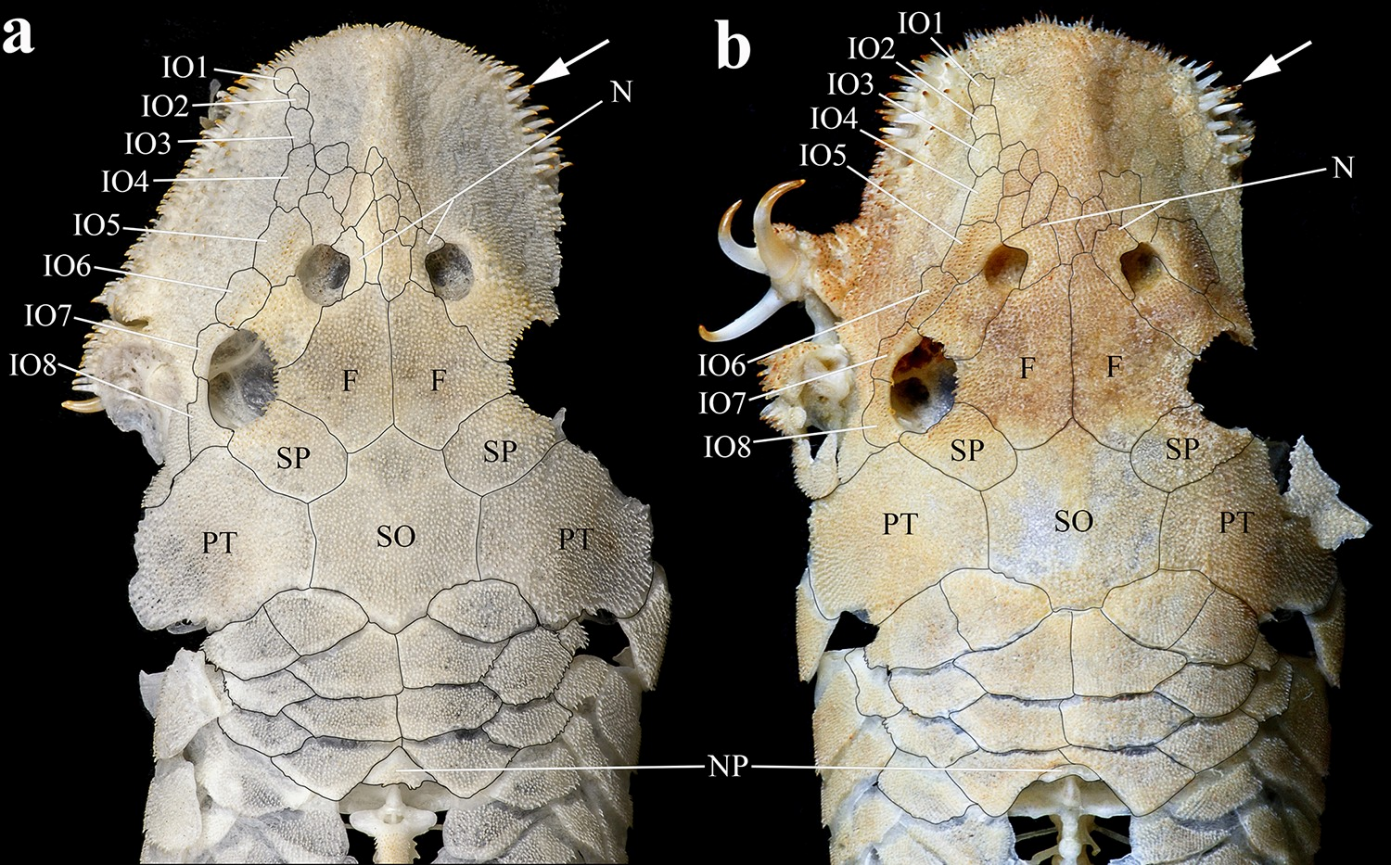
Dorsal view of anterior portion of body. (a) *Hopliancistrus wolverine*, INPA 43731, 122.2 mm SL; (b) *H*. *xavante*, INPA 37616, 114.7 mm SL. Arrows point the patch of strong and stiff odontodes on the antero-lateral border of the snout. F- frontal, IO1-IO8- infraorbitals 1 to 8, N- nasal, NP- nuchal plate, PT- compound pterotic, SO- Parieto-supraoccipital, SP- sphenotic.

*Hopliancistrus* is diagnosed by having two exclusive features among Loricariidae: the presence of only three hypertrophied odontodes on the eversible cheek plates, and a patch of strong and stiff odontodes on the antero-lateral border of the snout (Figs 1 and 2). Other non-exclusive diagnostic features include: spinelet reduced (dorsal-fin locking mechanism not functional); five series of plates on caudal peduncle; head and body depressed (body width two times body depth); abdomen completely naked; premaxilla and dentary of moderate length and disposed parallel to anterior border of snout; teeth bifid, delicate and similar number and size on premaxilla and dentary.

#### Distribution

The species of *Hopliancistrus* are apparently restricted to the Rio Xingu and Rio Tapajós basins, the two main drainages of the Brazilian Cratonic Shield that flows North to the Rio Amazon.

#### Popular name

The species of the genus are known in the aquarium trade as tricornis-pleco and “alicate” (= pliers) and “ancistrus-de-unha” (= clawed ancistrus) in Portuguese.

#### Ecological notes

Specimens of all species of the genus were collected exclusively in fast-flowing, well-oxygenated clear waters in stretches with rocky bottom. When disturbed, specimens resolutely evert the three hypertrophied odontodes, clipping the aggressor with their stiff cheek plates odontodes.

### *Hopliancistrus tricornis* Isbrücker & Nijssen, 1989

(Figs 3–5A)

*Hopliancistrus tricornis* Isbrücker & Nijssen [4]: 543, Figs 5–7 [Type locality: Brasilien, Est. Pará, poça de pedra no Rio Tapajós, São Luis; original description]. −Seidel & Evers [14]: 323 [picture of live adult specimen. Same picture was erroneously used in Camargo et al. [15]: 159 to depict *Hopliancistrus* "mancha" (L171) from Xingu], 325 [picture of young specimen]. −Seidel [16]: 107 [Fig 7, L212, Tapajós].
*Hopliancistrus* (L212).–Stawikowski [17]: 480 [DATZ magazine, new loricariids from Tapajós available to the hobby]. −Schraml & Schäfer [18]: 162 [Aquarium catalog].

**Fig 3:**
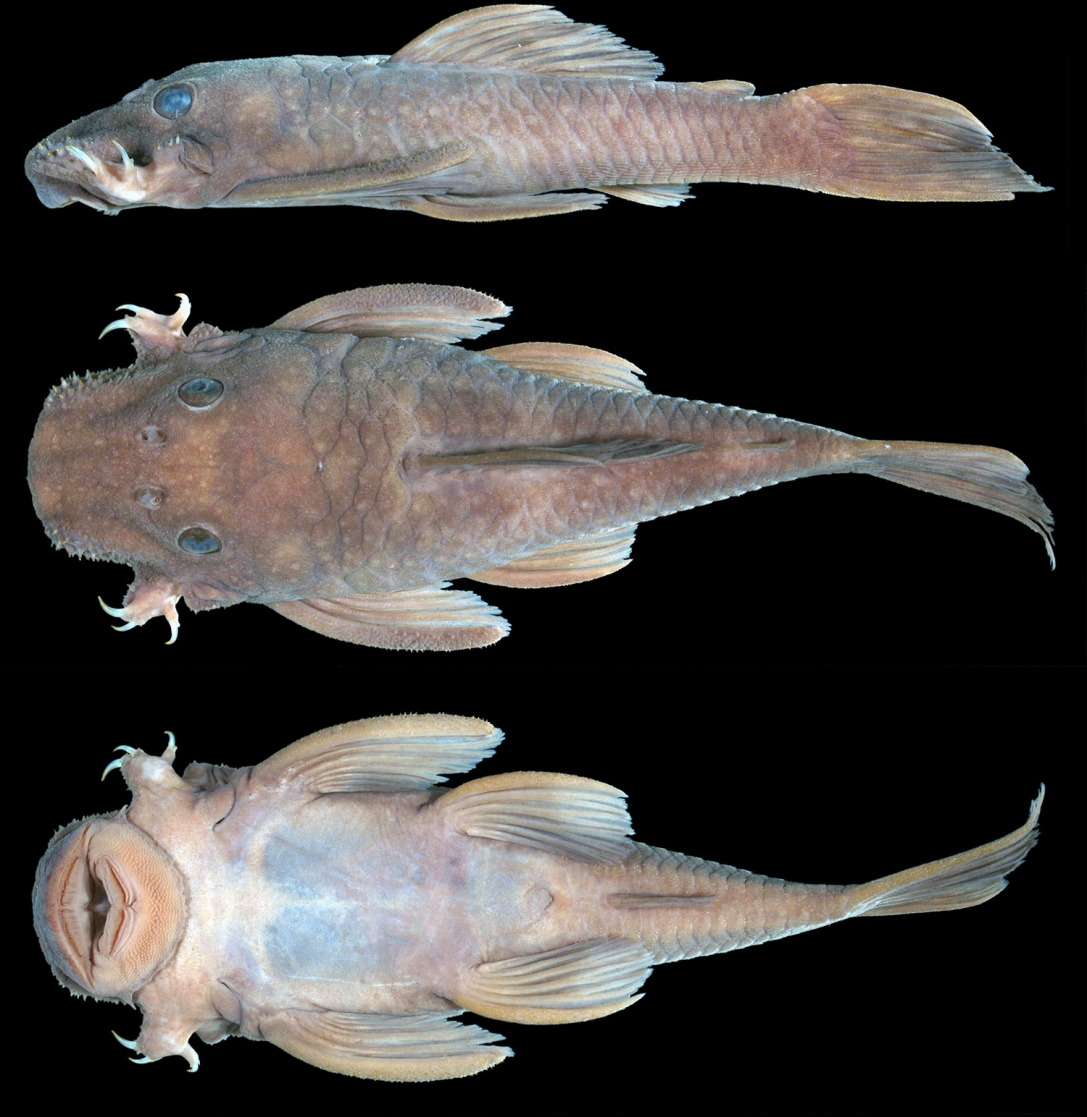
Holotype of *Hopliancistrus tricornis* Isbrücker & Nijssen, 1989. MZUSP 22007, 104.1 mm SL, from Rio Tapajós.

**Fig 4:**
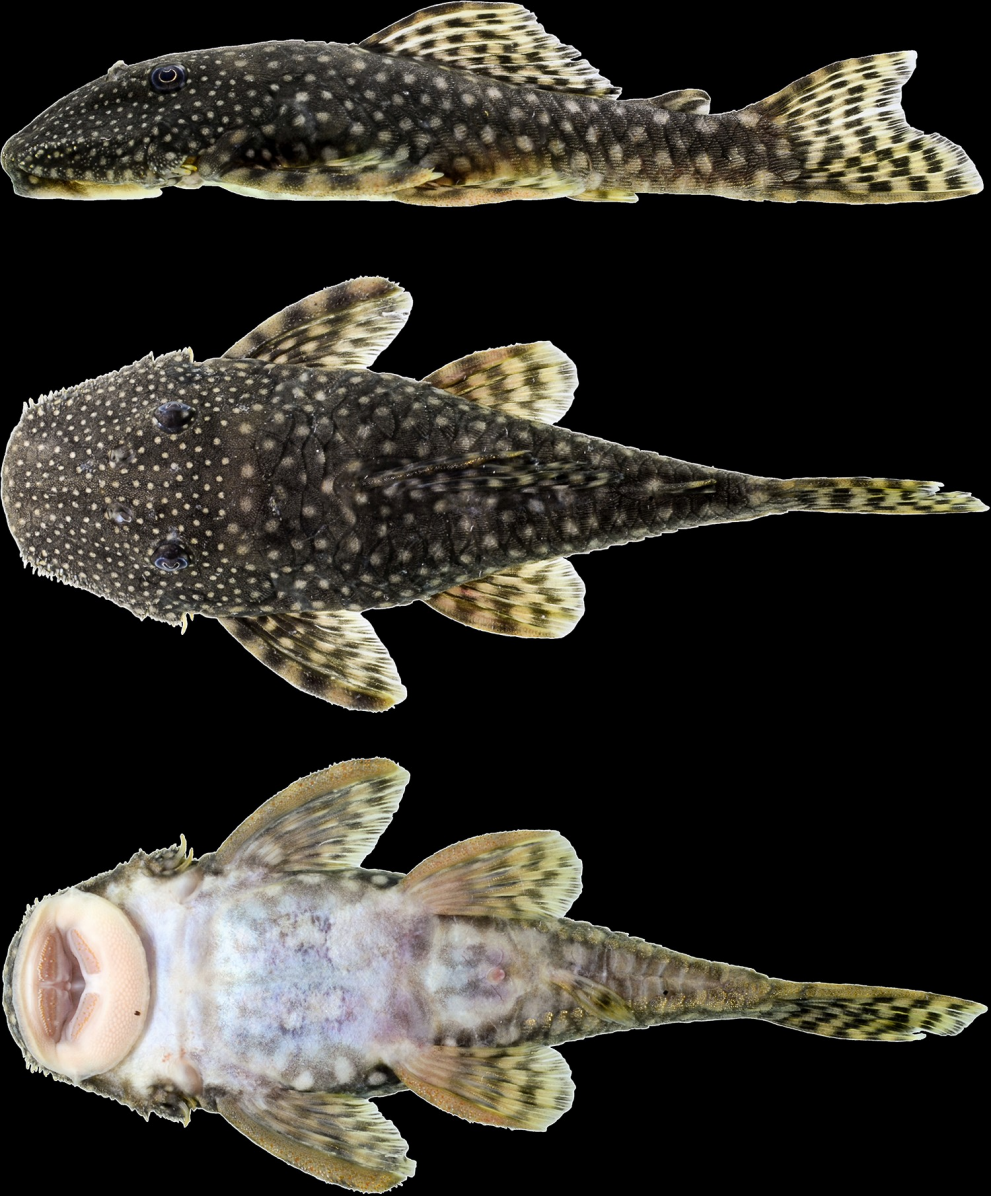
Live specimen of *Hopliancistrus tricornis*. INPA 58207, 96.5 mm SL, from Leitoso stream, a tributary of Rio Cupari, Rio Tapajós basin.

**Fig 5:**
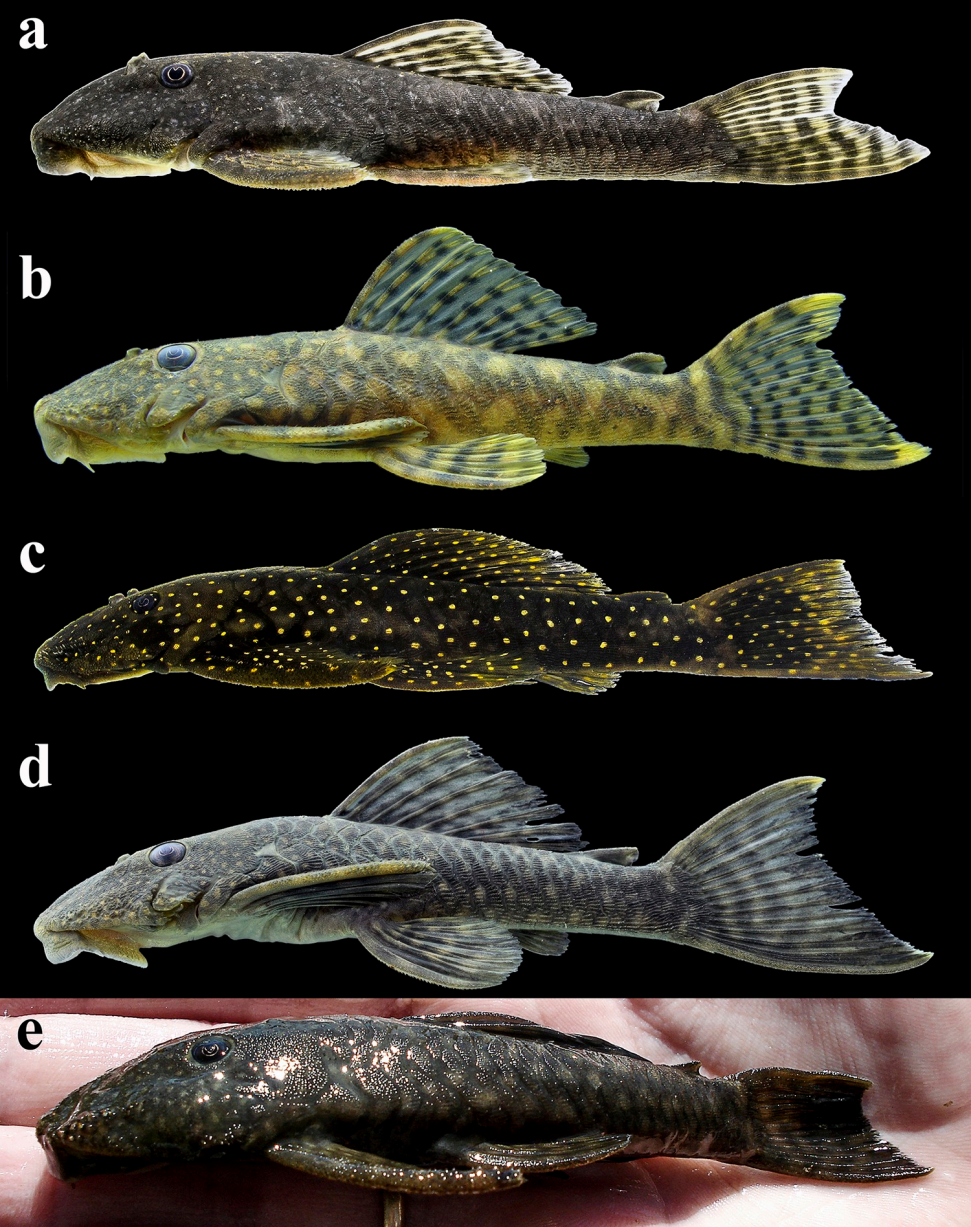
Lateral views of alive specimens. (a) *Hopliancistrus tricornis* INPA 58207, 77.4 mm SL, from Leitoso stream, Rio Tapajós basin; (b) *H*. *munduruku*, MZUSP 96964, 77.8 mm SL, paratype from Rio Curuá; (c) *H*. *wolverine* INPA 52276, 125.0 mm SL, paratype from Rio Xingu; (d) *Hopliancistrus xikrin*, ANSP 193087, 70.0 mm SL, paratype from Rio Bacajá and (e) *H*. *xavante* (uncatalogued specimen), from Rio Culuene. Photographed by M. Sabaj-Pérez (b, c and e).

#### Diagnosis

**Fig 6:**
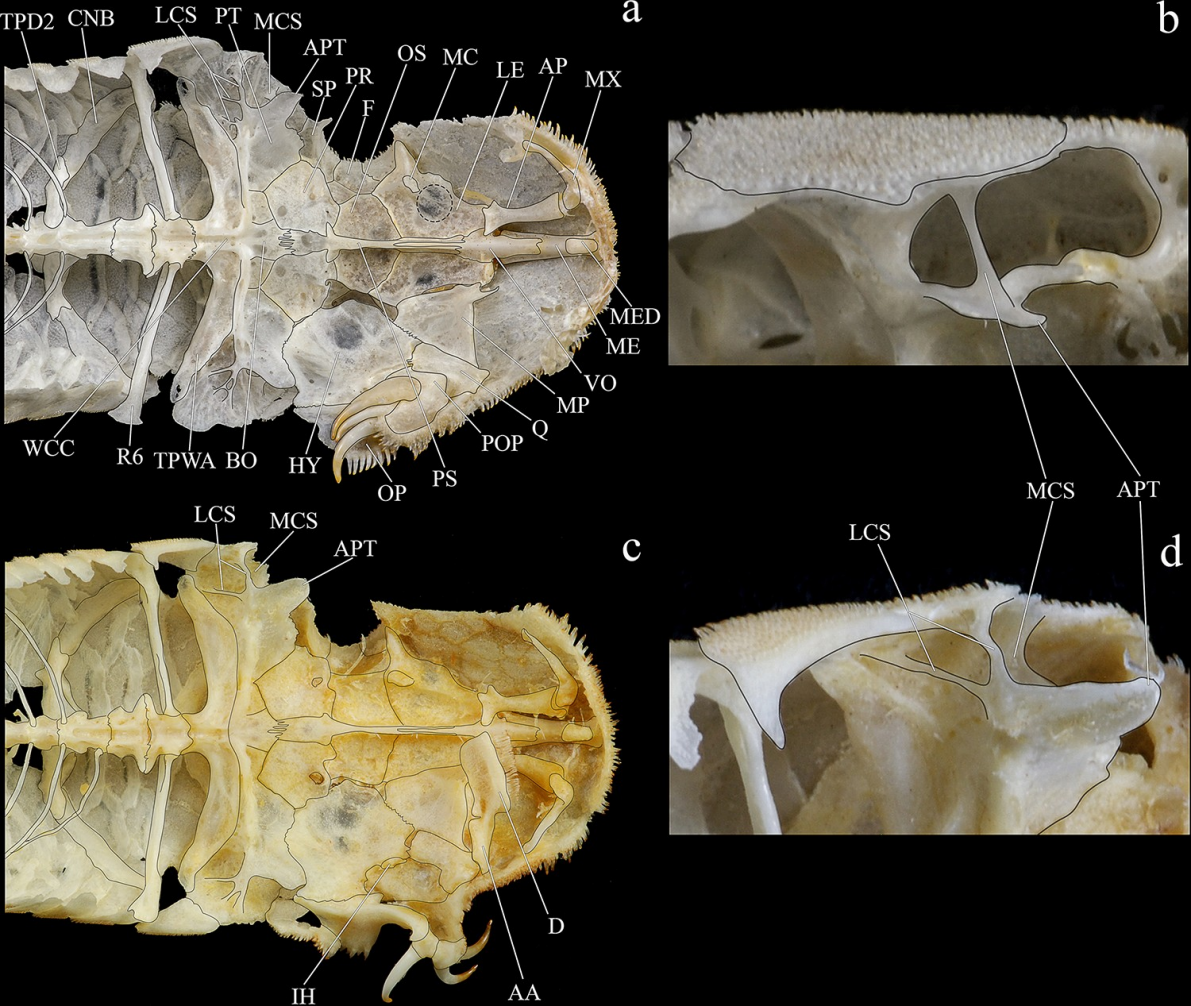
Ventral and latero-ventral (enlarged) views, right side, of anterior portion of body. (a, b) *Hopliancistrus wolverine*, INPA 43731, 122.2 mm SL; (c, d) *H*. *xavante*, INPA 37616, 114.7 mm SL. AA- angulo articular, AP- autopalatine, APT- anterior process of compound pterotic, BO- basioccipital, CNB- connecting bone, D- dentary, F- frontal, HY- hyomandibula, IH- interhyal, LCS- latero-posterior connection strut, LE- lateral ethmoid, MC- metapterygoid condyle of lateral ethmoid, MCS- mesial connection strut, ME- mesethmoid, MED- mesethmoid disk, MP- metapterygoid, MX- maxilla, OP- opercle, OS- orbitosphenoid, POP- preopercle, PR- prootic, PS- parasphenoid, PT- compound pterotic, Q- quadrate, R6- expanded rib of sixth vertebra, SP- sphenotic, TPD2- transverse process of second dorsal-fin pterygiophore, TPWA- transverse process of the Weberian apparatus, VO- vomer, WCC- weberian complex centrum. Dashed circle indicates the extent of the nasal capsule.

**Fig 7:**
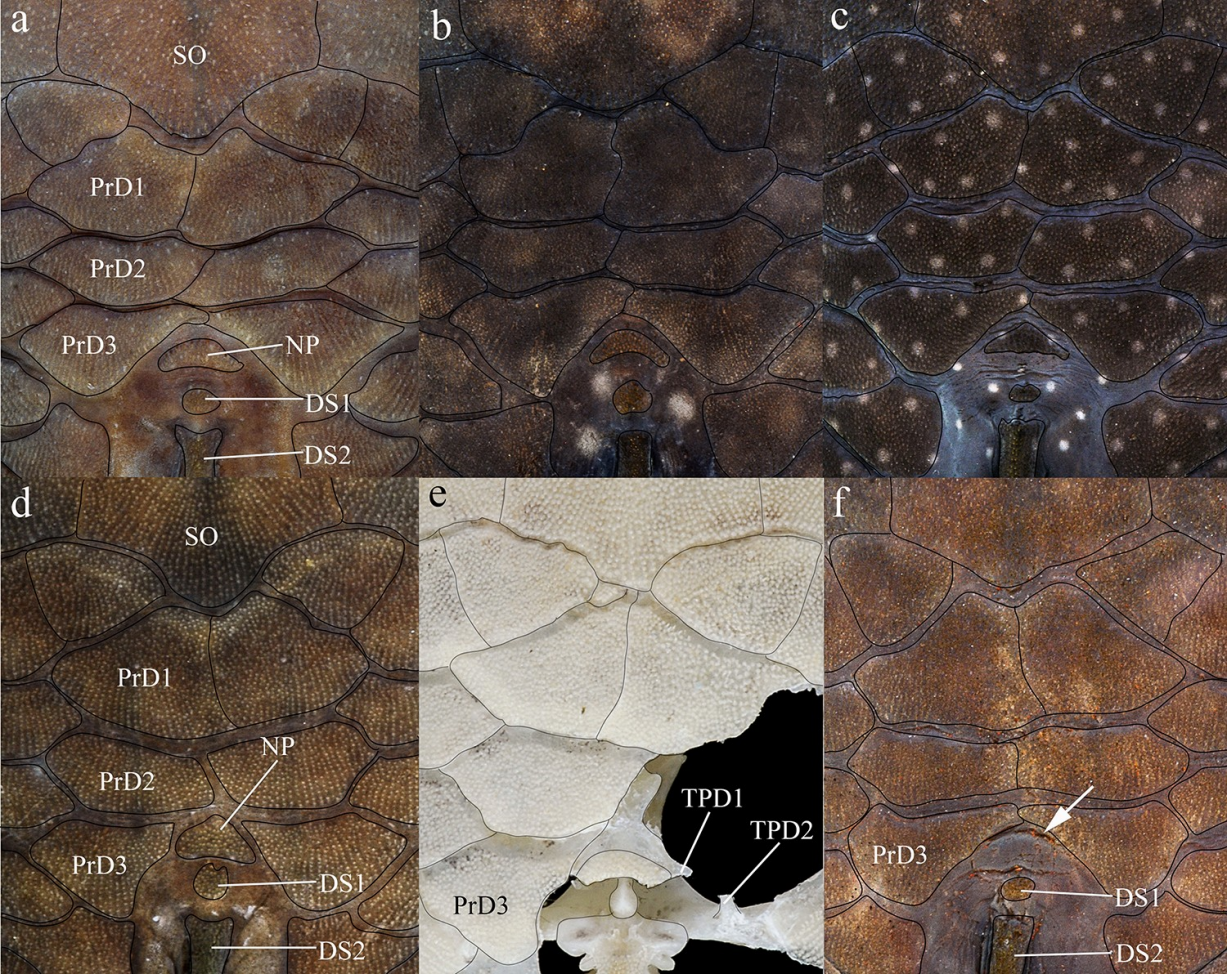
Dorsal view of predorsal region. (a) *Hopliancistrus tricornis*, INPA 6775, 124.7 mm SL, (b) *H*. *munduruku*, MZUSP 119674, 124.6 mm SL, (c) *H*. *wolverine*, INPA 40875, 136.3 mm SL; (d, e) *H*. *xikrin*, INPA 40905, 99.4 mm SL and INPA 40904, skel, 133.8 mm SL; (f) *H*. *xavante*, INPA 37616, 106.0 mm SL. Arrows point the nuchal plate covered by skin. DS1- first dorsal-fin spine or spinelet, DS2- second dorsal-fin spine, NP- nuchal plate, PrD1-PrD3- first to third predorsal plate pair, SO- Parieto-supraoccipital.

*Hopliancistrus tricornis* is distinguished from its congeners by having four branched rays on anal fin (vs. five branched rays); *Hopliancistrus tricornis* is distinguished from its congeners except *H*. *munduruku* by having large yellowish-white spots along the body, and dark brown spots on fins (vs. body covered by conspicuous small greenish-yellow dots of similar size on head, trunk and fins in *H*. *wolverine*; yellowish-white spots on posterior portion of the body moderate in size, usually smaller than pupil in *H*. *xikrin*; all fins covered by large yellowish-white spots in *H*. *xavante*). *Hopliancistrus tricornis* can be distinguished from its congeners except *H*. *wolverine* by a narrow, bar-shaped connection strut between anterior process of compound pterotic and main body, leaving a large posterior gap (vs. connection strut as a continuous sheet, see Fig 6). *Hopliancistrus tricornis* differs from *H*. *wolverine* and *H*. *xikrin* by pectoral-fin spine length 25.1–29.9% of SL (vs. 32.1–38.4% of SL and 32.1–35.7% of SL, respectively). It also differs from *H*. *xikrin* by the transverse process of first dorsal-fin pterygiophore sutured to the transverse process of the second pterygiophore (vs. absence of contact between the transverse processes of first and second dorsal-fin pterygiophores). *Hopliancistrus tricornis* differs from *H*. *xavante* by caudal peduncle depth 9.7–11.3% of SL (vs. 11.5–12.9% of SL); by narrow nasal bone plate (vs. broad nasal, sometimes slightly triangular, see Fig 2); and by having nuchal plate exposed, and covered by odontodes (vs. nuchal plate covered by thick skin and usually lacking odontodes, see Fig 7).

#### Description

Body shape and pigmentation in Figs 2–5A. Morphometric data and counts in Table 1. Medium-sized loricariid, with largest examined specimen reaching 132.1 mm SL. In dorsal view, head roughly quadrangular with snout outline; body gradually narrowing from cleithrum to caudal-fin origin. Greatest body width at cleithrum. Head and trunk moderately depressed, greatest depth at dorsal-fin origin. In lateral view, dorsal profile straight or slightly convex from snout tip to supraoccipital process; straight from tip of supraoccipital process to dorsal-fin origin, then straight or gently declining to first procurrent caudal-fin ray. Ventral profile straight from snout tip to caudal-fin origin.

**Table 1:** Morphometric data and counts of *Hopliancistrus tricornis* from lower Rio Tapajós basin and *H*. *munduruku* from upper Rio Jamanxim, Rio Tapajós basin and Rio Curuá, Rio Xingu basin.

	*Hopliancistrus tricornis*						*Hopliancistrus munduruku*					
Measurement	H	Mean	Min	Max	SD	N	H	Mean	Min	Max	SD	N
Standard length	104.1		61.1	124.7		46	155.3		55.5	155.3		58
**% of standard length**												
Predorsal length	45.5	45.4	42.2	48.7	1.1	46	44.5	46.0	44.0	48.7	1.1	58
Head length	36.5	36.9	34.8	40.6	0.9	46	36.5	37.5	35.6	39.0	0.9	58
Cleithral width	33.9	34.6	32.9	37.4	1.0	46	34.7	35.2	33.5	37.1	0.8	58
Cleithral process width	32.0	32.4	30.5	35.2	1.1	46	31.9	32.7	30.9	35.1	0.9	58
Pectoral-Pelvic origin length	23.3	24.5	22.6	28.3	1.3	46	24.7	24.6	20.4	28.5	1.6	58
Pectoral-spine length	29.9	27.9	25.1	29.9	1.3	46	30.0	28.7	24.5	30.9	1.6	58
Pelvic-anal origin length	24.8	23.5	21.6	26.4	1.1	46	24.2	23.4	21.4	25.1	0.9	58
Pelvic-spine length	24.6	23.4	21.0	25.2	1.1	46	21.4	23.3	20.9	24.8	0.9	58
Postanal length	27.0	27.2	24.6	29.0	1.0	46	26.9	26.3	24.3	28.2	0.9	58
Anal-fin spine length	10.6	9.1	7.0	10.6	0.9	46	9.2	9.5	7.7	12.4	0.8	58
Dorsal spine length	21.8	22.5	21.0	25.4	1.0	46	22.0	22.8	20.9	25.2	1.0	52
Dorsal-fin base length	22.0	24.7	22.0	27.8	1.2	46	24.6	25.9	23.8	27.9	0.9	58
Dorsal-adipose distance	15.8	11.7	8.2	15.8	1.3	46	13.2	11.1	9.1	15.0	1.2	58
Caudal peduncle depth	10.7	10.6	9.7	11.3	0.4	46	11.1	10.7	10.1	11.3	0.3	58
Adipose-spine length	6.5	7.7	6.3	9.0	0.7	46	7.4	7.4	5.6	9.4	0.8	58
Adipose-Caudal length	20.7	22.9	20.7	25.0	0.9	46	21.1	22.0	20.4	24.3	0.9	58
Body depth at dorsal-fin origin	14.7	16.8	14.1	19.1	1.2	46	16.7	17.7	15.2	21.9	1.3	58
Body Width at dorsal-fin origin	30.0	28.7	26.2	32.6	1.5	46	29.1	28.9	26.6	31.9	1.3	58
Body Width at anal-fin origin	16.0	16.2	14.8	19.3	0.9	46	18.3	17.7	15.8	20.0	1.0	58
Postdorsal length	34.7	33.1	23.1	35.4	1.8	46	32.7	31.8	26.1	34.7	1.4	58
Anus-anal fin length	6.7	6.0	4.3	7.4	0.8	46	7.3	6.4	5.0	9.5	0.7	58
**% of Head length**												
Orbital diameter	17.1	17.2	15.2	20.0	1.2	46	13.2	16.6	13.1	19.1	1.4	58
Snout length	58.4	61.3	57.6	65.4	2.1	46	63.8	61.8	57.0	64.8	1.7	58
Internares width	14.4	13.0	10.9	16.3	1.0	46	16.9	14.1	11.3	17.0	1.4	58
Interorbital width	34.3	34.4	31.1	37.2	1.4	46	38.2	34.5	30.6	39.6	1.8	58
Head depth	41.7	43.7	41.2	47.1	1.4	46	43.5	44.2	40.2	48.8	2.0	58
Dentary tooth row length	20.0	21.4	19.1	23.7	1.3	46	18.7	20.9	17.7	24.4	1.6	58
Premaxillary tooth row length	20.0	21.4	18.9	23.5	1.3	46	19.5	21.6	19.0	24.6	1.4	58
Head width	94.6	97.5	88.5	103.3	2.9	46	96.8	96.9	90.5	104.4	3.1	58
Eye-nare length	10.6	12.2	10.2	14.0	1.0	46	12.9	12.3	9.6	14.5	0.9	58
Interbranchial distance	51.1	55.2	48.7	62.8	3.2	46	54.9	55.3	50.0	61.0	2.2	58
Lower lip width	69.0	66.4	58.6	72.1	3.5	37	62.4	64.7	58.8	69.5	2.7	25
Lower lip length	16.3	16.4	13.9	18.7	1.2	37	15.7	16.1	13.7	18.1	1.1	25
**Counts**		**Mode**						**Mode**				
Premaxillary teeth	56	34	26	56		46	61	53	39	64		58
Dentary teeth	59	49	30	59		46	59	59	38	67		58
Total lateral median plates	25	24	23	25		46	24	24	23	25		58
Plates between anal and caudal fins	9	9	8	10		46	9	9	8	10		58
Plates between dorsal and adipose fins	5	4	3	5		46	5	4	3	5		58
Predorsal plates	3	4	2	4		46	3	3	3	4		58

H = holotype, SD = standard deviation, n = number of measured specimens.

Body anterior region roughly trapezoidal, depressed dorsoventrally, and slightly oval at caudal peduncle in cross section. Weak ridge from snout tip to anterior nare, and from posterior nare to eye. Snout completely covered by plates except for small naked area on tip. Lateral border of snout covered by short stiff odontodes. First four plates of mid-ventral series gently keeled. Ventral surface of caudal peduncle flat; small and delicate keel at three or four last ventral plates series of caudal peduncle. Remaining of body plates without keels.

Head large and wide. Eye moderate in size, dorsolaterally positioned at midpoint of head length; iris operculum present. Orbit not elevated; interorbital area nearly straight. Parieto-supraoccipital almost indistinct from other skull bones. Supraoccipital process not elevated, slightly rounded posteriorly. Parieto-supraoccipital limited posteriorly by four roughly triangular to trapezoidal plates. Predorsal area with three pairs of predorsal plates, plus one very small nuchal plate anterior to dorsal-fin spinelet.

Mouth and lips of moderate size; oral disk elliptical; lips almost completely covered with small round papillae densely packed, slightly larger on central portion of lower lip; smooth area surrounding premaxillary and dentary, without papillae. Lower lip large, but not reaching pectoral girdle. Maxillary barbel short and with small portion free from lower lip, except in some individuals with maxillary barbel completely fused into lower lip. Teeth short, thin, delicate, and bicuspid. Mesial cusp slightly larger and wider than lateral cusp; lateral cusp reaching two-thirds of mesial cusp length. Premaxilla and dentary of similar size and disposed parallel to anterior border of snout. Single and reduced buccal papilla between premaxilla present in most specimens.

Dorsal body surface completely covered by large plates, except immediately around dorsal-fin base. Ventral surface entirely devoid of plates from snout to anal-fin origin. Base of first anal-fin pterygiophore covered by skin, preanal plate absent. Caudal peduncle completely covered by plates. Median series plates 23–25. Five series of lateral plates at caudal peduncle. Five to seven oblong plates on caudal-fin base. Opercle exposed, sickle-shaped and covered by odontodes; plates immediately posterior to opercle small, sometimes reaching one-third of length of exposed opercle. Plates of supraopercular area small and few in number, leaving large naked area around opercle. Three large, strong, and curved hook-like odontodes on cheek plates, reaching posterior margin of branchial opening; fleshy and thick odontode sheath, sometimes covering two-thirds of cheek odontodes. All plates covered with small and aligned odontodes, slightly larger on opercle, postopercular plate and pectoral-fin spine.

Dorsal-fin origin on anterior portion of body, slightly anterior to vertical through pelvic-fin origin. Dorsal-fin II,7; spinelet present and very reduced, dorsal-fin locking mechanism not functional. Dorsal fin short and low, not reaching adipose fin when adpressed, nor even reaching preadipose plate in some specimens. Four to five plates separating dorsal from adipose fin. Adipose fin short and low. Usually two unpaired dorsal plates between adipose fin and first procurrent caudal-fin ray. Caudal-fin i,14,i, slightly emarginated, with oblique distal margin, ventral lobe slightly longer. Pectoral fin I,6, large, surpassing pelvic-fin base when adpressed. Pectoral-fin spine thick and strong, not pungent, covered by odontodes. Pelvic fin i,5, reaching end of anal-fin base when adpressed. Anal fin short, i,4, (except in two specimens of MNRJ 35602 and MNRJ 35604 with i,3 rays in anal fin). All rays covered by numerous short odontodes on their free surface. Anteriormost branched rays of pectoral, pelvic and anal fins longer than unbranched rays. Four to five dorsal and ventral procurrent caudal-fin rays. Vertebrae 28 (6). Seven (4) or eight (2) rib pairs.

#### Color in alcohol

Body ground color light brown at dorsum and sides. Head covered by yellowish-white dots, smaller than pupil diameter; dorsum and sides covered by larger yellowish-white blotches, larger than pupil diameter. All fins with dark brown or black marks; dorsal and caudal fins covered by peculiar conspicuous black rings that encircling the lepidotrichia, giving overall aspect of black wings; marks roughly aligned on bands. Pectoral and pelvic fins with light brown membranes, covered by dark spots on dorsal surface also aligned on bands. Ventral surface cream colored (Fig 3).

#### Color in life

Body ground color dark brown at head, dorsum, and sides. Head covered by small yellow dots; dorsum and sides covered by yellow spots larger than pupil. Dorsal, caudal and anal fins yellowish-white, covered by dark brown to black spots over rays. Pectoral and pelvic fins with dark brown membranes, covered by yellowish-white spots on dorsal surface. Ventral surface white, with gray blotches. In specimens from Rio Tapajós and main tributaries as Rio Jamanxim, background color and spots on body and fins less conspicuous than specimens from smaller tributaries, as Leitoso stream (Figs 4 and 5A).

#### Distribution

**Fig 8:**
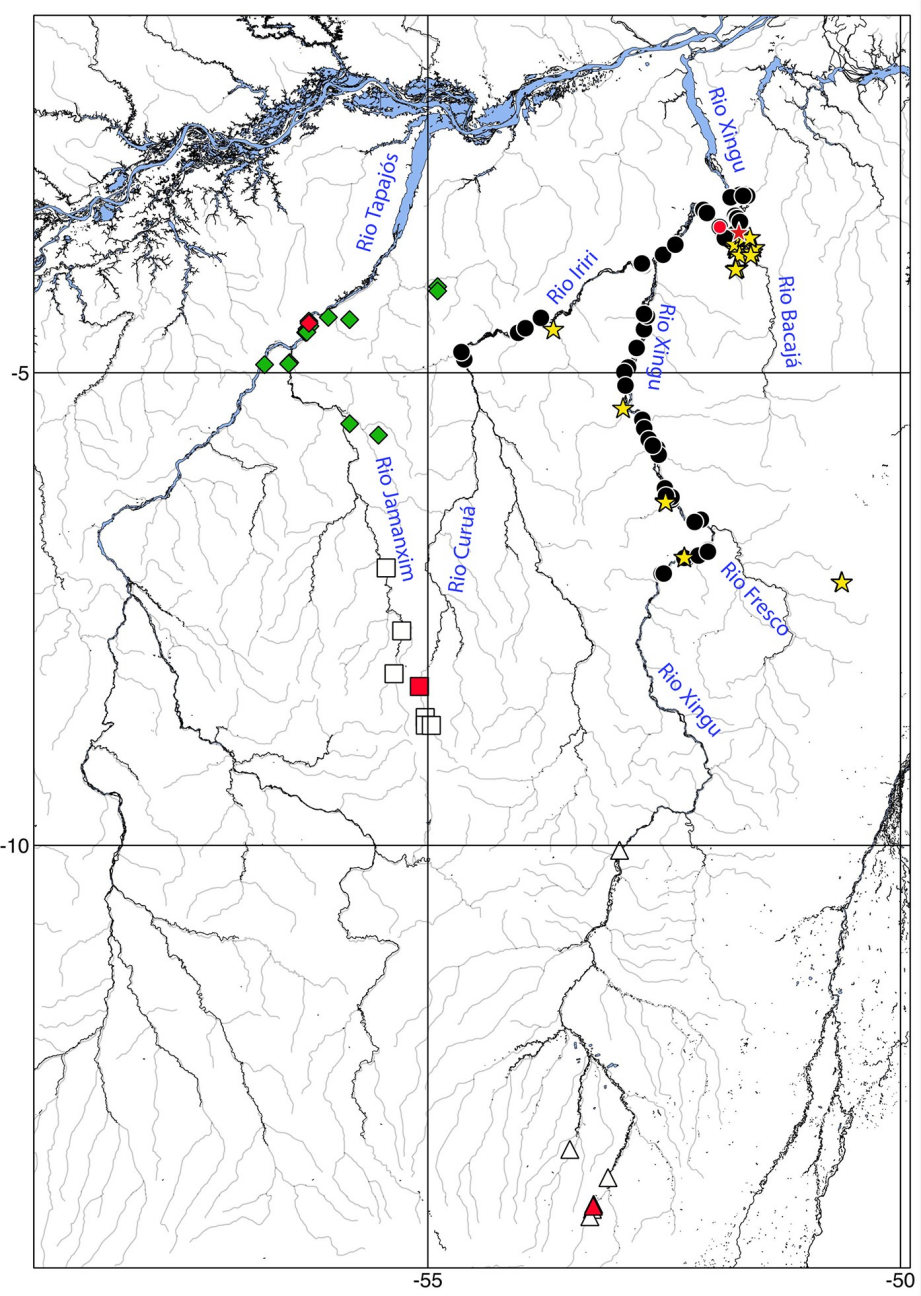
Distribution of *Hopliancistrus* species from Tapajós and Xingu basins. *Hopliancistrus tricornis* (diamonds); *H*. *munduruku* (squares); *H*. *wolverine* (circles); *H*. *xikrin* (stars) and *H*. *xavante* (triangles). Red symbols indicate type localities.

*Hopliancistrus tricornis* is apparently restricted to the middle portion of the Rio Tapajós basin in the São Luiz rapids, and its main tributaries, such as the lower portions of Rio Jamanxim and Rio Itapacurá. This species is also found in small tributaries, such as Igarapé Leitoso, tributary of the Rio Cupari, Rurópolis municipality, Pará State (Fig 8).

#### Remarks

Isbrücker & Nijssen [4] included only the measurements and counts of the holotype in the description of *Hopliancistrus tricornis*, and possibly did not notice the difference in the number of anal-fin rays in the paratypes from the Xingu basin. However, they reported that the specimens from the Xingu basin were distinct from those from the Tapajós basin by having more conspicuous light-colored blotches on head and body. This difference proved to be consistent in our study and useful in recognizing the two species as distinct. One lot of paratypes (MZUSP 24304) of *H*. *tricornis* included a small specimen (24.1 mm SL) of *Ancistrus* sp.

#### Examined material

All from Brazil, Pará, lower Rio Tapajós basin: MZUSP 22007, holotype, 104.1 mm SL, São Luis, Poça de pedra, Rio Tapajós. Paratypes: MZUSP 24304, 6, 35.7–76.8 mm SL, São Luis, Cachoeira do Maranhãozinho, perto de São Luis, Rio Tapajós. MZUSP 26837, 7, 37.5–56.9 mm SL, São Luis, Cachoeira do Lombo de Anta, near São Luis, Rio Tapajós. MZUSP 27664, 6, 57.5–96.0 mm SL, same data as holotype. **Non-types:** INPA 6775, 15 alc, 46.5–124.7 mm SL and 2 cs, 56.8–88.0 mm SL, Rio Jamanxim, Terra Preta Island, 4°53’31.4”S 56°27’43.5”W. INPA 6898, 1, 81.8 mm SL, Rio Jamanxim, Terra Preta Island, 4°54’19.6”S 56°28’21.7”W. INPA 7025, 179, alc, 30.1–132.1 mm SL and 3 skel, 111.1–122.0 mm SL, Rio Tapajós, Pimental, 4°34’14.6”S 56°16’48.7”W, 23 Oct 1991. INPA 35308, 1, 104.1 mm SL, Rio Itapacurá, near bridge, 2°24’48”S 56°03’10”W. INPA 43779, 9, 39.7–86.0 mm SL, Rio Tapajós, near Pimental Village, 4°34’25”S 56°17’33”W. INPA 43780, 5, 42.2–72.0 mm SL, Pimental stream tributary of Rio Tapajós, 4°54’50.2”S 56°43’39.1”W. INPA 45418, 2 alc, 31.3–81.3 mm SL and 1 cs, 38.5 mm SL, stream tributary of Rio Jamanxim, 5°39’35.5”S 55°31’21.2”W. INPA 58207, 26 alc, 43.8–95.7 mm SL and 2 cs, 81.3–93.8 mm SL, Rurópolis, Igarapé Leitoso, tributary of Rio Cupari, urban area of the municipality of Rurópolis, 4°5’20.6”S 54°53’48.3”W. MCP 44384, 17, 29.6–136.7 mm SL, Santarém, Rio Tapajós, between Santarém and Itaituba. MNRJ 35602, 17, 52.9–104.3 mm SL, Itaituba, stream on road BR-163, ca. of 12 km from Transamazônica highway, 4°26’18”S 55°49’41”W. MNRJ 35603, 31, 33.7–88.7 mm SL, Ruropolis, Igarapé Leitoso on Cachoeira bathing area, urban of the municipality of Rurópolis, 4°8’4.84”S 54°53’48.7”W. MNRJ 35604, 29, 21.0–88.2 mm SL, Aruri Grande, Igarapé Gui, ca. 131 km of Trairão on road BR-163, Rio Jamanxim drainage, 5°32’22”S 55°49’38”W. MZUSP 34277, 1, 88.6 mm SL, Rio Tapajós, São Luis Village, upstream Itaituba, 4°12’00”S 55°50’00”W.

### *Hopliancistrus munduruku*, new species

urn:lsid:zoobank.org:act:826A0AF1-965E-4BFC-9966-C9A65252755B

(Figs 1A, 5B and 9)

*Hopliancistrus* sp. (L361). −Werner [19] 3:61 [DATZ magazine, new imports from Jamanxim]. −Seidel & Evers [14]: 330 [figure and brief description. Jamanxim]. −Seidel [16]: 106, 107 [Fig 3. Jamanxim].
*Hopliancistrus* sp. "Rio Iriri". −Seidel & Evers [14]: 331. −Seidel [16]: 107 [Fig 5. Jamanxim].

**Fig 9:**
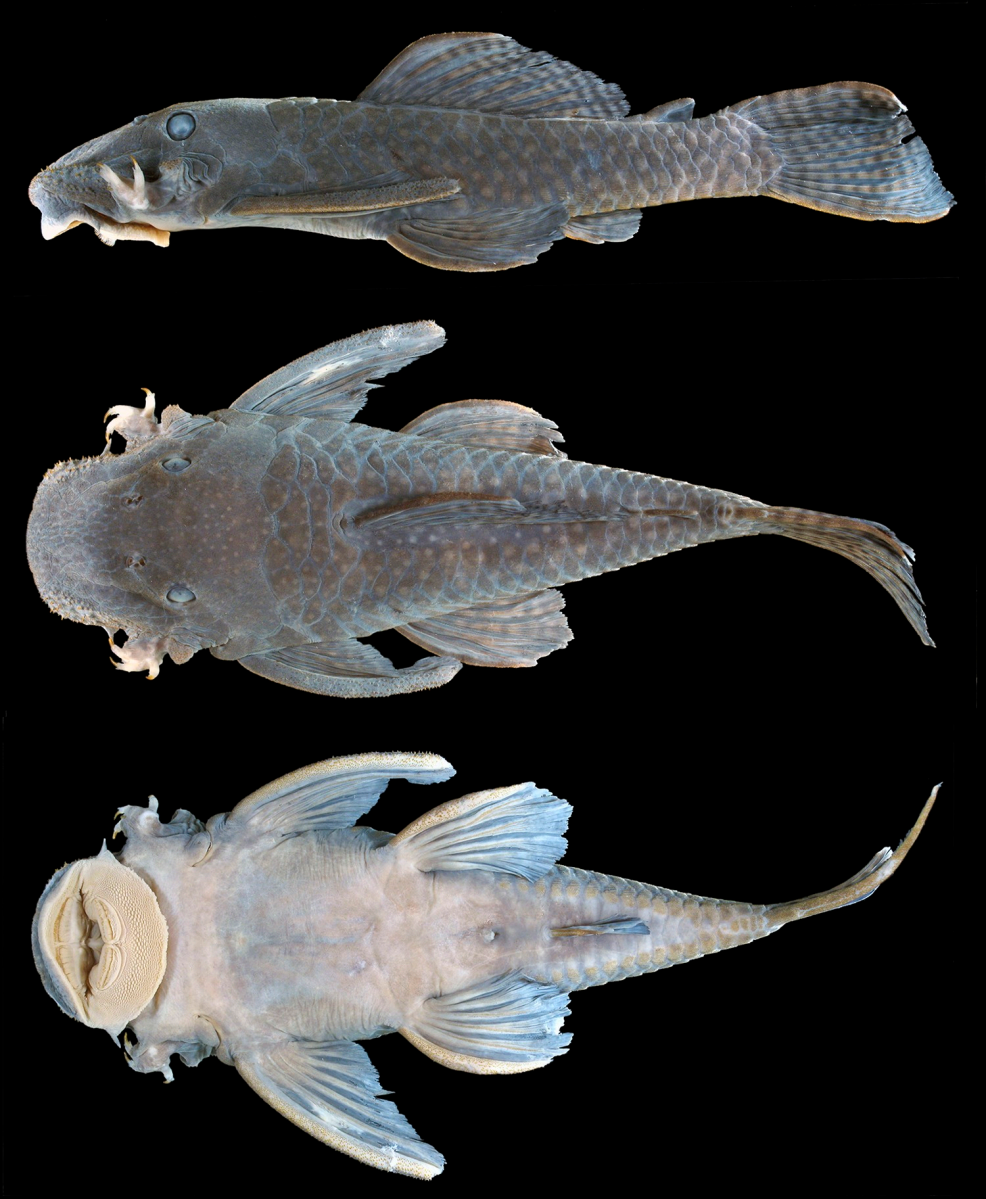
Holotype of *Hopliancistrus munduruku*. MZUSP 112207, 155.3 mm SL, from Rio Curuá, tributary of Rio Iriri, Rio Xingu basin.

#### Holotype

MZUSP 112207, 155.3 mm SL, Pará, Altamira, Curuá River, tributary of Iriri River, on Castelo dos Sonhos Village, 8°19’07”S 55°05’23”W, Xingu River basin, 22 Oct 2007, J. Birindelli, M. Sabaj-Pérez, L. M. Sousa, A. Netto-Ferreira & N. K. Lujan.

#### Paratypes

All from Brazil, Pará: **Altamira, Rio Xingu basin**: MZUSP 97209, 7, 55.4–155.3 mm SL, collected with holotype. ANSP 190689, 20, 23.0–108.4 mm SL; INPA 35272, 1 alc, 72.0 mm SL and 1 cs, 65.6 mm SL; MZUSP 96964, 25, 35.9–93.5 mm SL, Rio Curuá, tributary of Rio Iriri, below two large waterfalls, 8°43’50”S 54°57’49”W, 20 Oct 2007, J. Birindelli, M. Sabaj-Pérez, L. M. Sousa, A. Netto-Ferreira & N. K. Lujan. INPA 35271, 2, 73.8–86.8 mm SL; MZUSP 97113, 4, 49.8–93.4 mm SL, Rio Treze de Maio, tributary of Rio Curuá, Rio Iriri drainage, downstream of a large waterfall, 8°43’41”S 55°01’38”W, 22 Oct 2007, J. L. Birindelli, M. Sabaj-Pérez, L. M. Sousa, A. Netto-Ferreira & N. K. Lujan. INPA 38255, 1 alc, 103.1 mm SL, 1 skel, 73.3 mm SL, Igarapé on road BR 163, 9 Jun 2002, M. Camargo & T. Giarrizzo. MZUSP 96944, 6, 45.7–125.4 mm SL, Rio Treze de Maio, tributary of Rio Curuá, Rio Iriri drainage, at bridge of road BR 163, near Cachoeira da Serra Village, 8°38’53”S 55°01’41”W, 20 Oct 2007, J. Birindelli, M. Sabaj-Pérez, L. M. Sousa, A. Netto-Ferreira & N. K. Lujan. MZUSP 119674, 10, 72.4–124.6 mm SL, Rio Curuá, beach on Rio Curuá in Castelo dos Sonhos Village, 8°19’07”S 55°05’23”W, 7 Aug 2015, O. Oyakawa, W. M. Ohara & M. Pastana. **Novo Progresso, Rio Jamanxim, Rio Tapajós basin**: ANSP 190692, 5, 90.0–147.6 mm SL; INPA 35270, 1 alc, 112.2 mm SL and 1 cs, 74.0 mm SL; MZUSP 97359, 8, 53.3–124.5 mm SL, near Mil Village, 7°43’51”S 55°16’36”W, 23 Oct 2007, J. Birindelli, L. M. Sousa, A. L. Netto-Ferreira, M. Sabaj-Pérez & N. K. Lujan. MZUSP 97273, 4, 41.9–87.3 mm SL, ca. 30 km NW of Castelo dos Sonhos Village, 8°11’04”S 55°21’28”W, 23 Oct 2007, J. Birindelli, M. Sabaj-Pérez, L. M. Sousa, A. Netto-Ferreira & N. K. Lujan. MZUSP 97506, 1, 136.2 mm SL, little beach, near Novo Progresso Village, 7°03’52”S 55°26’28”W, 24 Oct 2007, J. Birindelli, M. Sabaj-Pérez, L. M. Sousa, A. Netto-Ferreira & N. K. Lujan.

#### Diagnosis

*Hopliancistrus munduruku* is distinguished from its congeners except *H*. *tricornis* by having large yellowish-white spots along the body, and dark brown spots on fins (vs. body covered by conspicuous small greenish-yellow dots of similar size on head, trunk and fins in *H*. *wolverine*; yellowish-white spots on posterior portion of the body moderate in size, usually smaller than pupil in *H*. *xikrin*; all fins covered by large yellowish-white spots in *H*. *xavante*). *Hopliancistrus munduruku* can be distinguished from *H*. *tricornis* and *H*. *wolverine* by the connection strut between the anterior process of the compound pterotic and main body shaped as a continuous sheet (vs. connection strut narrow and bar-shaped, leaving a large posterior gap, see Fig 6). *Hopliancistrus munduruku* also differs from *H*. *tricornis* by the possession of five branched anal-fin rays (vs. four), and from *H*. *wolverine* and *H*. *xikrin* by pectoral-fin spine length 24.5–30.9% of SL (vs. 32.1–38.4% of SL and 32.1–35.7% of SL, respectively). It differs from *H*. *xikrin* by the transverse processes of first and second dorsal-fin pterygiophores sutured to each other (vs. absence of contact between the transverse processes of first and second dorsal-fin pterygiophores). It differs from *H*. *xavante* by caudal peduncle depth 10.1–11.3% of SL (vs. 11.5–12.9% of SL); by a narrow nasal bone plate (vs. broad nasal, sometimes slightly triangular, see Fig 2); and by having nuchal plate exposed and covered by odontodes (vs. nuchal plate covered by thick skin and usually lacking odontodes, see Fig 7).

#### Description

Body shape and pigmentation in Figs 1A, 5B and 9. Morphometric data and counts in Table 1. Small-sized loricariid, with largest examined specimen reaching 155.3 mm SL. Head roughly square anteriorly in dorsal view; gradually narrowing from cleithrum to caudal-fin origin. Greatest width of body at cleithrum. Head and trunk wide and depressed, greatest depth at dorsal-fin origin. In lateral view, dorsal profile slightly convex from snout tip to dorsal-fin origin, gently declining from dorsal-fin origin to first procurrent caudal-fin ray. Ventral profile straight from snout tip to caudal-fin origin.

Body anterior region roughly trapezoidal, depressed dorsoventrally, and slightly oval at caudal peduncle in cross section. Weak ridge from snout tip to anterior nare and from posterior nares to eye. Snout completely covered by plates except for small naked area on its tip. Lateral border of snout covered by short stiff odontodes. First four plates of mid-ventral series gently keeled. Ventral surface of caudal peduncle flat; small and delicate keel at three or four last ventral plates series of caudal peduncle. Remaining of body plates not keeled.

Head large and wide. Eye rounded and moderate in size, dorsolaterally positioned at midpoint of head length; iris operculum present. Orbit not elevated; interorbital area nearly straight. Parieto-supraoccipital almost indistinct from rest the other bones of the skull. Supraoccipital process not elevated, slightly rounded posteriorly. Parieto-supraoccipital limited posteriorly by four roughly triangular plates. Predorsal area with three pairs of predorsal plates, plus one very small nuchal plate anterior to dorsal-fin spinelet.

Mouth and lips of moderate size; oral disk elliptical; lips almost completely covered with small round papillae densely packed, slightly larger on central portion of lower lip; lips area surrounding premaxillary and dentary smooth, without papillae. Lower lip large, but not reaching pectoral girdle. Maxillary barbel short and with small portion free from lower lip. Teeth short, thin, delicate, and bicuspid. Mesial cusp slightly larger and wider than lateral cusp; lateral cusp reaching three-quarters of mesial cusp length. Premaxilla and dentary of similar size and disposed parallel to anterior border of snout. Single very short buccal papilla between premaxilla.

Dorsal body surface completely covered by large plates, except immediately around dorsal-fin base. Ventral surface entirely devoid of plates from snout to anal-fin origin. Base of first anal-fin pterygiophore covered by skin, preanal plate absent. Caudal peduncle completely covered by plates. Median series plates 23–25. Five series of lateral plates at caudal peduncle. Six to seven oblong plates on caudal-fin base. Opercle exposed and covered by odontodes, exposed part sickle-shaped; plates immediately posterior to opercle small, sometimes reaching one-third of length of exposed opercle. Plates of supraopercular area small and few in number, leaving large naked area around opercle. Three large, strong, and curved odontodes on cheek plates, reaching posterior margin of branchial opening; fleshy and thick odontodes sheath, sometimes covering two-thirds of cheek odontodes. All plates covered with small and aligned odontodes; odontodes slightly larger on opercle, postopercular plate and pectoral-fin spine.

Dorsal-fin origin on anterior portion of body, slightly anterior to vertical through pelvic-fin origin. Dorsal-fin II,7; spinelet present and very reduced, dorsal-fin locking mechanism not functional. Dorsal fin short and low, not reaching adipose-fin when adpressed; reaching pre-adipose plate in some individuals. Three (2), four (24) and five (12) plates separating dorsal from adipose fin. Adipose fin short and low. Two unpaired dorsal plates between adipose fin and first procurrent caudal-fin ray. Caudal-fin i,14,i, slightly emarginated, with oblique distal margin, ventral lobe slightly longer. Pectoral fin I,6, large, reaching urogenital opening when adpressed. Pectoral-fin spine thick and strong, not pungent, covered by large odontodes on mature males. Pelvic fin i,5 reaching end of anal-fin base when adpressed. Anal fin i,5, moderate in size, reaching insertion line of adipose-fin spine. All rays covered by numerous short odontodes on their free surface. Anteriormost branched rays of pelvic and anal fins slightly larger than unbranched rays. Four to five dorsal and ventral procurrent caudal-fin rays. Vertebrae 28 (3). Eight (3) rib pairs.

#### Color in alcohol

Very similar to *Holiancistrus tricornis* in preserved specimens. Background color dark brown at head, dorsum and sides. Head covered by whitish-yellow small spots and trunk with irregular whitish-yellow blotches, sometimes present in trunk of few large specimens. All fins light brown covered with brown to black wing-like marks (as *H*. *tricornis*). Ventral surface of pectoral and pelvic fins cream colored without spots. Ventral surface of body uniformly cream to whitish-yellow (Fig 9).

#### Color in life

Background color gray or olive green at head, dorsum, and sides. Head covered by small yellow spots and trunk with irregular yellow blotches. All fins whitish-yellow, covered by small dark gray to black spots over rays of dorsal, caudal, and anal, and on dorsal surface of pectoral and pelvic fins. Distal portion of caudal-fin lobes yellow, more conspicuous in juvenile specimens. Ventral surface yellowish-white without dots (Fig 5B).

#### Distribution

*Hopliancistrus munduruku* is currently known from upper Rio Jamanxim, Rio Tapajós basin, and upper Rio Curuá, tributary of Rio Iriri, Rio Xingu basin, in the Novo Progresso municipality, Pará State (Fig 8). This disjunct distribution between upper tributaries to Tapajós and Xingu basin suggests, like in other taxa, drainage capture by geologic events.

#### Etymology

The new species is named after the Munduruku, a large indigenous group inhabiting a large part of the southwestern Pará State along the Rio Tapajós until the Rio Madeira in the Amazon State and the northern part of the Mato Grosso State in Brasil. The Munduruku people are well known for being powerful warriors and great strategists, and in recent years they have drawn much attention for the fight against the hydroelectric dams in the Xingu and the ones planned in the Tapajós Rivers.

### *Hopliancistrus wolverine*, new species

**Fig 10:**
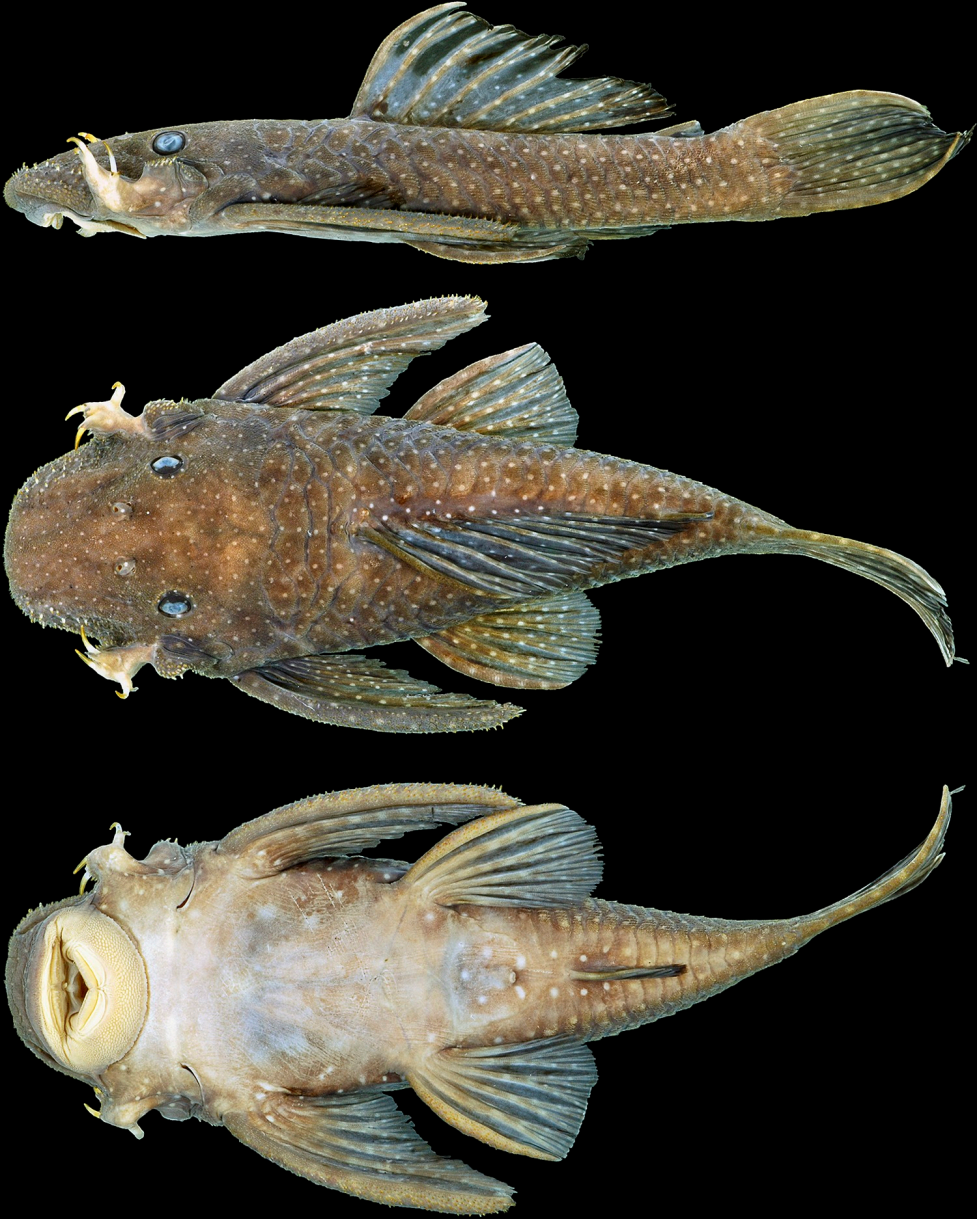
Holotype of *Hopliancistrus wolverine*. INPA 4012, 134.3 mm SL, from Volta Grande, Rio Xingu.

**Fig 11:**
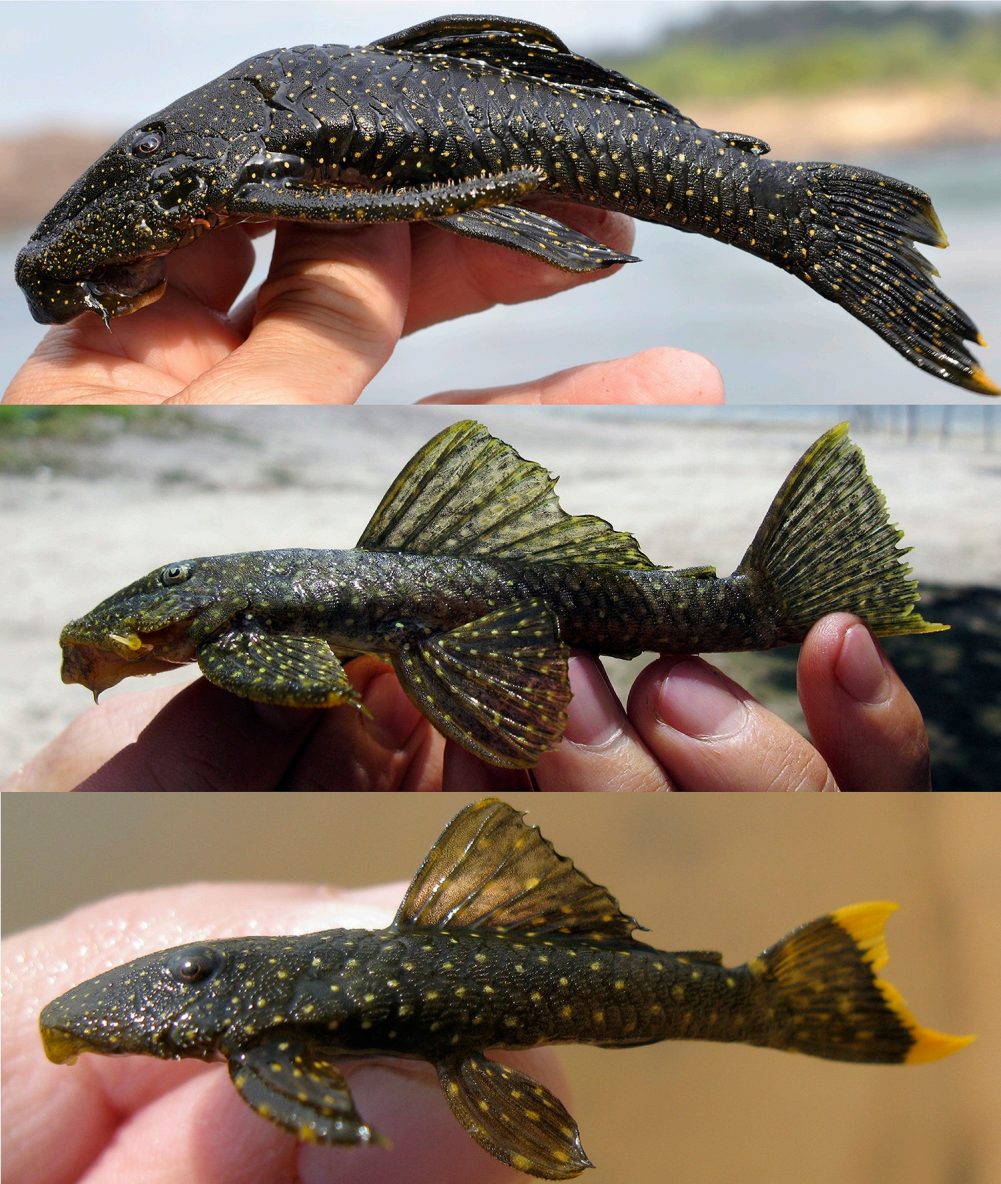
Lateral view of living specimens of *Hopliancistrus wolverine*. (above) INPA 38036, 138.0 mm SL, paratype; (middle) MNRJ 35607, 92.3 mm SL; (below) uncatalogued specimen. Photographed by M. Sabaj and L. Sousa.

**Fig 12:**
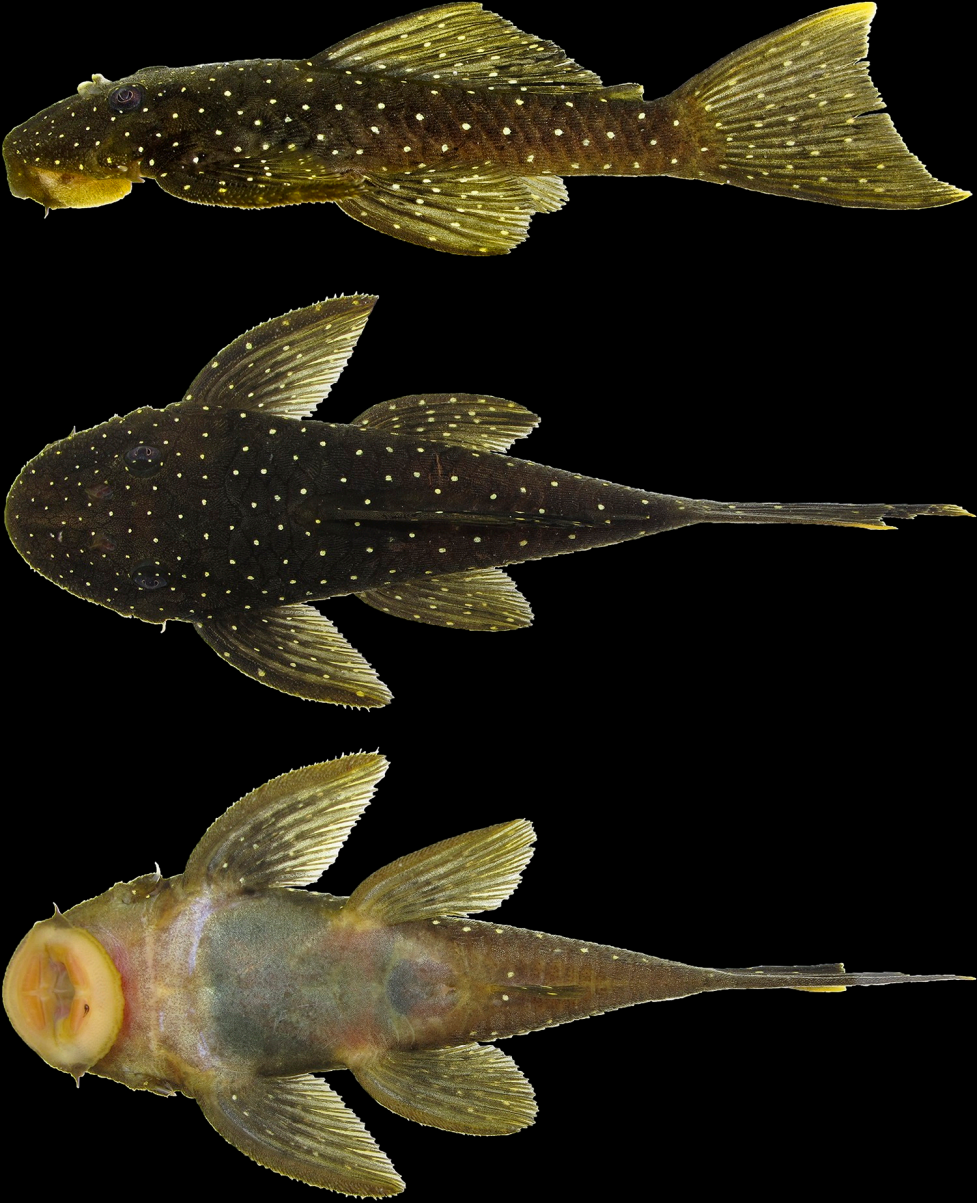
*Hopliancistrus wolverine* showing three views of living color. INPA 52359, 67.2 mm SL, Rio Xingu. Photographed by L. Sousa.

urn:lsid:zoobank.org:act:66CFEF13-4580-4778-A129-66282CF32606

(Figs 1B, 5C and 10–12)

*Hopliancistrus* sp. (L17). −Seidel & Evers [14]: 324 [L17 was originally depicted by Stawikowski [20]: 174 [Fig 9], showing a *Pseudancistrus* later described as *P*. *asurini*].
*Hopliancistrus* sp. (LDA15). −Seidel & Evers [14]: 326, 327, 328. −Seidel [16]: 107 [Fig 4].
*Hopliancistrus* sp. "Rio Xingú". −Seidel & Evers [14]: 332. −Seidel [16]: 107 [Fig 6]
*Hopliancistrus* sp. 'L017'. −Seidel [16]: 106,107 [Fig 2]
*Hopliancistrus* cf. *tricornis*. −Camargo & Ghilard Jr. [21]: 239 [list of ornamental fishes captured in Xingu].
*Hopliancistrus* sp. −Castilhos & Buckup [22]: 172 [Ichthyofaunal inventory of Xingu and Tapajós. Fig 7–7 M].
*Hopliancistrus* sp. 1 (L17). −Camargo et al. [15]: 157.

#### Holotype

INPA 4012, 134.3 mm SL, Brazil, Pará, Altamira, Rio Xingu, Pimental Furo da Crente, 3°27’30”S 51°54’41”W, 17 Sep 1997, J. Zuanon.

#### Paratypes

All from Brazil, Pará, Rio Xingu basin: ANSP 193023, 1, 130.8 mm SL; INPA 38036, 1, 138.0 mm SL, Altamira, Rio Iriri, Cachoeira Grande do Iriri, 3°50’36”S 52°44’08”W, 10 Oct 2012, M. Sabaj-Pérez, L. M. Sousa & M. Arce. ANSP 194663, 8, 49.3–148.4 mm SL; INPA 40052, 7, 41.2–121.9 mm SL, Altamira, Rio Iriri, immediately below Cachoeira Grande do Iriri, ca. 15 km upstream from confluence with Rio Xingu, 3°50’34.62”S 52°44’10.74”W, 9 Sep 2013, M. Sabaj-Pérez, L. M. Sousa, A. Gonçalves, N. K. Lujan, D. Fitzgerald & P. M. Ito. ANSP 195938, 2, 47.9–91.7 mm SL; INPA 43427, 2, 57.1–68.6 mm SL, Altamira, Rio Xingu, middle Volta Grande, ca. 55 km of Altamira, 3°33’41.1”S 51°51’29.1”W, 10 Mar 2014, M. Sabaj-Pérez, L. Rapp Py-Daniel, R. R. de Oliveira, M. Arce, A. Gonçalves & D. Fitzgerald. ANSP 196913, 1, 89.8 mm SL; INPA 43591, 1, 56.1 mm SL, Altamira, Rio Iriri, Cachoeira Grande do Iriri, 3°51’19”S 52°43’44”W, 16 Mar 2014, M. Sabaj-Pérez, L. Rapp Py-Daniel, R. R. de Oliveira, M. Arce, A. Gonçalves & D. Fitzgerald. ANSP 196926, 4, 62.2–99.8mm SL; INPA 43731, 6 alc, 46.2–83.5mm SL and 1 skel, 122.2 mm SL, Altamira, Rio Iriri, immediately below of Cachoeira Grande do Iriri, shallow rapids on left margin, 3°50’46”S 52°43’20”W, 15 Mar 2014, M. Sabaj-Pérez, L. Rapp Py-Daniel, R. R. de Oliveira, M. Arce, A. Gonçalves & D. Fitzgerald. ANSP 197869, 1, 142.9 mm SL; INPA 47541, 1, 56.7 mm SL, Rio Iriri, Cachoeira Grande do Iriri, ca. 15 km upstream from confluence with Rio Xingu, 3°50’32.3”S 52°44’03.9”W, 2 Nov 2014, M. Sabaj-Pérez, L. M. Sousa, R. R. de Oliveira, A. Gonçalves, D. Fitzgerald, V. Machado, P. M. Ito. ANSP 200877, 2, 119.4–181.8 mm SL, Altamira, Rio Iriri, Cachoeira Grande do Iriri, ca. 15 km upstream from confluence with Rio Xingu, 3°50’36.4” 52°44’4.2”W, 22 Sep 2015, M. Sabaj-Pérez, L. M. Sousa, M. Kalacska, J. P. Arroyo, O. Lucanus & Fishemen. INPA 31447, 1, 46.7 mm SL, Altamira, Rio Xingu, point of Aba Laranja, Community do Maia, 3°31’42”S 51°45’02”W, 9 Nov 2008, L. Rapp Py-Daniel & R. R. de Oliveira. INPA 31823, 1, 64.1 mm SL, Altamira, Rio Xingu, rocks in front of the Community do Maia, 3°30’44”S 51°44’43”W, 9 Nov 2008, L. Rapp Py-Daniel & R. R. de Oliveira. INPA 34659, 1, 141.9 mm SL, Altamira, Rio Xingu, Kaituká rapids, 10 Oct 1990, L. Rapp Py-Daniel & J. Zuanon. INPA 34950, 1, 47.4 mm SL, Altamira, Rio Xingu, Arini rapids, 3°24’15”S 51°41’53”W, 8 Sep 1997, J. Zuanon. INPA 35275, 2, 78.4–85.7 mm SL, Altamira, Rio Xingu, Cachoeira de Kaituká, 5 Jan 2001, M. Zorro. INPA 39161, 1, 141.3 mm SL, Altamira, Igarapé Santo Antônio, tributary of Rio Xingu, 3°08’38”S 51°47’50”W, 18 Nov 2012, D. A. Bastos & A. Jamesson. INPA 40199, 1, 60.9 mm SL, Anapu, Rio Xingu, 1 km above Cachoeira de Jericoá, 3°21’56”S 51°43’36”W, 14 Sep 2013, M. Sabaj-Pérez, L. M. Sousa, A. S. Oliveira & P. M. Ito. INPA 40842, 1, 39.5 mm SL, Anapu, Rio Xingu, below Cachoeira Tamaracá, right margin, 3°07’41”S 51°37’17”W, 1 Oct 2013, M. Sabaj-Pérez, L. M. Sousa, A. S. Oliveira & P. M. Ito. INPA 40873, 11, 43.7–148.0 mm SL, Vitória do Xingu, Igarapé Belo Monte, tributary of Rio Xingu, 3°06’46”S 51°37’42”W, 16 Jul 2012, L. M. Sousa & R. Fróis. INPA 40874, 1, 43.1 mm SL, Vitória do Xingu, Rio Xingu, Belo Monte, 3°07’43”S 51°39’53”W, 16 Jul 2012, L. M. Sousa & R. Fróis. INPA 40875, 4 alc, 103.6–136.3 mm SL and 1 skel, 136.1 mm SL, Altamira, Rio Iriri, Cachoeira Grande do Iriri, 3°50’36”S 52°44’07”W, 14 Sep 2012, L. M. Sousa & J. Santos. INPA 40876, 8, 40.4–114.8 mm SL, Altamira, Rio Iriri, Cachoeira Grande do Iriri, 3°50’37”S 52°44’01”W, 2 Jul 2012, L. M. Sousa & R. Fróis. INPA 40877, 1, 135.1 mm SL, Altamira, Rio Xingu, Cachoeira do Espelho, 3°38’42”S 52°22’54”W, 4 Jul 2012, L. M. Sousa & R. Fróis. INPA 40878, 1, 42.1 mm SL, Altamira, Rio Xingu, Cajueiro, 3°16’34”S 52°05’08”W, 18 Sep 2012, D. A. Bastos & J. Santos. INPA 40879, 1, 40.3 mm SL, Altamira, Rio Iriri, Cachoeira Grande do Iriri, 3°50’36”S 52°44’07”W, 21 Sep 2012, L. M. Sousa & J. Santos. INPA 43514, 1, 109.6 mm SL, Altamira, Rio Xingu, ca. 21 km below from Altamira, 3°18’36”S 52°02’50”W, 12 Mar 2014, M. Sabaj-Pérez, L. Rapp Py-Daniel, R. R. de Oliveira, M. Arce, A. Gonçalves & D. Fitzgerald. INPA 48031, 1 alc, 108.3 mm SL and 1 skel, 92.4 mm SL, Altamira, Rio Iriri, Community Manelito 2, RESEX do Iriri, 4°46’51”S 54°38’42”W, 13 Aug 2012, D. B. Aviz. INPA 52276, 4, 49.1–125.0 mm SL, Altamira, Rio Iriri, 3°52’5”S 52°43’3”W, 25 Aug 2012, E. D. Ribeiro & L. M. Sousa. INPA 52308, 1, 84.4 mm SL, Altamira, Rio Xingu, Cachoeira do Jericoá, 3°21’57”S 51°44’8”W, 1 Sept 2012, E. D. Ribeiro & L. M. Sousa. INPA 52359, 2, 66.2–67.2 mm SL, Altamira, Rio Xingu, rapids above of Cachoeira de Belo Monte, 3°6’49”S 51°37’59”W, 6 Sept 2012, E. D. Ribeiro & L. M. Sousa. LIA 1162, 1, 42.7 mm SL, São Félix do Xingu, Xadai, Rio Xingu, above São Félix do Xingu, 6°53’35.5”S 52°2’14.5”W, 20 Sept 2014, L. Sousa, A. Gonçalves, C. Martins and fishermen Daildo, Neto and Quinha. LIA 1220, 5, 35.3–40.9 mm SL, São Félix do Xingu, Rio Xingu, Pedra Preta, 6°34’43.2”S 52°10’40.0”W, 22 Sept 2014, L. Sousa, A. Gonçalves, C. Martins and fishermen Daildo, Neto and Quinha. LIA 1304, 1, 36.6 mm SL, São Félix do Xingu, Rio Xingu, Pedra Preta, 6°34’34.5”S 52°10’46.7”W, 22 Sept 2014, L. Sousa, A. Gonçalves, C. Martins and fishermen Daildo, Neto and Quinha. LIA 1386, 1, 76.3 mm SL, São Félix do Xingu, Rio Xingu, Onça, 7°7’27.4”S 52°30’16.1”W, 21 Sept 2014, L. Sousa, A. Gonçalves, C. Martins and fishermen Daildo, Neto and Quinha. LIA 1462, 1, 42.5 mm SL, São Félix do Xingu, Rio Xingu, Araraquara, 6°33’16.1”S 52°6’43.1”W, 25 Sept 2014, L. Sousa, A. Gonçalves, C. Martins and fishermen Daildo, Neto and Quinha. LIA 1526, 3, 36.4–87.5 mm SL, São Félix do Xingu, Rio Xingu, Travessão do Nazaré, 6°18’4.3”S 52°28’50.8”W, 26 Sept 2014, L. Sousa, A. Gonçalves, C. Martins and fishermen Daildo, Neto and Quinha. LIA 2377, 2, 52.1–75.8 mm SL, São Félix do Xingu, Rio Xingu, Parna Serra do Pardo, 5°46’15.8”S 52°37’14.5”W, 18 May 2015, A. Gonçalves, D. Silva and fishermen Ney, Toninho, Sérgio and Fábio. LIA 2402, 1, 22.9 mm SL, São Félix do Xingu, Rio Xingu, Ilha do Pontal, 5°42’17.5”S 52°39’49.6”W, 19 May 2015, A. Gonçalves, D. Silva and fishermen Ney, Toninho, Sérgio and Fábio. LIA 2427, 3, 47.5–78.7 mm SL, São Félix do Xingu, Rio Xingu, Raio do Sol, PARNA Serra do Pardo, 5°52’3.3”S 52°33’29.0”W, 20 May 2015, A. Gonçalves, D. Silva and fishermen Ney, Toninho, Sérgio and Fábio. LIA 2454, 2, 49.1–59.2 mm SL, São Félix do Xingu, Rio Xingu, São Sebastião, PARNA Serra do Pardo, 5°47’46.5”S 52°34’41.7”W, 21 May 2015, A. Gonçalves, D. Silva and fishermen Ney, Toninho, Sérgio and Fábio. LIA 2467, 1, 63.4 mm SL, São Félix do Xingu, Rio Xingu, 100 m above of camp, 5°47’46.5”S 52°34’41.7”W, 21 May 2015, A. Gonçalves, D. Silva and fishermen Ney, Toninho, Sérgio and Fábio. LIA 2479, 3, 58.6–61.9 mm SL, São Félix do Xingu, Rio Xingu, stone wall below Parna headquarters, 5°46’15.8”S 52°37’14.5”W, 21 May 2015, A. Gonçalves, D. Silva and fishermen Ney, Toninho, Sérgio and Fábio. LIA 2494, 2, 36.7–49.4 mm SL, São Félix do Xingu, Rio Xingu, Pedra Preta, 6°57’31.6”S 52°17’12.8”W, 23 May 2015, A. Gonçalves, D. Silva and fishermen Ney, Toninho, Sérgio and Fábio. LIA 2505, 1, 57.6 mm SL, São Félix do Xingu, Rio Xingu, Pedra Preta, 6°57’29.7”S 52°15’30.2”W, 23 May 2015, A. Gonçalves, D. Silva and fishermen Ney, Toninho, Sérgio and Fábio. LIA 2514, 2, 49.5–82.0 mm SL, São Félix do Xingu, Rio Xingu, Pedra Preta, 6°57’8.9”S 52°13’48.5”W, 23 May 2015, A. Gonçalves, D. Silva and fishermen Ney, Toninho, Sérgio and Fábio. LIA 2620, 1, 53.5 mm SL, São Félix do Xingu, Rio Xingu, Camp 5, 6°57’7.8”S 52°13’13.7”W, 25 May 2015, A. Gonçalves, D. Silva and fishermen Ney, Toninho, Sérgio and Fábio. LIA 2624, 2, 94.2–107.1 mm SL, São Félix do Xingu, Rio Xingu, Xadai, 6°52’35.0”S 52°2’9.2”W, 26 May 2015, A. Gonçalves, D. Silva and fishermen Ney, Toninho, Sérgio and Fábio. LIA 2638, 5, 53.5–85.4 mm SL, São Félix do Xingu, Rio Xingu, Xadai, 6°53’1.4”S 52°1’28.4”W, 26 May 2015, A. Gonçalves, D. Silva and fishermen Ney, Toninho, Sérgio and Fábio. LIA 2663, 2, 50.2–109.4 mm SL, São Félix do Xingu, Rio Xingu, Xadai, 6°53’44.2”S 52°2’35.8”W, 26 May 2015, A. Gonçalves, D. Silva and fishermen Ney, Toninho, Sérgio and Fábio. LIA 2690, 2, 76.1–135.4 mm SL, São Félix do Xingu, Rio Xingu, 3,6 km above Xadai, 6°53’39.5”S 52°3’58.4”W, 26 May 2015, A. Gonçalves, D. Silva and fishermen Ney, Toninho, Sérgio and Fábio. LIA 2858, 10, 47.8–119.9 mm SL, São Félix do Xingu, Rio Xingu, near Igarapé Triunfo, 6°53’39.5”S 52°3’58.4”W, 2 Jun 2015, A. Gonçalves, D. Silva and fishermen Ney, Toninho, Sérgio and Fábio. LIA 2869, 1, 77.7 mm SL, São Félix do Xingu, Rio Xingu, rocks, nearly 5 km below camp 7, 6°181’56.1”S 52°25’20.0”W, 2 Jun 2015, A. Gonçalves, D. Silva and fishermen Ney, Toninho, Sérgio and Fábio. LIA 3484, 2, 99.8–130.6 mm SL, Altamira, Rio Iriri, São Francisco/ RESEX do Rio Iriri, 4°25’9.8”S 53°48’15.3”W, 31 Jan 2016, A. Gonçalves, D. Silva, P. Trindade and fishermen Ronca, Dani and Fábio. LIA 3616, 1, 94.5 mm SL, Altamira, Rio Iriri, Mata Fome/ RESEX do Rio Iriri, 4°31’42.5”S 53°58’3.8”W, 28 Jan 2016, A. Gonçalves, D. Silva, P. Trindade and fishermen Ronca, Dani and Fábio. LIA 3761, 2, 48.5–72.9 mm SL, Altamira, Rio Xingu, Balisa/ RESEX do Rio Xingu, 4°22’41.2”S 52°42’47.0”W, 29 Feb 2016, A. Gonçalves, D. Silva, and fishermen Ronca and Fábio. LIA 3870, 1, 78.0 mm SL, Altamira, Rio Xingu, Estragado/ RESEX do Rio Xingu, 4°44’14.8”S 52°47’27.7”W, 23 Feb 2016, A. Gonçalves, D. Silva, and fishermen Ronca and Fábio. LIA 3933, 2, 42.6–73.0 mm SL, Altamira, Rio Xingu, Gabiroto/ RESEX do Rio Xingu, 5°8’7.9”S 52°54’34.7”W, 19 Feb 2016, A. Gonçalves, D. Silva, and fishermen Ronca and Fábio. LIA 3973, 1, 87.8 mm SL, Altamira, Rio Xingu, Bom Jardim/ RESEX do Rio Xingu, 5°35’15.7”S 52°42’39.9”W, 15 Feb 2016, A. Gonçalves, D. Silva, and fishermen Ronca and Fábio. LIA 3987, 1, 127.6 mm SL, Altamira, Rio Xingu, Bom Jardim/ RESEX do Rio Xingu, 5°29’51.4”S 52°43’51.8”W, 15 Feb 2016, A. Gonçalves, D. Silva, and fishermen Ronca and Fábio. LIA 5010, 1, 127.6 mm SL, Altamira, Rio Xingu, Bom Jardim/ RESEX do Rio Xingu, 5°29’51.4”S 52°43’51.8”W, 15 Feb 2016, A. Gonçalves, D. Silva, and fishermen Ronca and Fábio. LIA 5878, 1, 103.1 mm SL, Vitória do Xingu, Rio Xingu, reduced flow section, Casa Branca, 3°20’20.2”S 51°45’2.5”W, 8 Aug 2016, L. Sousa, A. Netto-Ferreira, M. Sabaj & M. S. Sabaj. LIA 5930, 2, 92.6–104.5 mm SL, Altamira, Rio Iriri, Lajeiro, RESEX do Rio Iriri, 4°51’29.2”S 54°36’52.5”W, 22 Aug 2016, A. Gonçalves, D. Silva, D. Batista & N. Estrela. LIA 5953, 1, 103.7 mm SL, Altamira, Rio Iriri, RESEX do Rio Iriri, Curral de Pedra, 4°34’38.4”S 54°2’41.7”W, 25 Aug 2016, A. Gonçalves, D. Silva, D. Batista & N. Estrela. LIA 5980, 2, 95.8–99.0 mm SL, Altamira, Rio Iriri, Furo do Veado, São Francisco, RESEX do Rio Iriri, 4°25’32.4”S 53°48’13.3”W, 28 Aug 2016, A. Gonçalves, D. Silva, D. Batista & N. Estrela. LIA 6011, 1, 93.5 mm SL, Altamira, Rio Iriri, Boca do João Cândido, RESEX do Rio Iriri, 31 Aug 2016, A. Gonçalves, D. Silva, D. Batista & N. Estrela. LIA 6075, 1, 87.8 mm SL, Altamira, Rio Xingu, Gabiroto, RESEX do Rio Xingu, 4°59’31.4”S 52°55’16.8”W, 21 Sep 2016, A. Gonçalves, D. Silva, P. Trindade & P. Ito. LIA 6100, 1, 76.7 mm SL, Altamira, Rio Xingu, RESEX Xingu, Cachoeira da Mucura, 4°56’36.4”S 52°52’53.5”W, 23 Sep 2016, A. Gonçalves, D. Silva, P. Trindade & P. Ito. LIA 6155, 1, 57.9 mm SL, Altamira, Rio Xingu, RESEX Xingu, Volta da Pedra/Furo da Cachoeira, 4°45’8.1”S 52°47’32.1”W, 25 Sep 2016, A. Gonçalves, D. Silva, P. Trindade & P. Ito. LIA 6178, 1, 58.7 mm SL, Altamira, RESEX Xingu, Pedra Preta/Furo do Jatobá, 4°32’22.6”S 52°42’49.4”W, 27 Sep 2016, A. Gonçalves, D. Silva, P. Trindade & P. Ito. LIA 6294, 1, 47.8 mm SL, São Félix do Xingu, Rio Xingu, lateral channel in the Corredeira do Onça, 7°7’35.0”S 52°31’37.0”W, 12 Aug 2016, L. Sousa, O. Lucanus, M. Kalacska & D. Costa. LIA 6304, 1, 40.3 mm SL, São Félix do Xingu, Rio Xingu, Cachoeira do Acadá, 6°20’44.0”S 52°25’19.0”W, 14 Aug 2016, L. Sousa, O. Lucanus, M. Kalacska & D. Costa. LIA 6330, 2, 50.9–125.4 mm SL, São Félix do Xingu, Rio Xingu, Cachoeira do Urubu, 6°13’22.0”S 52°29’31.0”W, 9 Aug 2016, L. Sousa, O. Lucanus, M. Kalacska & D. Costa. LIA 6333, 1, 85.6 mm SL, Altamira, Rio Xingu, Cachoeira do Espelho, 3°39’6.0”S 52°22’43.0”W, 27 Aug 2016, L. Sousa, A. Netto-Ferreira, M. Sabaj & M. S. Sabaj. MCP 54540, 2, 72.7–92.2 mm SL, São Félix do Xingu, Rio Xingu, Pedra Preta, 6°56’2.1”S 52°8’13.5”W, 22 Sept 2014, L. Sousa, A. Gonçalves, C. Martins and fishermen Daildo, Neto and Quinha. MPEG 38947, 5, 40.7–100.4 mm SL, Altamira, Rio Xingu, Rebojo do Avelino, 3°45’6.4”S 52°30’44.1”W, 29 Sept 2014, L. Sousa, A. Gonçalves, C. Martins and fishermen Daildo, Neto and Quinha. MZUEL 20812, 4, 55.8–114.9 mm SL, São Félix do Xingu, Rio Xingu, Ilha do Pontal, 5°43’12.8”S 52°39’54.9”W, 19 May 2015, A. Gonçalves, D. Silva and fishermen Ney, Toninho, Sérgio and Fábio. MZUSP 34287, 2, 108.6–135.0 mm SL, Altamira, Igarapé Santo Antônio, tributary of Rio Xingu, 3°07’00”S 51°42’00”W, 27 Sep 1983, M. Goulding. NUP 22272, 2, 47.5–117.3 mm SL, São Félix do Xingu, Rio Xingu, São Sebastião, PARNA Serra do Pardo, 5°48’5.2”S 52°34’25.7”W, 20 May 2015, A. Gonçalves, D. Silva and fishermen Ney, Toninho, Sérgio and Fábio. ZUEC 4503, 2, 41.8–91.4 mm SL, Altamira, Rio Xingu, Corredeira na ilha do Arini, 3°24’15”S 51°41’53”W, 7 Nov 1997, J. Zuanon. ZUEC 17326, 2, 75.5–76.2 mm SL, Altamira, RESEX Xingu, Baliza, 4°24’3.7”S 52°40’57.1”W, 29 Sep 2016, A. Gonçalves, D. Silva, P. Trindade & P. Ito.

#### Non-types

INPA 34658, 1 alc, 108.5 mm SL and 1 cs, 71.2 mm SL, Altamira, Rio Xingu, Corredeiras do Arini, 3°24’15”S 51°41’53”W, 7 Sep 1997, J. Zuanon. MNRJ 35607, 1, 92.3 mm SL, Brazil, Pará, Altamira, material from Hom Aquarium, Rio Xingu, 25 Nov 2008.

#### Diagnosis

*Hopliancistrus wolverine* is distinguished from its congeners by having body color black, dark olive, or dark gray covered by conspicuous greenish-yellow small dots of similar size on head, trunk, and fins (vs. head with small spots, and large whitish-yellow spots or blotches posteriorly towards caudal fin, and dorsal, caudal, and anal fins covered by dark brown spots over rays). *Hopliancistrus wolverine* can be further distinguished from its congeners by a combination of additional characters: from its congeners except *H*. *tricornis* by a narrow, bar-shaped connection strut between anterior process of compound pterotic and main body, leaving a large posterior gap (vs. connection strut shaped as a continuous sheet, see Fig 6). Additionally, *Hopliancistrus wolverine* is distinguished from its congeners except *H*. *xikrin* by pectoral-fin spine length 32.1–38.4% of SL (vs. 25.1–29.9% in *H*. *tricornis*, 24.5–30.9% in *H*. *munduruku* and 27.4–30.8% of SL in *H*. *xavante*). It differs from *H*. *xikrin* by the transverse processes of first and second dorsal-fin pterygiophores sutured to each other (vs. absence of contact between the transverse processes of first and second pterygiophores). *Hopliancistrus wolverine* differs from *H*. *xavante* by having narrow nasal bone plate (vs. nasal broad, sometimes slightly triangular, see Fig 2); and by having the nuchal plate exposed and covered by odontodes (vs. nuchal plate covered by thick skin and usually lacking odontodes, see Fig 7). *Hopliancistrus wolverine* also differs from *H*. *tricornis* by the possession of five branched rays on the anal-fin (vs. four).

#### Description

Body shape and pigmentation in Figs 1B, 5C and 10–12. Morphometric data and counts in Table 2. Small-sized loricariid, with largest examined specimen 181.8 mm SL. Head roughly square anteriorly in dorsal view; body gradually narrowing from cleithrum to caudal-fin origin. Greatest width of body at cleithrum. Head and trunk wide and very depressed, greatest depth at dorsal fin origin. In lateral view, dorsal profile slightly convex from snout tip to dorsal-fin origin, then straight or flat to caudal-fin origin. Ventral profile straight from snout tip to caudal-fin origin.

**Table 2:** Morphometric data and counts of *Hopliancistrus wolverine* and *H*. *xikrin* from Rio Xingu basin.

	*Hopliancistrus wolverine*						*Hopliancistrus xikrin*					
Measurement	H	Mean	Min	Max	SD	N	H	Mean	Min	Max	SD	N
Standard length	134.3		62.2	181.8		44	131.3		65.0	141.6		35
**% of standard length**												
Predorsal length	43.3	43.9	41.1	46.3	1.2	44	44.5	44.6	43.4	46.0	0.7	35
Head length	36.3	35.5	33.4	37.0	0.9	44	35.4	36.0	34.9	37.7	0.6	35
Cleithral width	36.5	34.4	32.7	36.6	1.0	44	34.5	35.4	34.1	37.7	0.7	35
Cleithral process width	33.8	31.9	30.4	33.8	0.8	44	31.9	33.0	31.3	35.0	0.7	35
Pectoral-Pelvic origin length	25.7	25.1	22.2	28.5	1.5	44	23.9	25.1	22.4	27.6	1.3	35
Pectoral-spine length	38.4	34.2	32.1	38.4	1.6	44	35.1	33.3	32.1	35.7	1.0	35
Pelvic-anal origin length	24.9	24.5	22.2	26.6	1.0	44	25.3	24.1	22.5	26.3	0.8	35
Pelvic-spine length	24.1	24.4	22.6	26.4	0.9	44	22.6	23.1	20.7	24.6	0.9	35
Postanal length	25.0	25.7	23.1	28.0	1.3	44	26.9	26.4	24.5	28.0	0.7	35
Anal-fin spine length	11.0	11.4	8.6	14.8	1.1	44	11.0	9.6	8.4	11.0	0.7	34
Dorsal spine length	23.2	24.6	22.8	26.7	1.0	44	23.7	24.1	21.6	25.8	0.9	34
Dorsal-fin base length	27.7	27.7	25.5	30.2	1.2	44	27.4	26.2	24.9	27.5	0.6	35
Dorsal-adipose distance	10.9	11.4	8.9	13.2	1.0	44	12.1	11.7	9.3	13.6	0.9	35
Caudal peduncle depth	11.3	11.0	9.8	12.2	0.5	44	10.9	10.6	10.0	11.0	0.3	35
Adipose-spine length	5.9	7.4	5.9	9.0	0.8	43	7.1	7.7	6.2	9.1	0.6	34
Adipose-Caudal length	20.7	21.0	17.8	23.9	1.2	44	20.7	21.4	19.8	23.5	0.8	35
Body depth at dorsal-fin origin	14.1	14.9	13.0	16.8	1.1	44	16.6	14.8	12.8	17.0	1.3	35
Body Width at dorsal-fin origin	30.2	28.0	25.7	31.2	1.2	44	29.4	29.2	27.6	30.7	0.9	35
Body Width at anal-fin origin	18.8	17.6	14.7	19.6	1.1	44	19.6	17.8	15.4	23.7	1.4	35
Postdorsal length	30.5	31.1	29.2	33.7	1.1	44	31.9	32.2	30.8	33.5	0.8	35
Anus-anal fin length	6.3	6.7	4.8	9.7	0.9	44	7.4	6.2	5.1	7.4	0.5	35
**% of Head length**												
Orbital diameter	14.3	16.5	13.8	20.2	1.5	44	15.5	17.0	15.5	19.4	1.0	35
Snout length	61.1	58.7	56.0	61.1	1.2	44	60.3	59.6	57.4	62.5	1.3	35
Internares width	15.8	13.8	9.8	16.8	1.3	44	15.1	14.3	12.6	17.4	1.0	35
Interorbital width	38.8	36.2	32.3	40.0	2.0	44	40.6	37.8	35.1	40.6	1.6	35
Head depth	37.4	39.9	36.8	45.3	2.0	44	42.6	39.8	36.3	43.1	1.7	35
Dentary tooth row length	17.6	17.4	15.0	19.6	1.1	44	17.7	18.3	16.7	20.2	0.8	35
Premaxillary tooth row length	18.0	18.5	16.5	22.7	1.2	44	17.2	19.0	16.7	20.4	0.9	35
Head width	96.7	97.6	90.3	105.3	3.3	44	104.2	100.7	92.8	108.4	3.7	35
Eye-nare length	12.9	12.1	10.4	13.8	0.7	44	13.8	13.0	11.2	14.5	0.8	35
Interbranchial distance	52.4	52.6	48.5	57.0	2.1	44	49.8	55.1	49.8	58.9	1.9	35
Lower lip width	58.9	60.0	54.6	64.7	2.9	41	57.6	58.3	52.8	64.8	2.8	35
Lower lip length	16.1	14.5	11.3	20.0	1.7	41	13.1	14.0	10.6	15.9	1.2	35
**Counts**		**Mode**						**Mode**				
Premaxillary teeth	61	51	42	69		44	52	58	45	72		35
Dentary teeth	64	61	37	67		44	54	53	45	78		35
Total lateral median plates	24	24	23	24		44	24	24	23	24		35
Plates between anal and caudal fins	9	9	8	10		44	9	9	8	9		35
Plates between dorsal and adipose fins	4	4	3	5		43	4	4	3	5		35
Predorsal plates	4	4	3	4		44	3	3	3	4		35

H = holotype, SD = standard deviation, n = number of measured specimens.

Body anterior region roughly trapezoidal, depressed, and slightly oval at caudal peduncle in cross section. Weak ridge from snout tip to anterior nare and from posterior nare to eye. Snout completely covered by plates except for small area on its tip. Lateral border of snout covered by strong, short and stiff odontodes. First four plates of mid-ventral series gently keeled. Ventral surface of caudal peduncle flat; small and delicate keel at three or four last ventral plates series of caudal peduncle. Remaining of body plates without keels.

Head large and wide. Eye rounded and moderate in size, dorsolaterally positioned at midpoint of head length; iris operculum present. Orbit not elevated; interorbital area flat or nearly straight. Parieto-supraoccipital almost indistinct from rest of the skull bones. Supraoccipital process not elevated, slightly rounded posteriorly. Parieto-supraoccipital limited posteriorly by four roughly triangular plates. Predorsal area with three pairs of predorsal plates; plates of third pair connected anteriorly but separated posteriorly by small nuchal plate.

Mouth and lips of moderate size; oral disk elliptical; lips almost completely covered with small round papillae densely packed, slightly larger on central portion of lower lip; lips area surrounding premaxillary and dentary smooth, without papillae. Lower lip large, but not reaching pectoral girdle. Maxillary barbel short and with small portion free from lower lip. Teeth short, thin, delicate, and bicuspid. Mesial cusp slightly larger and wider than lateral cusp; lateral cusp reaching two-thirds of mesial cusp length. Premaxilla and dentary of similar size and disposed parallel to anterior border of snout. Single and reduced buccal papilla between premaxilla.

Dorsal body surface completely covered by large plates, except immediately around dorsal-fin base. Ventral surface entirely devoid of plates from snout to anal-fin origin. Base of first anal-fin pterygiophore covered by skin, preanal plate absent. Caudal peduncle completely covered by plates. Median series plates 24. Five series of lateral plates at caudal peduncle. Six to seven oblong plates on caudal-fin base. Opercle exposed sickle-shaped and covered by odontodes; plates immediately posterior to opercle of moderate size, sometimes reaching half of opercle length. Plates of supraopercular area of moderate size and few in number, leaving large naked area around opercle. Three large, strong, and curved odontodes on cheek plates, reaching the posterior margin of branchial opening; fleshy and thick sheath sometimes covering two-thirds of cheek odontodes. All plates covered with small and aligned odontodes lines; odontodes slightly larger on opercle, postopercular plate and pectoral-fin spine.

Dorsal-fin origin on anterior portion of body, slightly anterior to vertical through pelvic-fin origin. Dorsal fin II,7; spinelet present and very reduced, dorsal-fin locking mechanism not functional. Dorsal fin short and low, reaching adipose-fin when adpressed. Four to five plates separating dorsal from adipose fin. Adipose fin short and low. Two unpaired dorsal plate between adipose fin and first procurrent caudal-fin ray. Caudal fin i,14,i, slightly emarginated, with oblique distal margin, and ventral lobe slightly longer than dorsal. Pectoral fin I,6, very large, reaching urogenital opening when adpressed. Pectoral-fin spine thick and strong, not pungent, covered by large odontodes. Pelvic fin i,5 reaching end of anal-fin base when adpressed. Anal fin i,5, moderated. All rays covered by numerous short odontodes on their free surface. Anteriormost branched rays of pelvic and anal fins larger than unbranched rays. Four to five dorsal and ventral procurrent caudal-fin rays. Vertebrae counts 28 (4). Seven (3) or eight (1) rib pairs.

#### Color in alcohol

Background color light to dark gray on head, trunk and fins. Head, trunk, and dorsal, caudal, and anal fins, and dorsal surface of pectoral and pelvic fins covered by small whitish-yellow dots, smaller than pupil. Caudal fin distal band yellowish-white when visible. Ventral surface cream-colored or light brown (Fig 10).

#### Color in life

Background color dark olive to black on head, dorsum and sides, fins slightly clearer than body; ventral surface of pectoral and pelvic fins light gray or yellowish-white. Small greenish-yellow dots covering body and fins. Caudal fin with yellow distal band, wider in juveniles and narrowing along ontogenetic development. In adults, caudal fin band, when present, becoming reduced to blotch on distal portion of both lobes. Ventral surface light gray to yellowish-white without dots; some individuals with dots on lateral portion of abdomen and around anus and anal fin. Considerable variation occurs in density, size and color intensity of dots (Figs 5C, 11 and 12).

#### Distribution

*Hopliancistrus wolverine* is currently known from the middle Rio Xingu, from immediately downriver the Belo Monte Village, throughout the Volta Grande do Xingu to the vicinity of São Félix do Xingu, and in the Rio Iriri, immediately upstream its confluence to Xingu until the mouth of the Rio Curuá (Fig 8).

#### Etymology

The specific name is an allusion to the Mustelidae *Gulo gulo*, also known as wolverine, glutton, carcajou, skunk bear, or quickhatch because of it's blunt stature, strong claws, and ferocity. Name in apposition.

#### Remarks

This species is referred to in some aquarium fish publications as L017 and LDA15 [16], but the code L017 was coined to depict a *Pseudancistrus* (Stawikowski [20]: 174 [Fig 9]) today known as *P*. *asurini*. One individual of the lot INPA 40877 shows a hypertrophied membrane posterior to last ray of dorsal-fin, covering four plates immediately behind it, but not reaching the preadipose plate, thus a similar but distinct condition than what is observed in species of *Baryancistrus* Rapp Py-Daniel, 1989.

### *Hopliancistrus xikrin*, new species

urn:lsid:zoobank.org:act:FD6CCF04-1BA5-4F7A-A570-7B6B3157FCAD

(Figs 5D and 13)

*Hopliancistrus* sp. L171. −Stawikowski [23]: 533 [DATZ magazine, new import from Xingu/Iriri]. −Seidel & Evers [14]: 329 [Aquarium fish Atlas].
*Hopliancistrus* sp. 2 (L171). −Camargo et al. [15]: 159 (picture of *Hopliancistrus tricornis* erroneously used as L171, same picture from Seidel & Evers [14]: 323).

**Fig 13:**
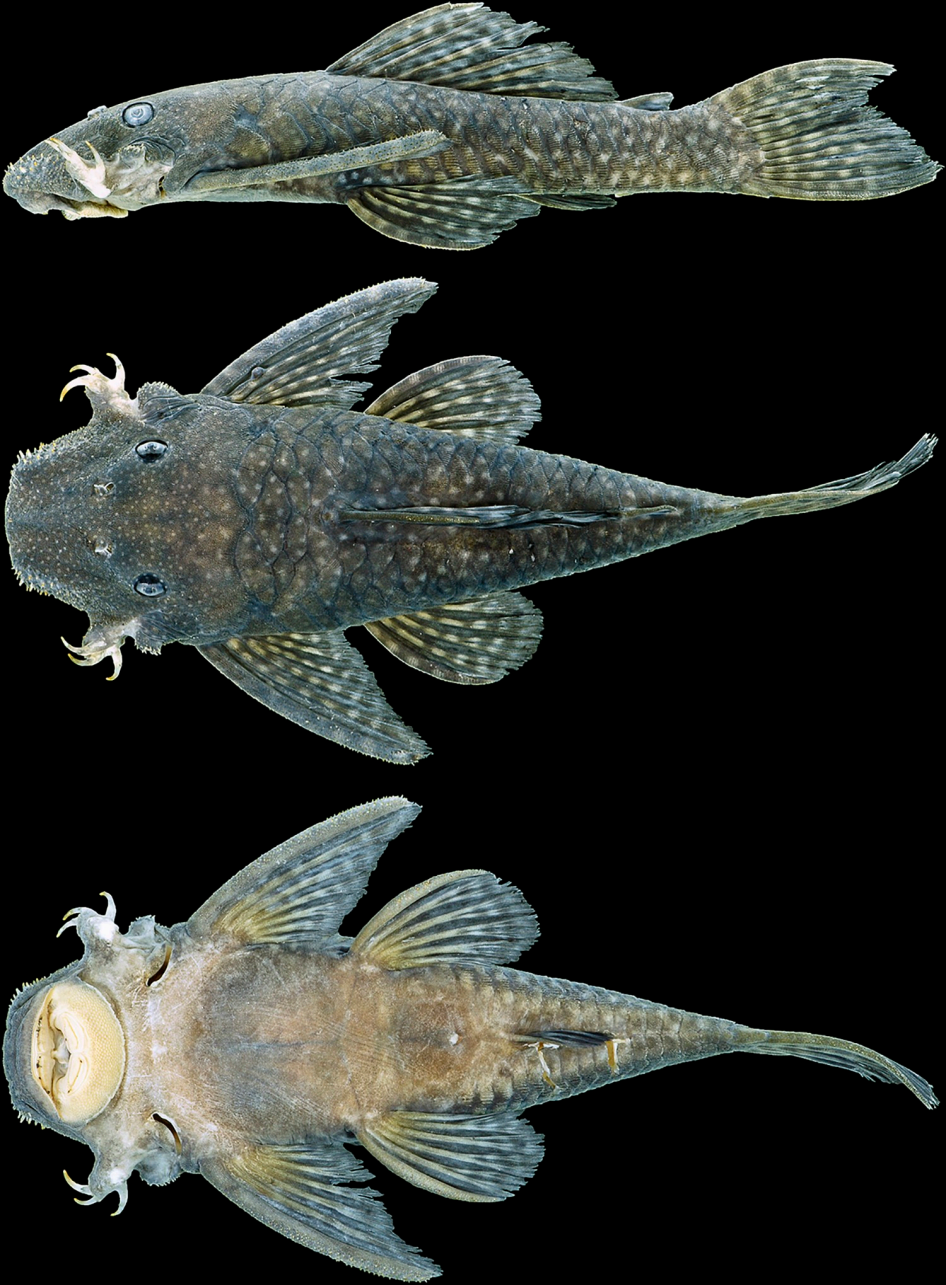
Holotype of *Hopliancistrus xikrin*. INPA 37903, 131.4 mm SL, from Rio Bacajá, Rio Xingu basin.

#### Holotype

INPA 37903, 131.3 mm SL, Brazil, Pará, Altamira, Rio Bacajá, ca. 2 km upstream from confluence with Rio Xingu, 3°31’10”S 51°42’35”W, Rio Xingu basin, 15 Oct 2012, M. Sabaj-Pérez, L. M. Sousa & M. Arce.

#### Paratypes

All from Brazil, Pará, Rio Xingu basin: ANSP 193087, 3, 70.0–92.6 mm SL; INPA 38096, 2, 65.7–69.2 mm SL, Rio Bacajá, ca. 2 km upstream from confluence with Rio Xingu, 3°31’10”S 51°42’35”W, 15 Oct 2012, M. Sabaj-Pérez, L. M. Sousa & M. Arce. INPA 35302, 1, 94.6 mm SL, Altamira, Rio Bacajá, 3°34’39”S 51°35’38”W, 28 Oct 2010, T. Giarrizzo, M. C. Andrade & D. Bastos. INPA 35742, 1, 106.6 mm SL, Altamira, Rio Bacajá, Cachoeira Alpercata, 3°31’04”S 51°45’00”W, 27 Sep 1996, J. Zuanon. INPA 37355, 1, 126.2 mm SL, Altamira, Rio Bacajá, 3°32’44”S 51°41’03”W, 25 Jul 2011, M. C. Andrade. INPA 37356, 1, 141.6 mm SL, Altamira, Rio Bacajá, 3°40’55”S 51°32’43”W, 22 Jul 2011, M. C. Andrade. INPA 40268, 1, 95.8 mm SL, Anapu, Rio Bacajá, first large rapid, ca. 1.7 km upstream of confluence with Rio Xingu, 3°31’10”S 51°42’35”W, 15 Sep 2013, M. Sabaj-Pérez, L. M. Sousa, A. S. Oliveira & P. M. Ito. INPA 40902, 11, 43.7–128.2 mm SL, Vitória do Xingu, Rio Bacajá, Pariaxá, 3°34’28”S 51°39’22”W, 22 Sep 2012, D. A. Bastos & J. Santos. INPA 40903, 14 alc, 36.1–120.0 mm SL and 1 cs, 98.5 mm SL, Vitória do Xingu, Rio Bacajá, Seca Farinha, 3°43’38”S 51°33’59”W, 23 Sep 2012, D. A. Bastos & J. Santos. INPA 40904, 7, alc, 62.6–110.3 mm SL and 1 skel, 133.8 mm SL, Vitória do Xingu, Rio Bacajá, Seca Farinha, 3°45’45”S 51°35’03”W, 10 Jul 2012, L. M. Sousa & R Fróis. INPA 40905, 9, 36.0–99.4 mm SL, Vitória do Xingu, Rio Bacajá, Seca Farinha, 3°45’45”S 51°35’03”W, 9 Jul 2012, L. M. Sousa & R. Fróis. INPA 40906, 2, 62.9–66.9 mm SL, Vitória do Xingu, Rio Bacajá, Pariaxá, 3°34’37”S 51°35’36”W, 24 Sep 2012, D. A. Bastos & J. Santos. INPA 52331, 8, 65.9–141.5 mm SL, Vitória do Xingu, Rio Bacajá, 3°31’26”S 51°42’17”W, 3 Sep 2012, E. D. Ribeiro & L. Sousa. LIA 679, 19, 25.3–84.4 mm SL, Senador José Porfírio, Igarapé Bacajaí, 3°38’41.7”S 51°45’13.8”W, 12 Jul 2014, A. Gonçalves, D. Bastos and fishermen Neto, Toninho and Tita. LIA 2493, 5, 64.1–88.0 mm SL, São Félix do Xingu, Pedra Preta, Rio Xingu, 6°57’31.6”S 52°17’12.8”W, 23 May 2015, A. Gonçalves, D. Silva and fishermen Ney, Toninho, Sergio and Fábio. LIA 2819, 10, 27.5–78.6 mm SL, São Félix do Xingu, Triunfo stream, nearl 5 km above of mouth, 6°22’21.8”S 52°29’5.7”W, 31 May 2015, A. Gonçalves, D. Silva and fishermen Ney, Toninho, Sergio and Fábio. LIA 2905, 16, 33.8–102.1 mm SL, Senador José Porfírio, waterfall of Rio Bacajá, 3°38’41.7”S 51°45’13.8”W, 8 Jun 2015, A. Gonçalves, D. Silva and fishermen Ney, Toninho, Sergio and Fábio. LIA 2933, 16, 44.7–125.3 mm SL, Senador José Porfírio, waterfall of Rio Bacajá, 2,3 km below of camp 10, 3°5’437.9”S 51°44’32.7”W, 9 Jun 2015, A. Gonçalves, D. Silva and fishermen Ney, Toninho, Sergio and Fábio. LIA 2951, 8, 26.2–80.9 mm SL, Senador José Porfírio, rocks, Rio Bacajá, nearly 600 m below of camp 10, 3°53’55.3”S 51°43’28.1”W, 9 Jun 2015, A. Gonçalves, D. Silva and fishermen Ney, Toninho, Sergio and Fábio. LIA 2956, 7, 32.3–91.6 mm SL, Senador José Porfírio, Rocks, Rio Bacajá, nearly 400 m below of camp 10, 3°54’28.3”S 51°44’4.0”W, 9 Jun 2015, A. Gonçalves, D. Silva and fishermen Ney, Toninho, Sergio and Fábio. LIA 2963, 7, 31.1–99.5 mm SL, Senador José Porfírio, rock, Rio Bacajá, nearly 6 km below of camp 11, 3°41’55.8”S 51°44’23.0”W, 11 Jun 2015, A. Gonçalves, D. Silva and fishermen Ney, Toninho, Sergio and Fábio. LIA 2971, 3, 36.6–61.3 mm SL, Senador José Porfírio, Rio Bacajá, nearly 45 km below of camp 11, 3°39’41.9”S 51°45’38.5”W, 11 Jun 2015, A. Gonçalves, D. Silva and fishermen Ney, Toninho, Sergio and Fábio. LIA 2977, 5, 43.7–66.6 mm SL, Senador José Porfírio, rocks, Rio Bacajá, nearly 1.5 km below of camp 11, 3°41’28.9”S 51°45’0.5”W, 11 Jun 2015, A. Gonçalves, D. Silva and fishermen Ney, Toninho, Sergio and Fábio. MCP 54541, 1, 105.5 mm SL, Altamira, Morro do Félix/RESEX do Rio Xingu, 4°32’47.6”S 53°40’21.5”W, 17 Feb 2016, A. Gonçalves, D. Silva and fishermen Ronca and Fábio. MNRJ 35605, 13, 12 alc., 34.6–75.4 mm SL and 1 cs, 64.2 mm SL, Ourilândia, Igarapé tributary of left margin of the Rio Jauri (sub-drainage Rio Fresco), ca. of 1 km east of Rio Jauri on road, 7°13’24”S 50°37’10”W, 10 Oct 2008, P. A. Buckup, J. Birindelli & C. Chamon. MNRJ 35606, 3, 54.4–64.2 mm SL, Ourilândia do Norte, Rio Jauri (sub-drainage Rio Fresco), road Bannach-Ourilândia do Norte, 7°13’14”S 50°37’26”W, 10 Oct 2008, P. A. Buckup, J. Birindelli & C. Chamon. MPEG 38948, 5, 62.0–93.4 mm SL, São Félix do Xingu, Igarapé Triunfo, nearly 5 km above of mouth, 6°22’24.3”S 52°29’6.2”W, 1 Jun 2015, A. Gonçalves, D. Silva and fishermen Ney, Toninho, Sergio and Fábio. MZUEL 20813, 3, 70.6–81.8 mm SL, São Félix do Xingu, Igarapé Triunfo, nearly 4 km above of mouth, 6°21’38.6”S 52°29’5.7”W, 31 May 2015, A. Gonçalves, D. Silva and fishermen Ney, Toninho, Sergio and Fábio. NUP 22723, 4, 78.5–115.9 mm SL, Altamira, Rio Novo, between RESEX do Rio Iriri and Estação Ecológica da Terra do Meio, 4°32’47.6”S 53°40’21.5”W, 1 Feb 2016, A. Gonçalves, D. Silva and fishermen Ney, Toninho, Sergio and Fábio. ZUEC 17327, 4, 30.8–95.4 mm SL, Senador José Porfírio, Igarapé Bacajaí, 3°45’30.5”S 51°42’7.4”W, 11 Jul 2014, A. Gonçalves, D. Bastos and fishermen Neto, Toninho and Tita.

#### Diagnosis

*Hopliancistrus xikrin* is distinguished from its congeners by having ground body color brown with fainted whitish-yellow spots of moderate size (usually smaller than pupil) on posterior portion of body (vs. body color black or dark olive covered by conspicuous greenish-yellow smalls dots of similar size on head, trunk and fins in *H*. *wolverine*; body color brown with large fainted whitish-yellow spots on posterior portion of the body, spots always larger than pupil in *H*. *tricornis*, *H*. *munduruku* and *H*. *xavante*). *Hopliancistrus xikrin* can be further distinguished from its congeners by the absence of contact between the transverse processes of first and second dorsal-fin pterygiophores (vs. the transverse processes of first and second dorsal-fin pterygiophores sutured to each other). *Hopliancistrus xikrin* is distinguished from its congeners except *H*. *wolverine* by pectoral-fin spine length 32.1–35.7% of SL (vs. 25.1–29.9% in *H*. *tricornis*, 24.5–30.9% in *H*. *munduruku* and 27.4–30.8% of SL in *H*. *xavante*). *Hopliancistrus xikrin* also differs from *H*. *tricornis* and *H*. *wolverine* by the connection strut between anteroventral process of compound pterotic and the main body shaped as a continuous sheet (vs. connection strut narrow and bar-shaped, leaving a large posterior gap, see Fig 6). *Hopliancistrus xikrin* differs from *H*. *xavante* by having dorsal, caudal, and anal fins covered by dark brown to black spots (vs. all fins covered by large yellowish-white spots); by narrow nasal bone plate (vs. nasal broad, sometimes slightly triangular, see Fig 2); by having nuchal plate exposed, and covered by odontodes (vs. nuchal plate covered by thick skin and usually lacking odontodes, see Fig 7); and by caudal peduncle depth 10.0–11.0% of SL (vs. 11.5–12.9% of SL). *Hopliancistrus xikrin* also differs from *H*. *tricornis* by the possession of five branched rays on anal-fin (vs. four).

#### Description

Body shape and pigmentation in Figs 5D and 13. Morphometric data and counts in Table 2. Small-sized loricariid, with largest examined specimen 141.6 mm SL. Head roughly square anteriorly in dorsal view; body gradually narrowing from cleithrum to caudal-fin origin. Greatest width of body at cleithrum. Head and trunk depressed, greatest depth at dorsal-fin origin. In lateral view, head dorsal profile from snout tip to eyes sloped, flat from eyes to dorsal-fin origin and nearly straight or slightly declining to first procurrent ray of caudal-fin. Ventral profile straight from snout tip to caudal-fin origin.

Anteriorly, body roughly trapezoidal, depressed, and slightly oval at caudal peduncle in cross section. Weak ridge from snout tip to anterior nare, and from posterior nare to eye. Snout completely covered by small plates except for small naked area on tip. Lateral border of snout covered by short and stiff odontodes. First four plates of mid-ventral series gently keeled. Ventral surface of caudal peduncle flat; small and delicate keel at three or four last ventral plates series of caudal peduncle. Remaining of body plates without keels.

Head large and wide. Eye rounded and moderate in size, dorsolaterally positioned at midpoint of head length; iris operculum present. Orbit not elevated; interorbital area nearly straight. Parieto-supraoccipital almost indistinct from other skull bones. Supraoccipital process not elevated, slightly rounded posteriorly. Parieto-supraoccipital limited posteriorly by four roughly triangular plates. Predorsal area reduced, with three pairs of predorsal plates, plus one small plate anterior to dorsal-fin spinelet. Nuchal plate situated between plates of third predorsal pair.

Mouth and lips of moderate size; oral disk elliptical; lips almost completely covered with small round papillae densely packed, slightly larger on central portion of lower lip; area around premaxillary and dentary smooth, without papillae. Lower lip large, but not reaching pectoral girdle. Maxillary barbel short with small portion free from lower lip. Teeth short, thin, delicate, and bicuspid. Mesial cusp slightly larger and wider than lateral cusp; lateral cusp reaching two-thirds of mesial cusp length. Premaxilla and dentary of similar size and disposed parallel to anterior border of snout. Single and reduced buccal papilla between premaxilla.

Dorsal body surface completely covered by large plates, except immediately around dorsal-fin base. Ventral surface entirely devoid of plates from snout tip to anal-fin origin. Base of first anal-fin pterygiophore covered by skin, preanal plate element absent. Caudal peduncle completely covered by plates. Median plates series 23–24. Five series of lateral plates at caudal peduncle. Five to six oblong plates on caudal-fin base. Opercle exposed sickle-shaped and covered by odontodes; plates immediately posterior to opercle small, sometimes reaching half of length of exposed opercle. Plates of supraopercular area moderate-sized and few in number, leaving large naked area around opercle. Three large, strong, and curved odontodes on cheek plates, reaching posterior margin of branchial opening; fleshy and thick odontodes sheath covering two-thirds of odontodes. All plates covered with aligned small-sized odontodes; odontodes slightly larger on opercle, postopercular plate and on pectoral-fin spine.

Dorsal-fin origin on anterior portion of body, slightly anterior to vertical through pelvic-fin origin. Dorsal fin II,7; spinelet present and very reduced, dorsal-fin locking mechanism not functional. Dorsal fin large and low, reaching preadipose plate when adpressed. Four to five plates separating dorsal from adipose fins. Adipose fin short and low. Two unpaired dorsal plates between adipose fin and first procurrent caudal-fin ray usually present. Caudal fin i,14,i, slightly emarginated, with oblique distal margin, and ventral lobe slightly longer. Pectoral fin I,6, large, reaching urogenital opening when adpressed. Pectoral-fin spine thick and strong, not pungent, covered by large odontodes. Pelvic fin i,5 reaching end of anal-fin base when adpressed. Anal fin i,5, moderate-sized. All rays covered by numerous short odontodes on their free surface. Anteriormost branched rays of pelvic and anal fins larger than unbranched rays. Four to six dorsal procurrent caudal-fin rays and four to five ventral procurrent caudal-fin rays. Vertebral counts: 27 (1) to 28 (2). Seven (1), eight (1) or nine (1) rib pairs.

#### Color in alcohol

Body uniformly dark brown with inconspicuous mottled clear blotches. All fins uniformly dark; some specimens with clear spots larger than pupil on fins; dorsal fin with irregular dark spots on rays. Ventral surface from snout tip to border of upper lip dark gray; lower lip cream, whitish-yellow from lower lip to pectoral girdle, then light gray to caudal fin origin (Fig 13).

#### Color in life

Body ground color light to dark brown at head, dorsum and flanks. Head covered by very small inconspicuous white dots; trunk and flanks covered with inconspicuous whitish spots, smaller than pupil size. Dorsal fin smudged with light brown to dark spots over rays. Pectoral, pelvic, anal, and caudal fins gray, covered by white spots on caudal and anal fins and dorsal surface of pectoral and pelvic fins. Caudal fin with little yellow blotch on distal portion of both caudal fin lobes in juveniles, absent in adults. Ventral surface from snout tip to border of upper lip dark gray, whitish-yellow from lower lip to pectoral girdle, then gray to caudal fin origin (Fig 5D).

#### Distribution

*Hopliancistrus xikrin* is known from small to medium-sized tributaries of the middle portion of Rio Xingu, like Rio Bacajá and Rio Bacajaí, at the Volta Grande area (Fig 8).

#### Etymology

The new species is named after the Xikrin, an indigenous ethnic group inhabiting the Rio Bacajá margins. The Xikrin people belong to the "Je" linguistic trunk, calling themselves "Mebengokre". They are related to the Kayapo people with whom they share the same self-denomination, cultural characteristics such as body painting patterns, language, residence and marital relations, rituals, chants, and naming system.

### *Hopliancistrus xavante*, new species

**Fig 14:**
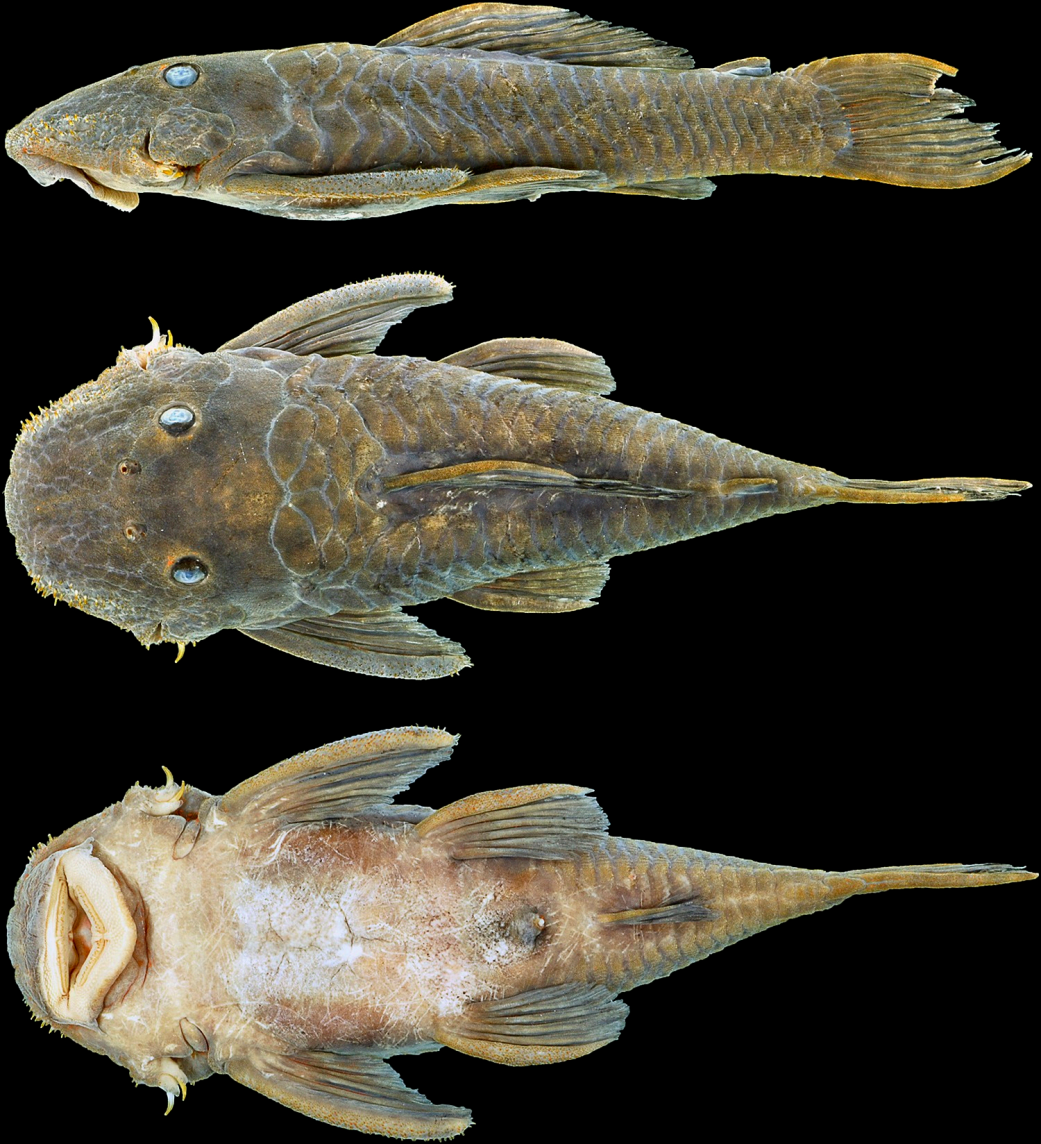
Holotype of *Hopliancistrus xavante*. INPA 35273, 114.2 mm SL, from Rio Culuene, upper Rio Xingu basin.

**Fig 15:**
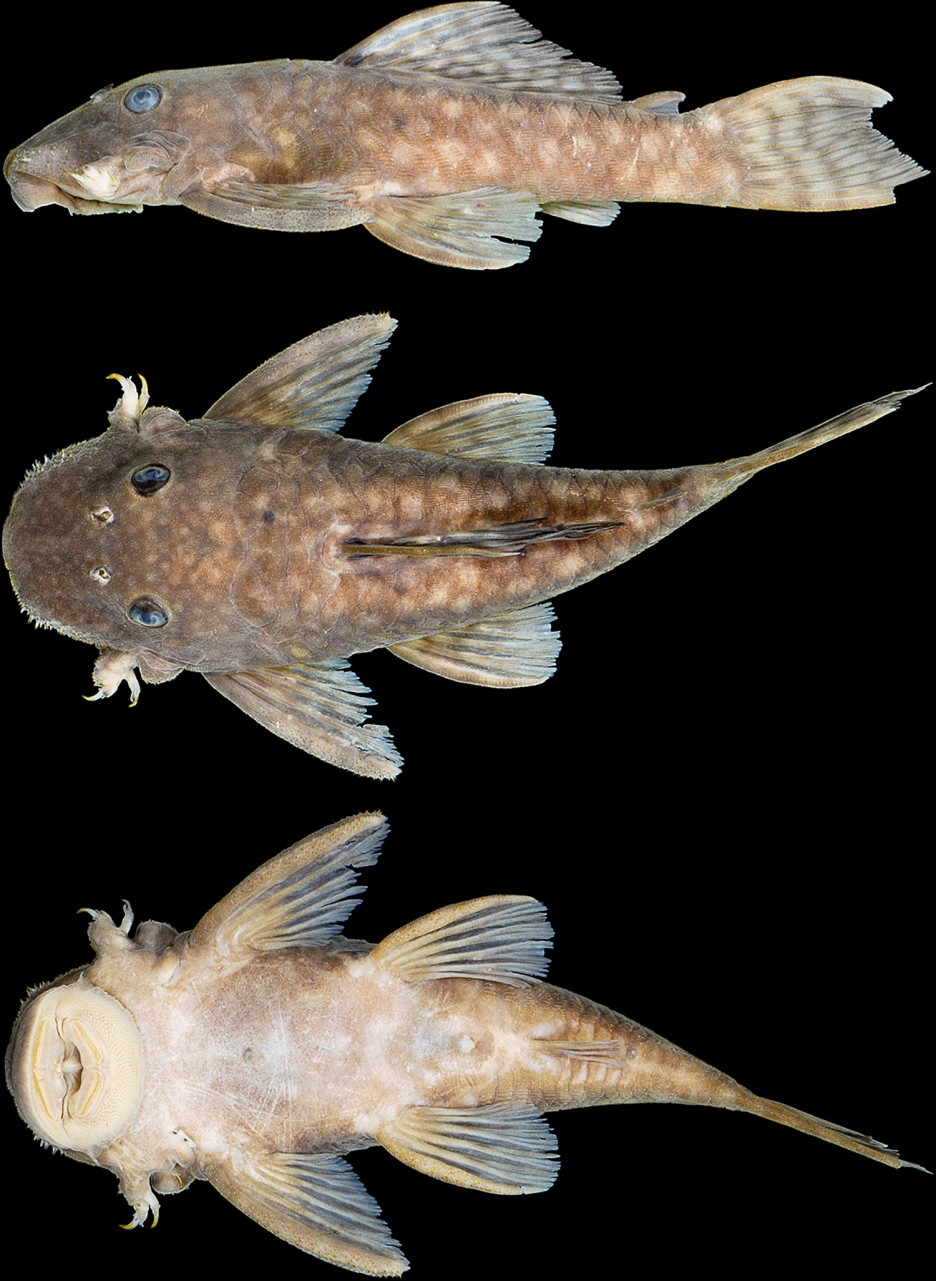
Paratype of *Hopliancistrus xavante*. MZUSP 89805, 84.8 mm SL, from Rio Culuene, tributary of Rio Xingu.

urn:lsid:zoobank.org:act:2FFD3600-A6D3-48B4-A6C5-7E6DDB829529

(Figs 5E, 14 and 15)

*Hopliancistrus tricornis*.–Isbrücker & Nijssen [4]: 544 [in part, two lots mixed in the type series of *Hopliancistrus tricornis*].
*Hopliancistrus* sp. "Rio da Paz" (L422).–Stawikowski [24]: 35 [DATZ magazine. Fish from Rio da Paz, a small eastern affluent of the Rio Xingu a few kilometers north of the border between the Brazilian states of Pará and Mato Grosso.]

#### Holotype

INPA 35273, 114.2 mm SL, Brazil, Mato Grosso, Paranatinga, Rio Culuene, Small Hidroelectric powerplant Paranatinga 2 coffeudam, 13°49’00”S 53°15’00”W, Xingu River basin, 2 Jul 2007, L. M. Sousa et al.

#### Paratypes

All from Brazil, Mato Grosso, Rio Xingu basin: INPA 37616, 33 alc, 32.3–116.5 mm SL, 2 skel, 103.3–114.7 mm SL and 4 cs, 54.4–85.3 mm SL, collected with holotype. MZUSP 87061, 2, 60.7–74.5 mm SL, Gaúcha do Norte, Rio Curisevo, beach on road to Sorriso, ca. of 30 km W of Gaúcha do Norte, 13°12’58”S 53°29’53”W, 19 Oct 2004, C. Moreira et al. MZUSP 89805, 2, 76.2–84.8 mm SL, Paranatinga, Rio Sucuri, tributary right margin of the Rio Culuene, near its mouth, 13°55’40”S 53°17’10”W, 15–22 Jan 2006, A. Akama & J. Birindelli. MZUSP 94134, 4, 59.2–89.2 mm SL, Gaúcha do Norte, Rio Culuene, farm of Zezé (2 km above bridge), 13°30’53”S 53°05’40”W, 21–26 May 2007, F.C.T. Lima et al. MZUSP 94189, 2, 90.4–99.2 mm SL, Campinápolis, Rio Culuene, rapids (area of future Small Hidroelectric powerplant Paranatinga 2) up until 1 km downstream, approx. 13°49’S 53°15’W, 19–20 May 2007, F.C.T. Lima et al. MZUSP 98180, 34, 34.0–107.5 mm SL (4 measured), Campinápolis, Rio Culuene, Cachoeira de Adelino (= Cachoeira do Culuene), 13°47’50”S 53°14’46”W, 2–14 Oct 2007, F.C.T. Lima et al.

#### Non-types

MZUSP 94848, 315, 20.5–115.8 mm SL, Mato Grosso, Paranatinga, Rio Culuene, Small Hidroelectric powerplant Paranatinga 2 coffeudam, approx. 13°49’S 53°15’W, Rio Xingu basin, 2 Jul 2007, L. M. Sousa et al. MZUSP 91975, 1, 106.5 mm SL, Mato Grosso, Paranatinga, Rio Culuene, on future area of Small Hidroelectric powerplant Paranatinga 2, approx. 13°49’S 53°15’W, Rio Xingu basin, 21 Aug 2006, J. L. Birindelli et al. IRSNB 692, 20; ZMA 120.339, 19, 50.0–70.0 mm SL, Brazil, Mato Grosso, Cachoeira von Martius, Station 114, upper Xingu, 29 Oct 1964, J. P. Gosse.

#### Diagnosis

*Hopliancistrus xavante* can be distinguished from its congeners by the nuchal plate covered by thick skin (Fig 7), and usually lacking odontodes (vs. nuchal plate exposed and covered by odontodes); by having a broad nasal bone plate (Fig 2), sometimes slightly triangular (vs. narrow nasal); and by the trunk and fins covered by large yellowish-white spots (vs. dorsal, caudal, and anal fins covered by dark brown to black spots in *H*. *tricornis*, *H*. *munduruku*, and *H*. *xikrin*; body covered by conspicuous small greenish-yellow dots of similar size on head, trunk, and fins in *H*. *wolverine*). *Hopliancistrus xavante* can be further distinguished from its congeners by a combination of additional characters: it differs from its congeners except *H*. *wolverine* by caudal peduncle depth 11.5–12.9% of SL (vs. 9.7–11.3% in *H*. *tricornis*, 10.1–11.3% in *H*. *munduruku*, 10.0–11.0% in *H*. *xikrin*). *Hopliancistrus xavante* differs from *H*. *tricornis* and *H*. *wolverine* by connection strut between anterior process of compound pterotic and main body shaped as a continuous sheet (vs. connection strut narrow and bar-shaped, leaving a large posterior gap, see Fig 6). It differs from *Hopliancistrus xikrin* by the transverse processes of first and second dorsal-fin pterygiophores sutured to each other (vs. absence of contact between the transverse processes of first and second dorsal-fin pterygiophores). *Hopliancistrus xavante* also differs from *H*. *tricornis* by the possession of five branched rays on anal-fin (vs. four).

#### Description

Body shape and pigmentation in Figs 5E, 14 and 15. Morphometric data and counts in Table 3. Small size loricariid, with largest examined specimen reaching 116.5 mm of SL. Head roughly square anteriorly in dorsal view; from cleithrum to dorsal insertion line straight, then gradually narrowing to caudal-fin origin. Greatest width of body at head. Overall body corpulent, strong. Head and trunk wide and moderately depressed, greatest depth at dorsal-fin origin. In lateral view, dorsal profile sloped and slightly convex from snout tip to eye, then plane with low decline to first procurrent caudal-fin ray. Ventral profile straight from snout tip to caudal-fin origin.

**Table 3:** Morphometric data and counts of *Hopliancistrus xavante* from upper Rio Xingu basin.

	*Hopliancistrus xavante*					
Measurement	H	Mean	Min	Max	SD	N
Standard length	114.2		60.7	116.5		28
**% of standard length**						
Predorsal length	43.6	45.0	42.9	46.4	0.9	28
Head length	35.8	36.2	35.1	37.4	0.6	28
Cleithral width	34.0	34.5	33.6	35.6	0.5	28
Cleithral process width	31.9	32.2	31.1	34.0	0.7	28
Pectoral-Pelvic origin length	26.3	27.2	25.3	29.4	1.2	28
Pectoral-spine length	29.5	29.3	27.4	30.8	1.0	28
Pelvic-anal origin length	24.6	23.9	22.4	25.8	0.9	28
Pelvic-spine length	23.0	22.9	21.1	25.0	0.9	28
Postanal length	23.5	24.0	23.1	25.4	0.6	28
Anal-fin spine length	12.1	11.3	8.6	12.7	1.0	27
Dorsal spine length	24.6	23.2	21.1	25.2	1.2	27
Dorsal-fin base length	28.1	27.3	26.1	28.4	0.6	28
Dorsal-adipose distance	12.2	12.5	10.5	14.1	1.0	28
Caudal peduncle depth	12.0	12.0	11.5	12.9	0.3	28
Adipose-spine length	7.0	6.6	4.7	7.9	0.8	27
Adipose-Caudal length	20.2	20.9	19.4	22.9	0.9	28
Body depth at dorsal-fin origin	19.9	17.8	15.2	20.1	1.3	28
Body Width at dorsal-fin origin	30.8	29.4	27.4	31.5	1.0	28
Body Width at anal-fin origin	19.9	18.9	17.6	20.6	0.7	28
Postdorsal length	29.8	31.5	28.7	33.2	1.0	28
Anus-anal fin length	6.3	6.9	5.9	8.4	0.6	28
**% of Head length**						
Orbital diameter	15.5	16.6	15.0	18.6	1.0	28
Snout length	63.4	61.7	58.3	63.8	1.6	28
Internares width	16.0	15.7	13.4	19.0	1.3	28
Interorbital width	38.8	36.9	34.2	42.0	1.7	28
Head depth	49.7	46.3	42.9	49.7	1.7	28
Dentary tooth row length	19.1	20.1	18.7	21.9	0.9	28
Premaxillary tooth row length	21.8	20.8	19.2	22.7	0.9	28
Head width	100.6	100.4	93.1	105.9	3.2	28
Eye-nare length	14.1	13.4	12.1	14.7	0.7	28
Interbranchial distance	57.5	56.4	54.2	58.3	1.1	28
Lower lip width	60.8	59.8	57.2	61.4	1.5	11
Lower lip length	16.5	15.5	13.8	17.5	1.0	11
**Counts**		**Mode**				
Premaxillary teeth	52	54	38	62		20
Dentary teeth	45	45	39	60		20
Total lateral median plates	25	24	24	25		20
Plates between anal and caudal fins	9	9	8	9		20
Plates between dorsal and adipose fins	4	4	4	5		20
Predorsal plates	3	3	3	4		22

H = holotype, SD = standard deviation, n = number of measured specimens.

Anteriorly, body roughly trapezoidal, depressed, and slightly oval at caudal peduncle in cross section. Weak ridge from snout tip to anterior nare and from posterior nare to eye. Snout completely covered by plates except for small naked area on tip. Lateral border of snout covered by strong, short and stiff odontodes. First four plates of mid-ventral series gently keeled. Ventral surface of caudal peduncle flat; small and delicate keel at three or four last ventral plates of caudal peduncle. Remaining of body plates without keels.

Head large and wide. Eye rounded and moderate in size, dorsolaterally positioned just posterior to midpoint of head length; iris operculum present. Orbit not elevated; interorbital area nearly straight. Parieto-supraoccipital almost indistinct from rest the other bones of the skull. Supraoccipital process not elevated, slightly rounded posteriorly. Parieto-supraoccipital limited posteriorly by four roughly triangular plates. Predorsal area with three pairs of predorsal plates, nuchal plate not exposed, covered by skin and usually lacking odontodes.

Mouth and lips of moderate size; oral disk elliptical; lips almost completely covered with small round papillae densely packed, slightly larger on central portion of lower lip; lip area surrounding premaxillary and dentary smooth, without papillae. Lower lip moderate in size, not reaching pectoral girdle. Maxillary barbel short and with small portion free from lower lip. Teeth long, thin, delicate, and bicuspid. Mesial cusp slightly larger and wider than lateral cusp; lateral cusp reaching two-thirds of mesial cusp length. Premaxilla and dentary of similar size and disposed parallel to anterior border of snout. Single and small buccal papilla between premaxilla.

Dorsal body surface completely covered by large plates, except immediately around dorsal-fin base. Ventral surface entirely devoid of plates from snout to anal-fin origin. Base of first anal-fin pterygiophore covered by skin, preanal plate absent. Caudal peduncle completely covered by plates. Median series plates 24–25. Five series of lateral plates at caudal peduncle. Six to seven oblong plates on caudal-fin base. Exposed opercle sickle-shaped and covered by odontodes; plates immediately posterior to opercle of moderate size, sometimes reaching half of opercle length. Plates of supraopercular area small and few in number, leaving large naked area around opercle. Three large, strong, and curved odontodes on cheek plates, reaching the posterior margin of branchial opening; fleshy and thick odontodes sheath covering two-thirds of cheek odontodes. All plates covered with small and aligned odontodes; odontodes slightly larger on opercle, postopercular plate and pectoral-fin spine.

Dorsal-fin origin on anterior portion of body, slightly anterior to vertical through pelvic-fin origin. Dorsal fin II,7; spinelet present but very reduced, dorsal-fin locking mechanism not functional. Dorsal fin short and low, not reaching adipose-fin spine when adpressed. Four to five plates separating dorsal from adipose fins. Adipose fin short and low, slightly more developed than congeners. Two unpaired dorsal plates between adipose fin and first procurrent caudal-fin ray usually present. Caudal fin i,14,i, slightly emarginated, with oblique distal margin, and ventral lobe slightly longer. Pectoral fin I,6, moderate in size, reaching end of pelvic-fin base when adpressed. Pectoral-fin spine thick and strong, not pungent, covered by large odontodes. Pelvic fin i,5 not reaching end of anal-fin base when adpressed. Anal fin i,5, moderated in size. All rays covered by numerous short odontodes on their free surface. Anteriormost branched rays of pelvic and anal fins slightly larger than unbranched rays. Four to six dorsal procurrent caudal-fin rays and four to five ventral procurrent caudal-fin rays. Vertebrae counts 28 (6). Eight (2) or nine (4) rib pairs.

#### Color in alcohol

Body ground color light brown at dorsum and sides. Head, dorsum, and sides covered by yellowish-white spots, sometimes absent, smaller than pupil diameter on head and larger than pupil on trunk. Dorsal, caudal, and anal fins, and dorsal surface of pectoral and pelvic fins covered by large yellowish-white spots. Ventral surface cream to yellowish white (Figs 14 and 15).

#### Color in life

Body ground color brown at head, trunk, and fins. Head covered by small yellowish-white dots; dorsum, flank, and dorsal, caudal, and anal fins, and dorsal surface of pectoral and pelvic fins covered by large yellowish-white spots, larger than pupil diameter. Ventral surface light brown (Fig 5E).

#### Distribution

*Hopliancistrus xavante* is currently known from small tributaries of the middle portion of Rio Xingu (Fresco and Jauri rivers) and upper Rio Xingu basin at Cachoeira von Martius and its tributaries such as Rio Culuene, Rio Curisevo, and Rio Sucuri (Fig 8).

#### Etymology

The new species is named after the Xavante, an indigenous ethnic group nowadays inhabiting several Indigenous Lands that form part of its earlier territory of traditional occupation, in the region comprised by the Serra do Roncador and the valleys of the Rio das Mortes, Rio Culuene, Rio Couto de Magalhães, Rio Batovi, and Rio Garças, in the eastern Mato Grosso State.

#### Remarks

Two lots included in the type series of *Hopliancistrus tricornis* by Isbrücker & Nijssen [4] (IRSNB 692 with 20 specimens and ZMA 120.339 with 19), from the von Martius Waterfall of the Xingu River basin, are instead the species herein described.

### Key to the species of *Hopliancistrus* Isbrücker & Nijssen, 1989

1. Four branched rays on the anal-fin…………………………*H*. *tricornis* (Tapajós basin)

1’. Five branched rays on the anal-fin…………………………………………………2

2. Head, trunk, and fins covered by conspicuous yellowish-white small dots of similar size and uniformly spaced.……………………………*H*. *wolverine* (middle Xingu basin)

2’. Head and trunk covered by yellowish-white somewhat faint spots of varying sizes along the body, smaller in the snout and head and larger towards caudal fin……………3

3. Nuchal plate covered by thick skin, lacking exposed odontodes (Fig 7F); caudal peduncle depth 11.5–12.9% of SL; nasal bone plate broad, length approximately equal width, sometimes slightly triangular (Fig 2B)…………………*H*. *xavante* (upper Rio Xingu)

3’. Nuchal plate exposed and ornamented by odontodes (Fig 7A–7E); caudal peduncle depth 10.0–11.3% of SL; nasal bone plate narrow, length approximately two times width (Fig 2A)………………………………………………………4

4. Preserved specimens with overall dark coloration on body and fins; fins uniformly darkened with inconspicuous black spots……………………………………….*H*. *xikrin* (tributaries of middle Xingu basin, not on main channel)

4'. Preserved specimens with overall brown coloration with irregular and inconspicuous pale blotches; fins with conspicuous black spots on rays………………….*H*. *munduruku* (upper Rio Jamanxim and Rio Curuá)

### Osteology of *Hopliancistrus*

#### Skull

**Fig 16:**
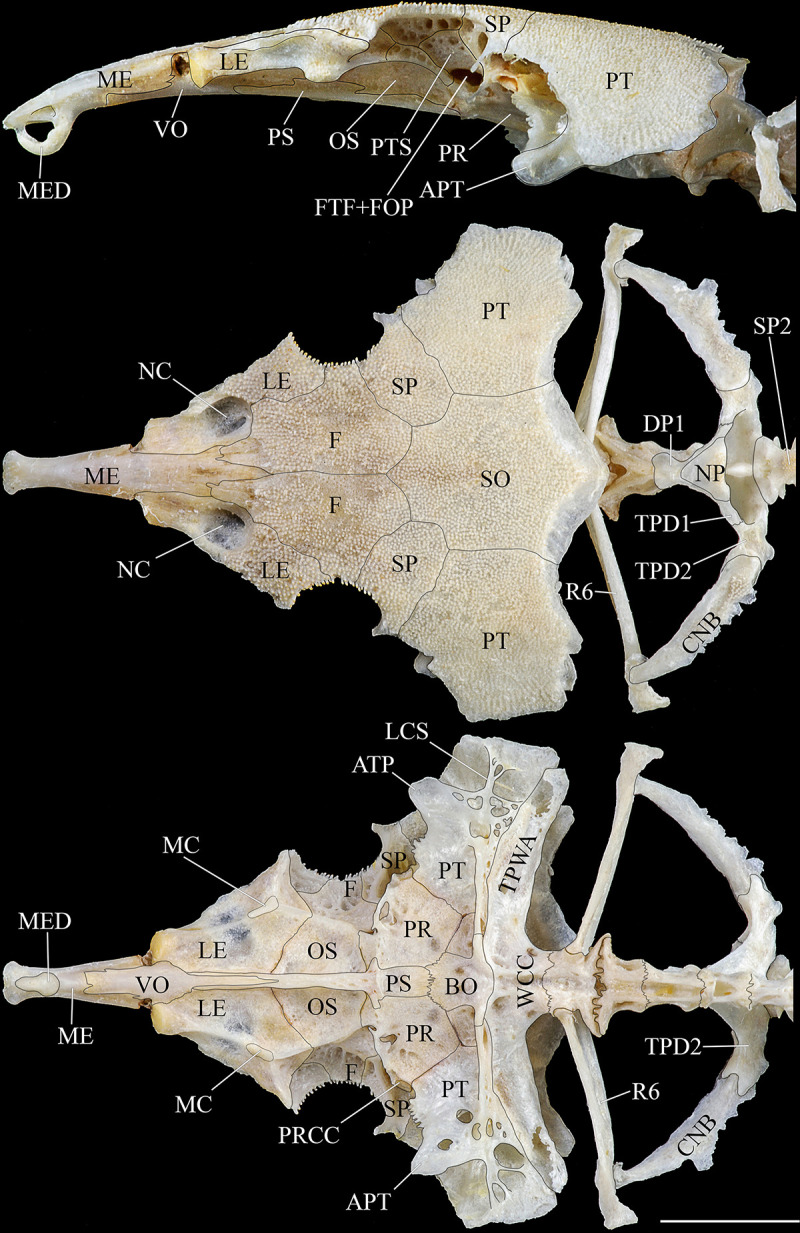
Skull bones of *H*. *wolverine*, INPA 40875, 136.1 mm SL, in lateral, dorsal and ventral views. APT- anterior-ventral process of compound pterotic, BO- basioccipital, CNB- connecting bone, DP1- first dorsal-fin pterygiophore, F- frontal, FTF+FOP- compound trigeminofacialis and optic nerve foramen, LCS- latero-posterior connection strut, LE- lateral ethmoid, MC- metapterygoid condyle of lateral ethmoid, ME- mesethmoid, MED- mesethmoid disk, NC- nasal capsule, NP- nuchal plate, OS- orbitosphenoid, PR- prootic, PRCC- cartilaginous condyle of prootic, PS- parasphenoid, PT- compound pterotic, PTS- pterosphenoid, R6- expanded rib of sixth vertebra, SO- Parieto-supraoccipital, SP- sphenotic, SP2- second dorsal-fin spine, TPD1- transverse process of first dorsal-fin pterygiophore, TPD2- transverse process of second dorsal-fin pterygiophore, TPWA- transverse process of the Weberian apparatus, VO- vomer, WCC- weberian complex centrum. Scale bar 10 mm.

Depressed; cranial roof-bones delicate and completely covered by odontodes (Fig 2). Dorsal profile of cranial-roof bones convex in lateral view; skull triangular, anteriorly narrow and posteriorly expanded in dorsal view; larger width at pterotic-supracleithrum (Fig 16).

Mesethmoid short and slender, cylindrical in cross section. Dorsal surface smooth and covered by small plates. Middle portion wider than rest of bone, narrowing anteriorly. Anterior tip of mesethmoid flattened and expanded laterally, narrower than middle portion; ventrally, mesethmoid disk well-developed and perforated. *Hopliancistrus tricornis*, *H*. *munduruku*, *H*. *xavante* and *H*. *xikrin* with anterior border of mesethmoid rounded, *H*. *wolverine* with small notch medially. Mesethmoid with dorsal expansion sutured to frontals posteriorly and laterally to lateral ethmoid (Fig 16); ventral surface with large rectangular notch sutured to vomer (Figs 6 and 16).

Lateral ethmoid well-developed and strongly ossified, triangle-shaped in ventral view (similar condition in *Peckoltia*, see Armbruster [25]), and posteriorly contacting frontal, and orbitosphenoid ventrally; anteriorly connected to vomer. Lateral ethmoid posterolateral processes well-developed and forming anterior margin of orbit, and contacting ventral margin of dermal plate between frontal and sixth infraorbital. Lateral ethmoid narrow anteriorly, with well-developed articular facet with palatine on ventral area. Condyle articulating with posterodorsal margin of metapterygoid, ovoid, large and positioned on posterior concavity of lateral ethmoid. Ridge on lateral ethmoid thin, extending from anterior point of contact with metapterygoid to ovoid condyle; in *Hopliancistrus tricornis*, *H*. *wolverine* and *H*. *xikrin* this ridge moderately deep, whereas in *H*. *munduruku* and *H*. *xavante* distinctly deep. Nasal capsule enclosed in lateral ethmoid near ridge (Figs 6 and 16).

Vomer elongated and relatively narrow, slightly longer than mesethmoid, and expanded medially. Anterior half sutured to mesethmoid; posterior half sutured laterally to lateral ethmoid, and posteriorly to parasphenoid and posteriorly reaching vertical through anterior margin of orbitosphenoid (Figs 6 and 16).

Parasphenoid elongated and narrow, anterior portion between contralateral lateral ethmoid, at midline sutured to orbitosphenoid and posteriorly to prootics. Parasphenoid posteriorly expanded and sutured to basioccipital; pair of small lateral wings sutured to anterior internal facet of prootic. Anteriorly narrow and deeply divided by vomer, fork-shaped, posterior to wings deeper than orbitosphenoid and lateral ethmoid (Figs 6 and 16).

Orbitosphenoid ranging from trapezoidal to slightly square-shaped, of similar size of prootic, and with ventral profile convex. Posteriorly connected to prootic, anteriorly to lateral ethmoid, medial margin to parasphenoid and lateral margin free (Figs 6 and 16). Sutured to frontal dorsally, forming internal facet of orbit, along with pterosphenoid and lateral ethmoid.

Prootic large, forming floor of neurocranium. Ventral facet plane with two large foramina at anterolateral margin. Dorsally prolonged sutured to compound pterotic and to sphenotic and pterosphenoid forming posterior wall of trigeminofacial and optic foramina (FTF+FOP [13]). Lateroposteriorly sutured to basioccipital and posteriorly with synchrondral articulation to exoccipital and compound pterotic. Anteriorly connected to orbitosphenoid, medial facet connected to parasphenoid, and lateral margin with cartilaginous condyle that articulates hyomandibula (Figs 6 and 16).

Basioccipital, small and strongly sutured to parasphenoid and small portion of prootic anteriorly, and laterally connected to exoccipital. Posterior portion of bone connected to Weberian complex, and with pair of thin lateral wings connected to transcapular process (Figs 6 and 16). Base of skull with large groove to accommodate facial and cranial musculature. Exoccipital slightly triangular and small; ventral facet plane with posterior border extended ventrally and connected to anterior facet of transcapular process; medial facet connected to basioccipital. Exoccipital anterolaterally shares synchrondral joint with prootic and compound pterotic.

Pterosphenoid large, rectangular; ventral surface contributing to dorsal wall of trigeminofacial and optic foramina along with prootic. Anteriorly connected to orbitosphenoid, dorsally to frontal and posteriorly to sphenotic, forming internal wall of orbit (Fig 16).

Frontals large, slightly triangular in dorsal view, expanded medially forming dorsal border of orbit (Figs 2 and 16). Anteriorly, frontals narrow and connected to nasal plate, and forming small portion of border of nasal capsule. Frontals medially sutured together (cranial fontanel absent); posteriorly connected to sphenotic and parieto-supraoccipital. Nasal elongated in *Hopliancistrus tricornis*, *H*. *munduruku*, *H*. *wolverine* and *H*. *xikrin*, and broad in *H*. *xavante*. Nasal capsule completely encased ventrally by lateral ethmoid.

Sphenotic roughly rectangular with rounded edges in dorsal view. Ventral process thin and elongated, ventrally surpassing half height of orbit, and forming posteromedial margin of orbit, with external contact with eighth infraorbital (Figs 2 and 6).

Parieto-supraoccipital large (only smaller than compound pterotic), flat, octogonal in shape and forming posterior portion of skull dorsally covered by odontodes on dorsal surface. Posterior process broad, rounded and not raised, posteriorly limited by four large plates (Figs 2 and 16).

Compound pterotic rectangular and greatly expanded laterally, with small perforations uniformly distributed on dorsolateral surface. Anteroventral process of compound pterotic separated medially from main body of bone (Figs 2, 6 and 16).

Infraorbital canal running through eight platelets, eighth and seventh platelets forming posteroventral border of orbit (Fig 2).

#### Splanchnocranium

**Fig 17:**
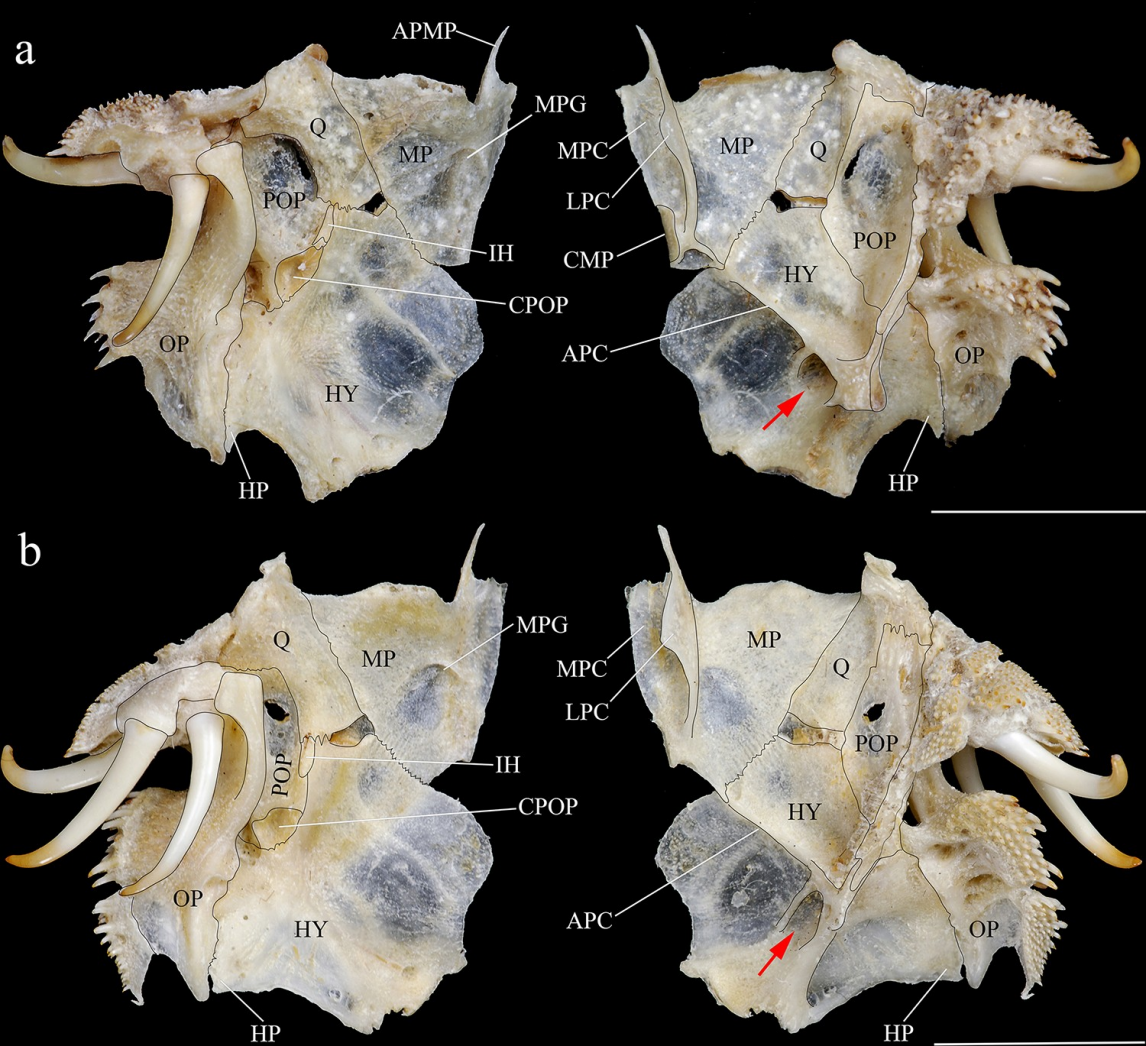
Suspensorium in ventral and dorsal views, right side. (a) *Hopliancistrus tricornis*, INPA 7025, 114.3 mm SL; (b) *H*. *wolverine*, INPA 40875, 136.1 mm SL. Arrows point the fovea on base of levator arcus palatini crest. APC- levator arcus palatini crest, APMP- anterior process of metapterygoid, CMP- condyle of metapterygoid, CPOP- condyle of preopercle, HP- posterior process of hyomandibula, HY- hyomandibula, IH- interhyal, LPC- lateral wall of the pterygoid channel, MP- metapterygoid, MPC- mesial wall of the pterygoid channel, MPG- metapterygoid groove, OP- opercle, POP- preopercle, Q- quadrate. Scale bar 10 mm.

Metapterygoid large, triangular, positioned on anterolateral portion of suspensorium, sutured to quadrate anteroventrally and hyomandibula posteriorly. Metaptertygoid anterior border sinuose, gently deflected ventrally. Metapterygoid channel large, deep, long-walled, and with thin anterior process straight, and elongated (with tip slightly expanded). Ventral wall of metapterygoid channel more developed than dorsal, and with edentate suture to lateral ethmoid anteriorly, with condyle posteriorly. Dorsal wall elongated, with border triangular, resting on large facet of lateral ethmoid. Ventral surface of metapterygoid with groove more developed in *Hopliancistrus wolverine* and *H*. *xikrin* (Fig 17).

Hyomandibula large (largest bone in suspensorium), synchondrally sutured to metapterygoid anteriorly. Hyomandibula separated from quadrate by symplectic cartilage; anterolateral facet jointed to preopercle. Hyomandibula has two posterior connections to skull: facet with cartilaginous condyle connected to prootic and digitized suture to compound pterotic. Posteromedial portion of hymandibula forming ventral wall of orbit. Dorsal surface with well-developed *levator arcus palatini* crest, with profound conical fossa on *levator arcus palatini* crest base (Fig 17). Posterior region of hyomandibula greatly deflected ventrally. Portion of hyomandibula posterior to opercle developed into shelf, with thin and pointed process extending posteriorly from opercular condyle, to which opercle has secondary attachment. Opercular condyle of hyomandibula well-developed.

Quadrate narrow, elongated and anteriorly expanded (L-shaped in dorsal view); dorsal surface flat, longitudinal ridge absent. Quadrate sutured to metapterygoid and laterally to preopercle, bearing small edentate suture to hyomandibula posteriorly. Quadrate anterior tip composed by large condyle articulating to autopalatine (Fig 17). Interhyal tiny, slender and connected to anterolateral facet of hyomandibula (Fig 6C); anterior margin contacting cartilage on anterior tip of hyomandibula and quadrate (character 26, state 2 [25]). Interhyal united to posterior ceratohyal via ligament.

Preopercle roughly rectangular, sutured to quadrate anteriorly and hyomandibula posteriorly. Preopercle-hyomandibular ridge high, continuous and covered by small platelets. Symplectic foramen cartilage present on anterior portion of preopercle. Ventral facet of preopercle with small condyle articulating with posterior ceratohyal (Fig 17).

Opercle with pointed anteriorly directed process, anterior to opercular condyle, which fits within depression on lateral face of preopercle. Posteriorly, opercle connected to preopercle by extensive synchondral joint (Fig 17).

Autopalatine short and robust, anterior tip slightly curved laterally and enlarged (Fig 6). Anterior tip of autopalatine connected to maxilla by large block of cartilage. Posteriorly with well developed rounded condyle connected to lateral ethmoid. Autopalatine posterior condyle with three processes, two lateral and one medial, latter two serving as insertion site for extensor muscles [13]. Maxilla relatively broad and flattened, with size similar to that of autopalatine. Maxilla articulated to autopalatine via two condyles.

**Fig 18:**
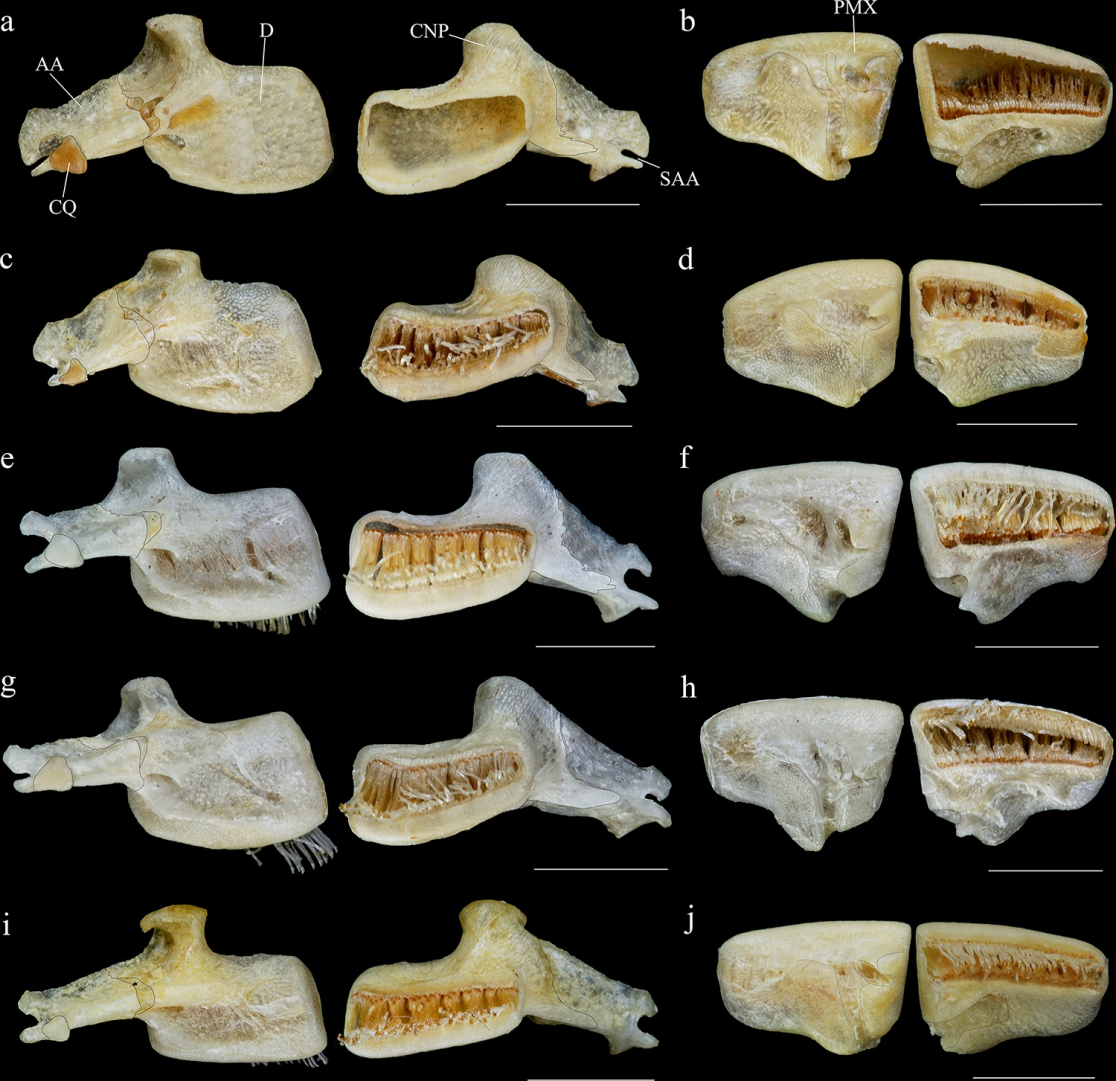
Dentary and premaxillary in dorsal and ventral views, left side. (a, b) *H*. *tricornis*, INPA 7025, 114.3 mm SL; (c, d) *H*. *munduruku*, INPA 38255, 73.3 mm SL; (e, f) *H*. *wolverine*, INPA 43731, 122.2 mm SL; (g, h) *H*. *xikrin*, INPA 40904, 133.8 mm SL; (i, j) *H*. *xavante*, INPA 37616, 114.7 mm SL. AA- angulo articular, CQ- condyle of articulation of quadrate, CNP- coronoid process, D- dentary, PMX- premaxillary, SAA- slit of angulo articular. Scale bar 5 mm.

Premaxilla roughly rectangular; dorsal surface with well developed groove and condyle for articulation with palatine cartilage. Dorsal ridge well-developed to insertion of adductor muscle. Premaxillary bones joint mesially, and articulated to mesethmoid disk through cartilage block. Ventral facet with rectangular opening, housing teeth. Teeth numerous and small, bicuspid with medial cusp diminute. Dentary somewhat rectangular, with well-developed rounded coronoid process and concave posteriorly (dentary deeper in *H*. *tricornis*). Dentary with bony laminar expansion strongly sutured to anguloarticular posteriorly, and connected via thick cartilage anteriorly. Anguloarticular L-shaped in cross section. Anterior area of anguloarticular with slit above condyle of articulation with quadrate. In *H*. *tricornis* slit very narrow, and dorsal facet wider than anguloarticular condyle. In *H*. *munduruku* slit more developed than in *H*. *tricornis*, but condyle smaller than in other species, and dorsal facet much wider than anguloarticular condyle. In *H*. *wolverine* and *H*. *xavante* slit wider and deeper than in *H*. *tricornis*, *H*. *munduruku* and *H*. *xikrin*, with dorsal facet narrower than condyle. In *H*. *xikrin* slit narrow but with dorsal facet narrower than condyle (Fig 18).

**Fig 19:**
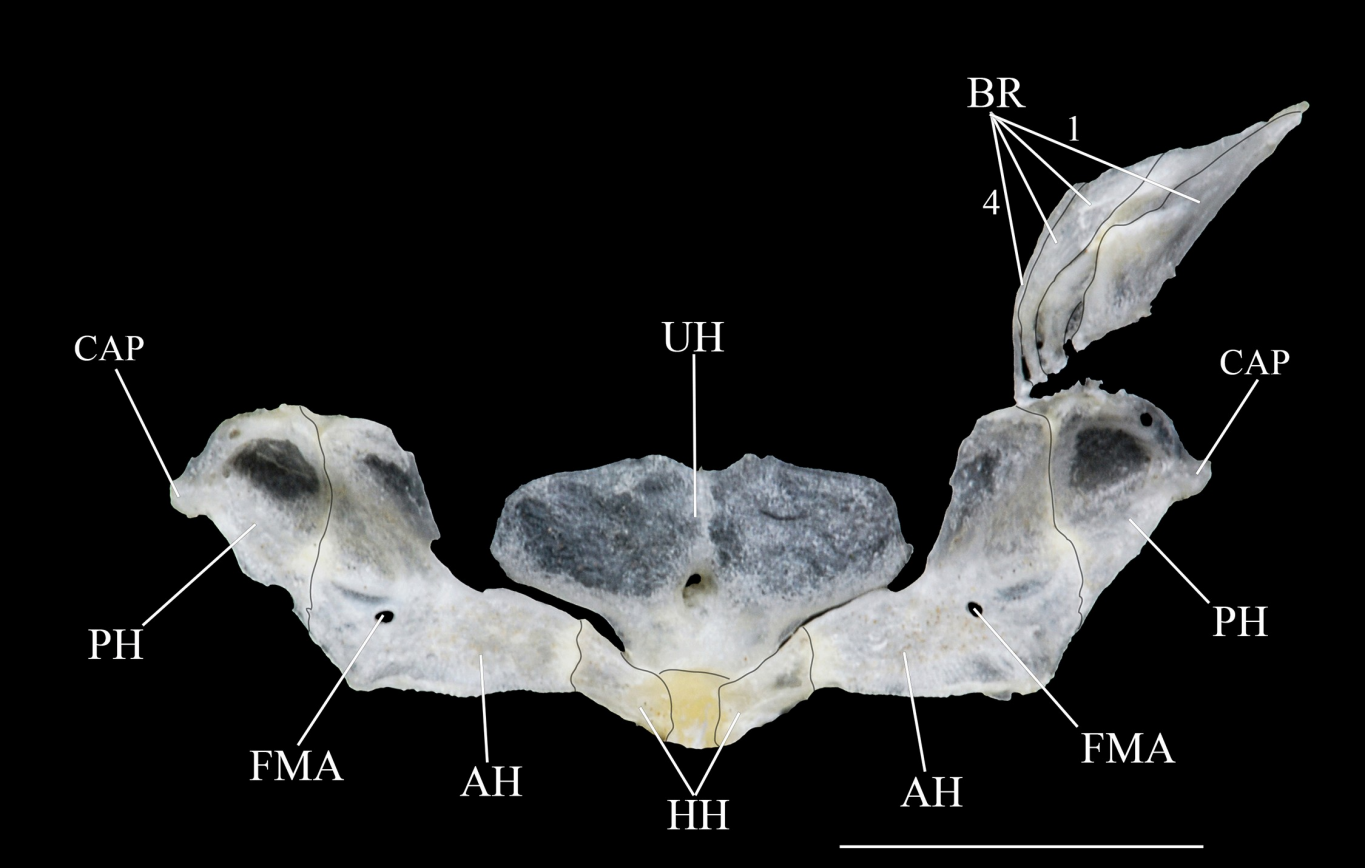
Hyoid arch ventral view of *H*. *wolverine* INPA 43731, 122.2 mm SL. AH- anterohyal, BR- branchiostegal, CAP- condyle of articulation of preopercle, FMA- foramen for afferent mandibular artery, HH- hypohyal, PH- posterohyal, UH- urohyal. Scale bar 10 mm.

**Fig 20:**
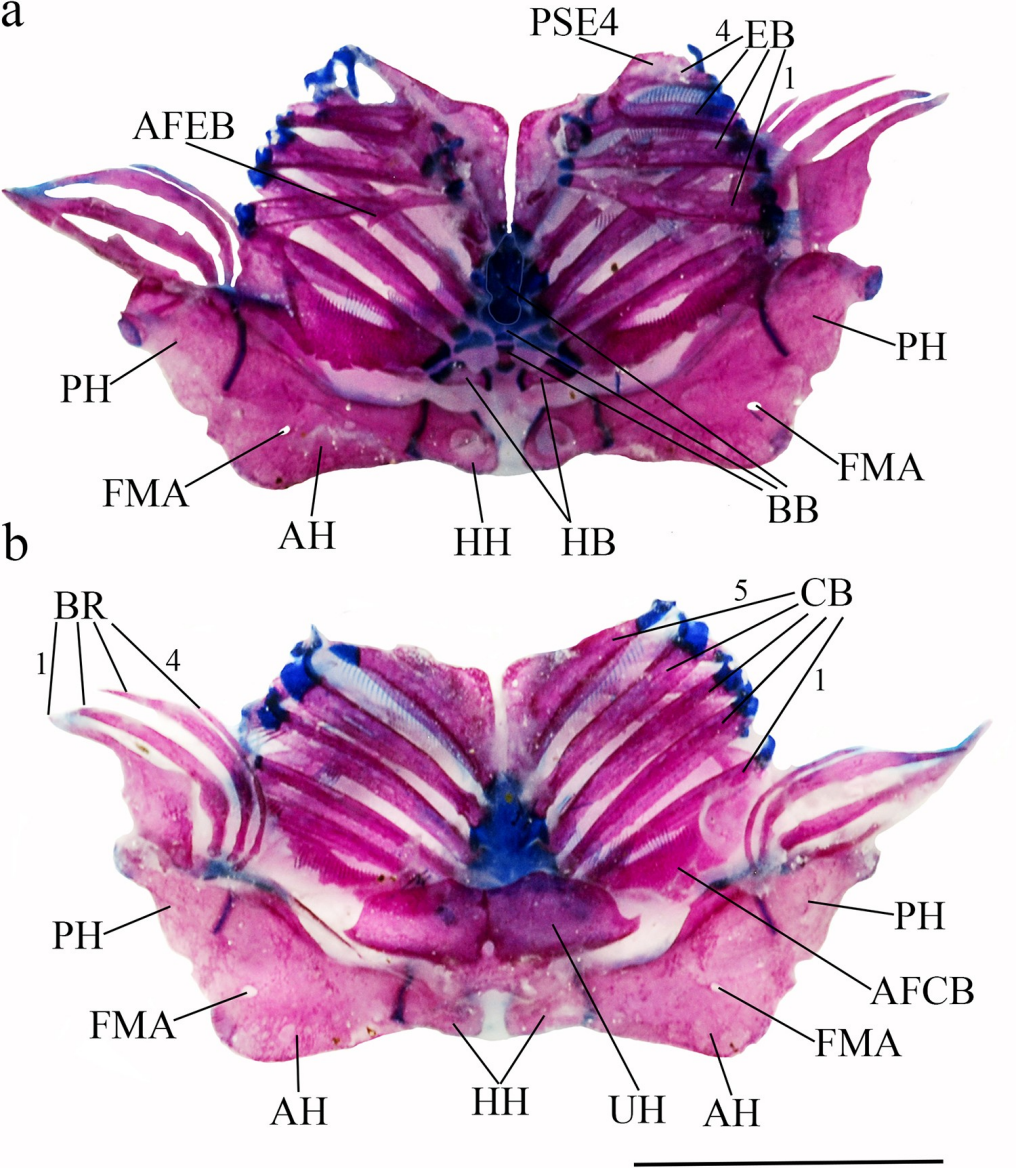
**Hyoid and branchial arches of *H*. *tricornis*, INPA 58207, 93.8 mm SL, in dorsal (a) and ventral (b) views.** AFCB- accessory flange of first ceratobranchial, AFEB- accessory flange of first epibranchial, AH- anterohyal, BB- basibranchial, BR- branchiostegal, CB- ceratobranchial, EB- epibranchial, FMA- foramen for afferent mandibular artery, HB- hypobranchial, HH- hypohyal, PH- posterohyal, PSE4- posterior shelf of fourth epibranchial. Scale bar 10 mm.

Posterohyal roughly triangular, with lateral border largest, and L-shaped in cross section. Lateral wall of the posterohyal pouch laterally curved externally extending lateral border of posterohyal (Fig 19). Condyle for articulation with preopercle small and positioned on ventrolateral margin. Posterohyal synchondrally connected to anterohyal except small suture on anterior portion (Fig 20). Four branchiostegal rays; first distinctly wider, and with small condyle of contact with ceratohyal. Last branchiostegal contacting adjacent cartilage between posterohyal to anterohyal (Fig 20). Anterohyal wide and flat dorsoventrally, with anterior margin (in dorsal and ventral views) straight from medial tip to approximately half bone, then distinctly convex towards suture with posterohyal. Anterohyal bearing small rounded foramen for afferent mandibular artery on posterior half of bone. Anterohyal connected to hypohyal by cartilage. Dorsal and ventral hypohyal not distinguishable, probably fused. Hypohyal short and rectangular, connected to counterpart, as well as to dentary via thick cartilage. Dorsal surface with large fossa for insertion of anterior process of urohyal. Urohyal well-developed, wider than longer, bearing strong anterior processes that articulate to hypohyals.

First hypobranchial ossified with cartilaginous caps, elongate and slender medially, with lateral portion slightly expanded. Second, third, fourth and fifth hypobranchials cartilaginous and indistinguishable from cartilaginous cap of ceratobranchials. First basibranchial ossified with cartilaginous caps and with half length of first hypobranchial. Second and third basibranchial cartilaginous; second being tiny and third robust and elongate, occupying space between bases of third to fifth ceratobranchials. Fifth ossified ceratobranchials, rod-like and with cartilaginous caps. First ceratobranchial with large accessory flange, as long as ceratobranchial. Fifth ceratobranchial with middle portion expanded, triangular-shaped, bearing lower pharyngeal tooth plates. Lower pharyngeal plate with vestigial teeth on its mesial border. Four ossified epibranchials, rod-like and with cartilaginous caps. First epibranchial bearing small ventrally directed process. Fourth with short anteriorly directed process and with well developed posterior shelf (Fig 20). Two infrapharyngobranchials, first connected to third epibranchial short and slender, expanded posteriorly; second square and connected to anterior facet of fourth epibranchial and upper pharyngeal tooth plates. Upper pharyngeal tooth plates slightly triangular; tooth plates with mesial shelf with raised bulbous area (comma-shaped in ventral view), bearing small teeth restricted to bulbous area and posterior edge of shelf.

**Fig 21:**
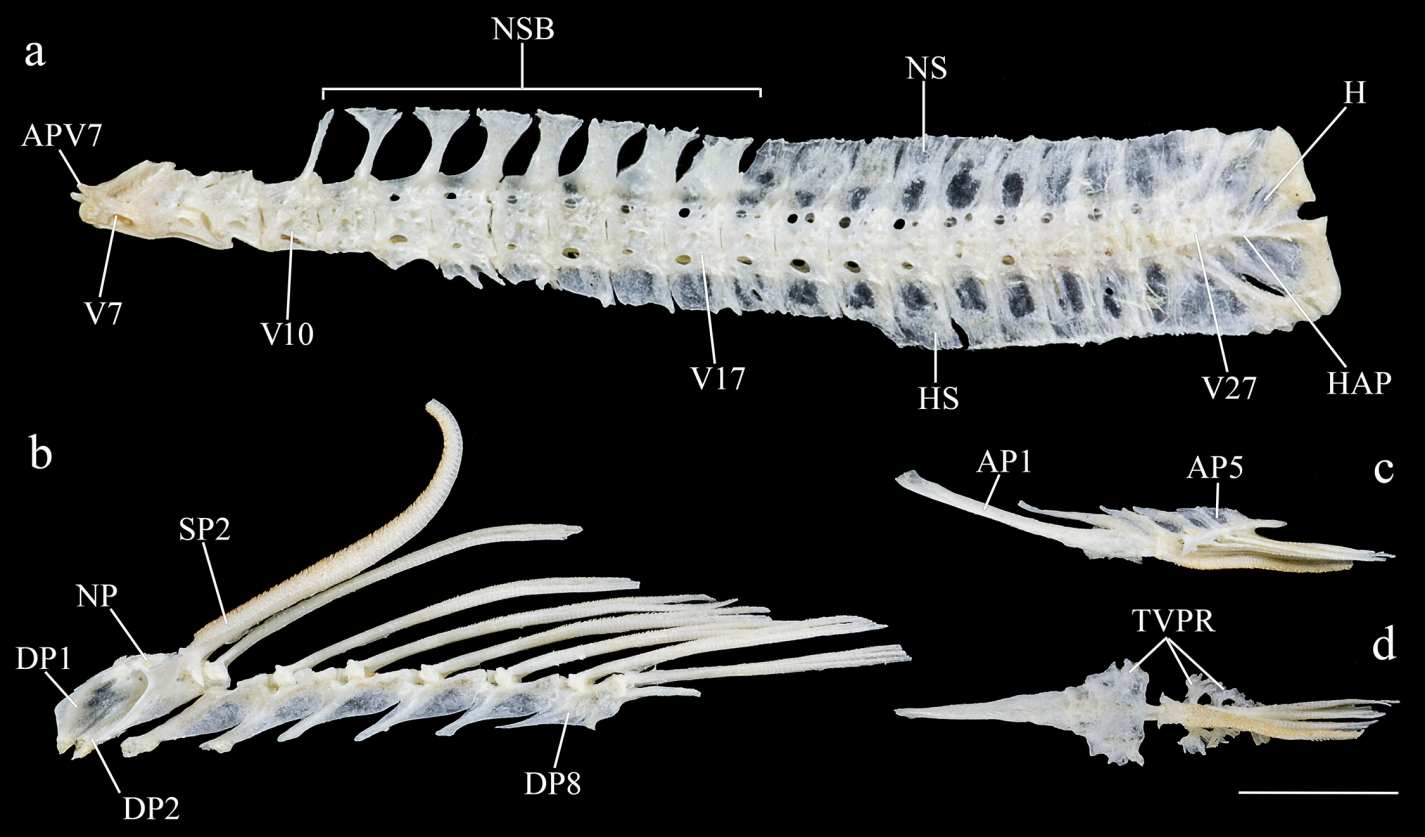
**Lateral views left side of vertebral column (a), dorsal-fin (b), and lateral (c) and ventral (d) views of anal-fin of *H*. *wolverine*, INPA 40875, 136.1 mm SL.** AP1- first anal-fin pterygiophore, AP5- fifth anal-fin pterygiophore, APV7- anterior process of seventh centrum, DP1- first dorsal-fin pterygiophore, DP2- second dorsal-fin pterygiophore, DP8- eighth dorsal-fin pterygiophore, H- hypural plate, HAP- hypurapophysis, HS- hemal spine, NP- nuchal plate, NS- neural spine, NSB- neural spine bifid, V7-V27- seventh to twenty seventh vertebral centrum, SP2- second dorsal-fin spine, TVPR- transverse process. Scale bar 10 mm.

Weberian complex centrum (WCC) relatively short and composed of vertebral centrum 1 to 5. Swimbladder encapsulated on ventral expansion of transverse process of WCC. Transverse process narrowing distally with rounded tip contacting compound pterotic (Fig 6). Ventral process of WCC roughly circular, short, not reaching the sixth vertebrae. First completely free, sixth vertebra with rectangular neural arch sutured to parieto-supraoccipital; sixth and seventh vertebrae expanded laterally in area they connect (Fig 6). Ribs of sixth vertebra (first rib) large and split medially, articulating to both centrum and neural arch; rib enlarged (Fig 6). Seventh centrum with pointed anteriorly directed process on dorsal portion, not reaching the tuberculum of rib of sixth centrum (Fig 21A). Ribs on vertebrae six, eventually on eighth, and always present from centra 9 to 16. Vertebra 8 bearing ribs only in some specimens of *H*. *xavante* and *H*. *xikrin*. Vertebral centra 8, 9 and sometimes 10 lacking neural spines; seven to eight bifid neural spines; first bifid neural spine on tenth, or sometimes on eleventh centrum. *Hopliancistrus tricornis*, *H*. *munduruku*, *H*. *xavante* and *H*. *xikrin* with 11 vertebrae after last neural bifid spine, whereas *H*. *wolverine* with 10. Vertebrae with 16 to 17 haemal spines; first haemal spine on eleventh or twelfth centrum. Haemal spines of 14, 15, 16, 17 and 18th vertebrae with small bifurcation on posterior portion to support anal-fin pterygiophores (Fig 21A).

Caudal skeleton with parhypural, hypural 1, and 2 fused forming a ventral plate, uroneural and epural fused to hypurals 3, 4 and 5 forming a dorsal plat; ventral plate larger than dorsal plate; ventral and dorsal plates fused at base with notch on posterior border (notch smaller in *H*. *tricornis*); hypurapophysis triangular and laterally directed (Fig 21A).

Nuchal plate extremely reduced in *H*. *tricornis*, large in *H*. *munduruku*, *H*. *wolverine* and *H*. *xikrin*, and covered with skin in *H*. *xavante*, although odontodes can be seen on skin (Figs 2 and 7). First and second dorsal-fin pterygiophores fused and forming nuchal plate, and with condyle on dorsal surface to support spinelet. First and second dorsal-fin pterygiophore connected along entire posterior and anterior margins, respectively, with small edentate suture on dorsal portion (Fig 21B). First pterygiophore loosely connected to short neural spine of seventh centrum; second pterygiophore loosely connected to neural spines of seventh and eighth centra. Second pterygiophore largest and with transverse process (TPD2) distally connected to connecting bone (CNB) (Fig 16). Connecting bone extended to rib of sixth vertebra. Transverse processes present on first five (of eight) pterygiophores; eighth supporting sixth and seventh branched rays, and with par of elongate Y-shaped posterior processes (Fig 21B). Dorsal-fin spinelet very reduced and V-shaped; short ventral arms of spinelet slide under transverse process of nuchal plate, but locking mechanism not functional. Dorsal-fin spine unbranched, robust.

Adipose-fin composed of single pre-adipose plate, spine, and posterior membrane. In *Hopliancistrus tricornis*, *H*. *munduruku*, and *H*. *xikrin* pre-adipose plate positioned over neural spine of vertebrae 22, and adipose-fin spine over 23; in *H*. *wolverine* and *H*. *xavante* pre-adipose plate over neural spine 23 and adipose-fin spine over 24.

**Fig 22:**
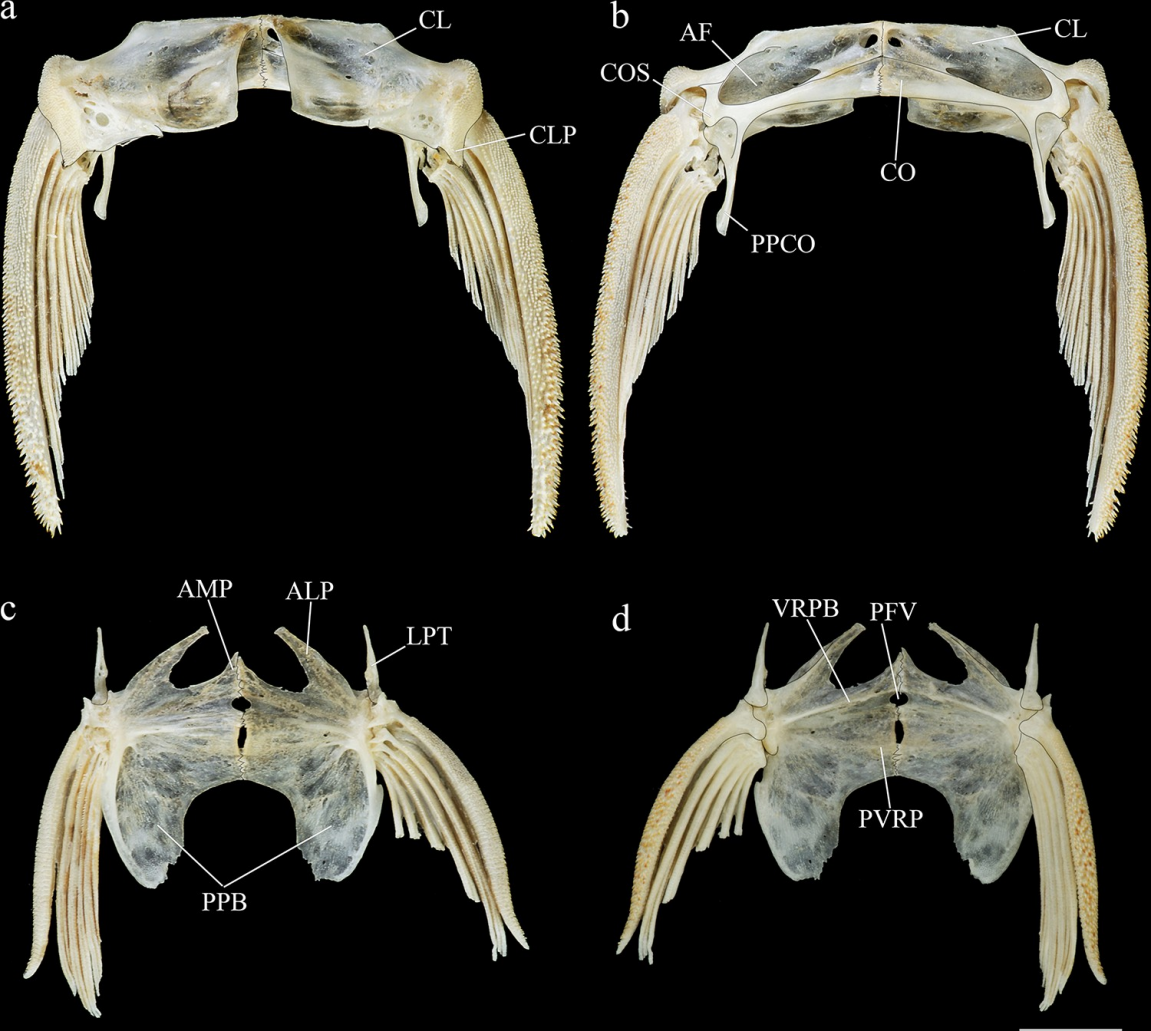
Pectoral and pelvic girdles in dorsal and ventral views of *H*. *xikrin* INPA 40904, 133.8 mm SL. AF- abductor fossa, ALP- anterolateral process of pelvic basipterygium, AMP- anteromesial process of pelvic basipterygium, CL- cleithrum, CLP- cleithrum posterior process, CO- coracoid, COS- lateral strut of coracoid, LPT- lateropterygium, PFV- fenestra of pelvic basipterygium, PPB- posterior process of pelvic basipterygium, PPCO- fenestra of pelvic basipterygium, VRPB- ventral ridge of pelvic basipterygium, PVRP- posteroventral ridge of pelvic basipterygium. Scale bar 10 mm.

Pectoral girdle strong, broad; cleithral process short, covered by odontodes on lateral facet. Anterior margin of cleithrum (in dorsal and ventral views) straight medially, and concave laterally. Posterodorsal process of cleithrum deep, and articulating to notch of compound pterotic. Ventral surface of pectoral girdle with large fossa for abductor muscles and base of pectoral-fin spine. Posterior process of coracoid relatively elongated, longer than last pectoral-fin ray (Fig 22A and 22B). Ventral surface or pectoral girdle covered by skin.

Basipterygium composed of anterolateral, anteromedial, and posterior processes. Anterolateral process of basipterygium not reaching counterpart at midline; anteromedial process with wide base, and sutured to counterpart. Lateropterygium short (smaller than anterolateral process). Anterior fenestra of basipterygium rounded and small. Basipterygium sutured to counterpart, except for a small portion in which it is connected via cartilage (Fig 22C and 22D).

Anal fin with 4 (*Hopliancistrus tricornis*) or 5 pterygiophores; fist pterygiophore elongated and posteriorly expanded laterally, not exposed, contacting posterior portion of haemal spine of 14th vertebral centrum. In *H*. *munduruku* and *H*. *xikrin* first pterygiophore longer than in congeners, and anterior tip almost reaching haemal spine of 13th vertebral centrum. Unbranched ray of anal-fin supported by first pterygiophore; first and second branched rays supported by second pterygiophore, last pterygiophores supporting only one ray (Fig 21C and 21D).

## Discussion

Species of *Hopliancistrus* of the Rio Tapajós basin are currently found in its middle portion only, even though several expeditions were conducted to its main upstream tributaries, the Juruena and Teles Pires rivers. On the other hand, *Hopliancistrus* are widespread in stretches with rapids and rocky substrate of the Rio Xingu basin. As far as we know, no other genus of Ancistrini has its distribution restricted to these two basins; the closest situation is that of *Parancistrus*, which is known only from the Xingu and Araguaia-Tocantins basins. Generally, Ancistrini genera that include species adapted to rapids present wider distributions that include the Araguaia-Tocantins basin and/or rivers draining the Guyana Shield at the northern margin of the Amazonas basin in Pará state, such as *Baryancistrus*, *Hypancistrus*, *Panaqolus*, *Panaque*, *Peckoltia*, *Pseudacanthicus*, *Pseudancistrus*, *Scobinancistrus* and *Spectracanthicus*.

The current distribution of *Hopliancistrus* indicates that the most recent ancestral species of the genus emerged after the tectonic events that shaped the Amazon basin, such as the reversal of the direction of the Amazonas River [26,27]. We would expect that the species radiated from an ancient connection between the two basins, possibly where now lies the headwaters of the Jamanxim and Curuá rivers, which are currently separated by a dry stretch of approximately 23 km in straight line.

*Hopliancistrus* belongs to Ancistrini by having fully eversible cheek plates with enlarged odontodes (also present in Pterygoplichthini); and by having the opercle modified into a sickle-shaped structure (a unique feature of Ancistrini). *Hopliancistrus* is unique by having three claw-like hypertrophied curved odontodes on the cheek plates, and short stiff odontodes on the lateral border of the snout, two exclusive features among loricariid catfishes. Enlarged to hypertrophied odontodes on the lateral border of the snout are present in several other cis-andean loricariids (e.g., *Delturus*, *Pareiorhaphis*, *Lasiancistrus*, *Pseudancistrus*) as well as trans-andean (*Dolichancistrus*). Whereas odontodes are elongate, thin, and somewhat flexible in the former species, these are short and stiff in *Hopliancistrus*. Nevertheless, large specimens of *Dolichancistrus* and *Chaetostoma* have few very long odontodes, somewhat resembling the condition found in *Hopliancistrus*. However, the condition in *Hopliancistrus* is quite modified. Besides carrying these strong and few odontodes, *Hopliancistrus* use them as a protective weapon, everting them deliberately to hold and stab the aggressor.

The osteological study of the species of *Hopliancistrus* revealed some interesting characters that could help us understand the phylogenetic position of the genus. *Hopliancistrus* has a conical and deep fossa on the base of the *levator arcus palatini* muscle crest, positioned laterally to preopercle-hyomandibular crest, with its opening turned to the dorsal portion of hyomandibula. A similar condition of the fossa was also observed in *Chaetostoma jegui* Rapp Py-Daniel, 1991, *C*. *dorsale* Eigenmann, 1922, *C*. *thomsoni* Regan, 1904, *Dolichancistrus fuesslii* (Steindachner, 1911), *Guyanancistrus brevispinis* (Heitmans, Nijssen & Isbrücker, 1983), *G*. *niger* (Norman, 1926) and *Panaqolus nix* Cramer & Rapp Py-Daniel, 2015. However, because only a few of the species of *Chaetostoma*, *Dolichancistrus* and *Guyanancistrus* were examined in the present study, it is impossible to confirm whether the presence of a conical fossa constitutes a synapomorphic condition for all these taxa. On the other hand, we have also examined other *Panaqolus* species, *P*. *claustellifer* Tan, Souza & Armbruster, 2016, *P*. *purusiensis* (La Monte, 1935) and *P*. *tankei* Cramer & Sousa, 2016, and these three lack the fossa.

**Fig 23:**
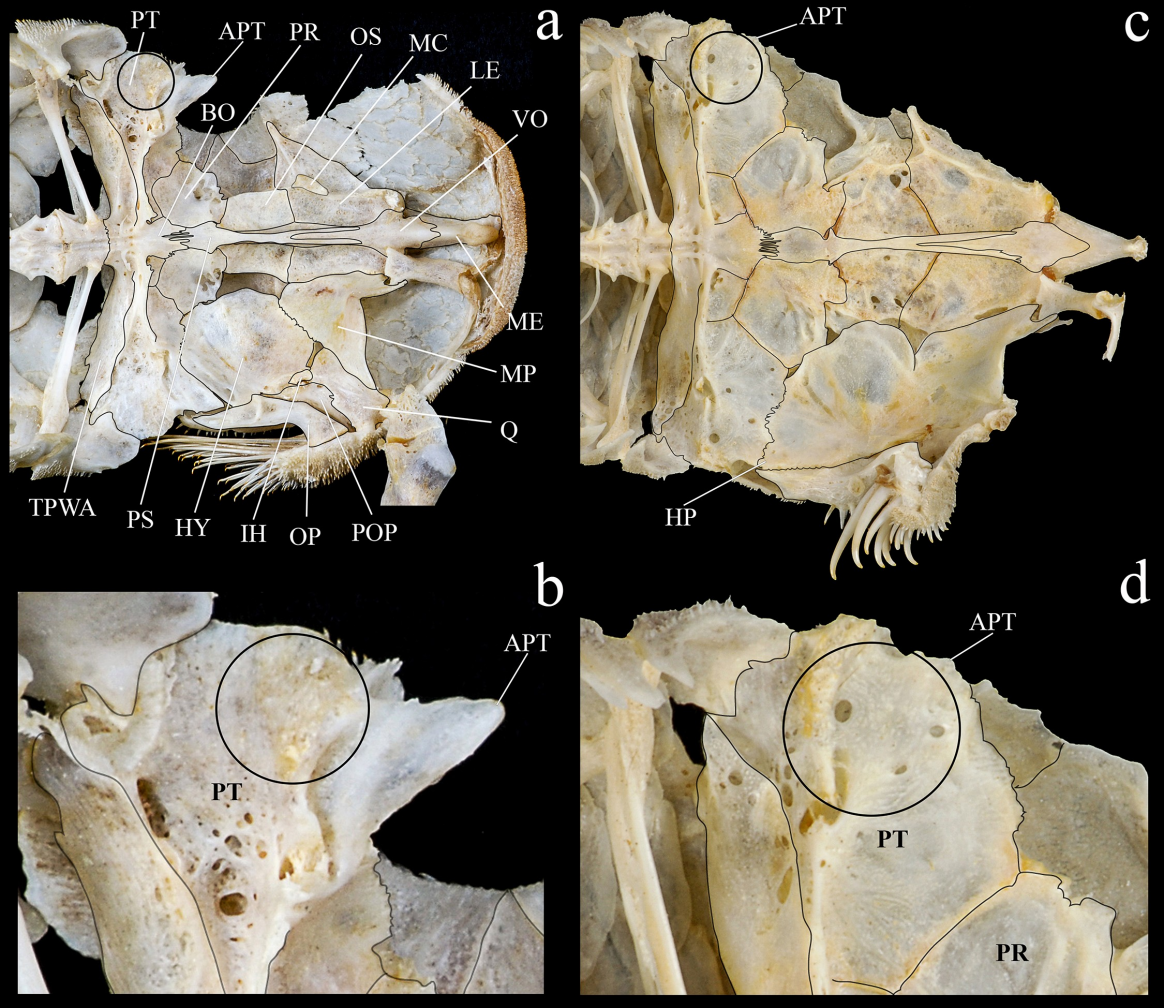
Ventral views and enlarged, right side, of anterior portion of body. (a, b) *Baryancistrus chrysolomus*, INPA 43458, 213.4 mm SL; (c, d) *Ancistrus ranunculus*, INPA 37616, 119.9 mm SL, showing the antero-ventral process of compound pterotic (APT), and latero-posterior bone shelf connection with the principal body of compound pterotic (black circles). BO- basioccipital, HP- posterior process of hyomandibula, HY- hyomandibula, IH- interhyal, LE- lateral ethmoid, MC- metapterygoid condyle of lateral ethmoid, ME- mesethmoid, MP- metapterygoid, OP- opercle, OS- orbitosphenoid, POP- preopercle, PR- prootic, PS- parasphenoid, PT- compound pterotic, Q- quadrate, TPWA- transverse process of the Weberian apparatus, VO- vomer.

In most loricariids, the compound pterotic has an anteroventral process located laterally to the main axis of the bone (Fig 23). In *Hopliancistrus*, the antero-ventral process of the compound pterotic is always present and connected dorsally to the top of the compound pterotic. The compound pterotic in *Hopliancistrus* is quite peculiar in comparison to other loricariids, as the bone is robust and forms almost a box, with a large chamber between the top and floor of the bone. Ventrally, the compound pterotic has the ossified Baudelot ligament extended laterally as a branched bony strut providing different openings for the internal weberian capsule (Fig 6). This huge chamber inside the compound pterotic is completely connected to the other side through a set of foramina on the internal dorsal wall of the weberian complex.

The presence of the branched bony strut on the main body of the compound pterotic, as well as the fossa on the base of the *levator arcus palatini* muscle crest, might be responsible for the insertion of muscles that support and move the opercle and the three hypertrophied odontodes of the cheek plate, such as the *dilatator operculi* muscle.

**Fig 24:**
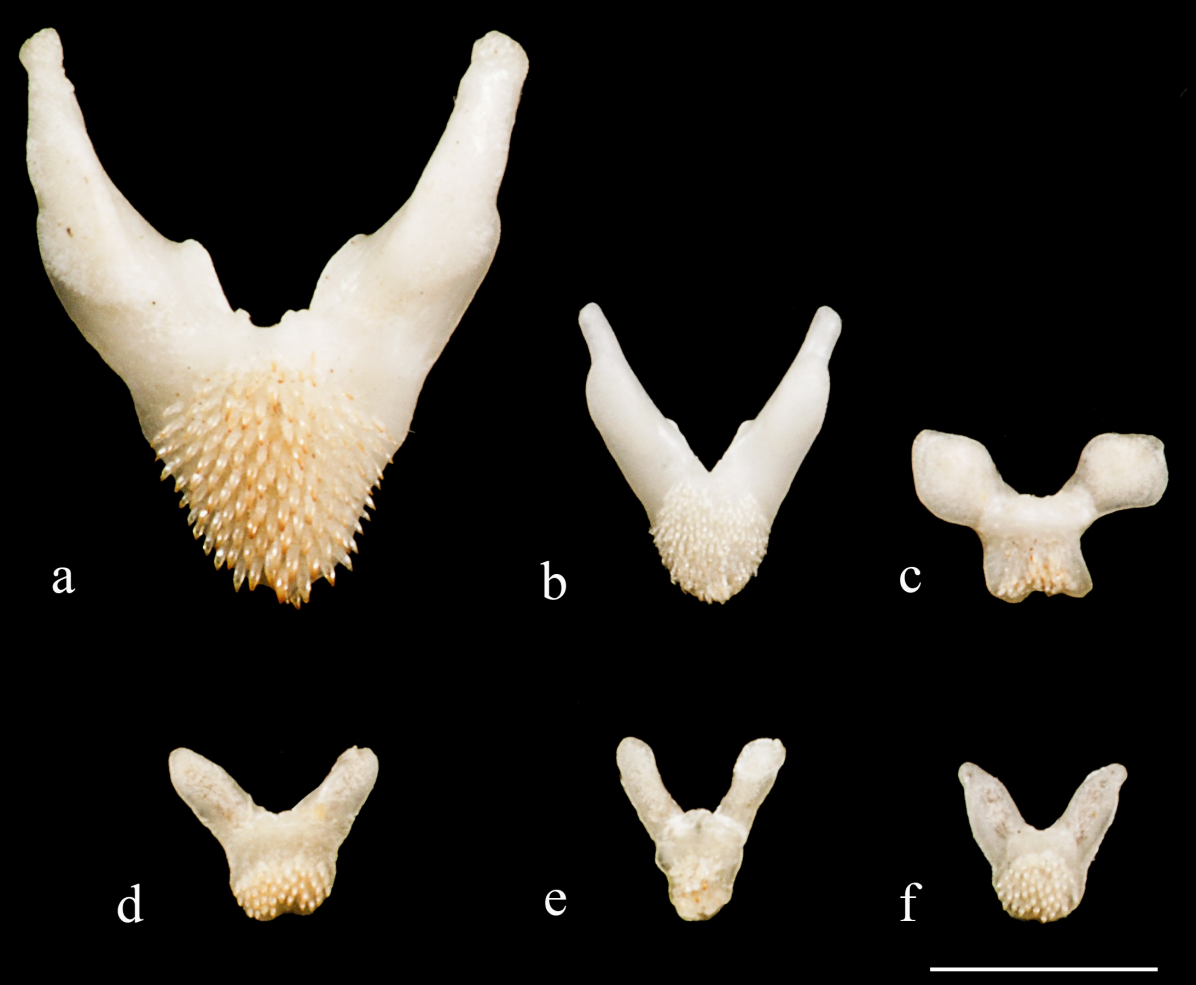
First spine of dorsal-fin or spinelet. (a) *Baryancistrus chrysolomus*, INPA 43458, 213.4 mm CP, (b) *Peckoltia vittata* INPA 43452, 103.0 mm CP, (c) *Ancistrus ranunculus* INPA 43456, 119.9 mm CP, (d) *Hopliancistrus wolverine*, INPA 40875, 136.1 mm SL, (e) *H*. *xavante*, INPA 37616, 114.7 mm CP, and (f) *H*. *xikrin*, INPA 40904, 131.6 mm CP. Scale bar 5 mm.

The spinelet in Ancistrini, in general, is well-developed, presenting two ventral elongate arms. *Hopliancistrus* has the smallest spinelet among all studied genera, with a non-functional locking mechanism. This condition is also present in *Ancistrus*, *Araichthys*, *Dekeyseri*a and *Exastilithoxus hoedemani*, although with spinelet slightly more developed than in *Hopliancistrus* (Fig 24).

Schaefer [13] found a great variation in opercle shape among loricariids. Ancistrini was diagnosed by Schaefer [13] based on modifications of the opercle. The Ancistrini present two shapes: 1. the opercle is relatively straight with posterior lateral section reduced, *Peckoltia*-type (sickle-shaped, following [25]), present in the genera that have bristle-like odontodes on cheek plates (e.g. *Baryancistrus*, *Peckoltia*, *Panaque* and *Scobinancistrus*) (Fig 23A); 2. the opercle is strongly tridimensional, slender and twisted anteriorly, with posterior lateral section expanded, *Ancistrus*-type, (bar-shaped [25]), present in the genera that have strongly hypertrophied odontodes on cheek plates (e.g. *Ancistrus*, *Chaetostoma*, *Dolichancistrus*, *Dekeyseria* and *Hopliancistrus*) (Fig 23C). Especially in *Hopliancistrus*, the opercle bone is strong, heavy, and very curved laterally in its anterior portion, with posterior lateral section very expanded, exposed, and covered by odontodes (Fig 17). *Hopliancistrus* also differs from most of the Ancistrini genera, except from *Ancistrus*, *Araichthys*, *Exastilithoxus*, *Chaetostoma*, *Dekeyseria*, *Dolichancistrus*, *Lasiancistrus*, *Lithoxus* and *Pseudolithoxus*, by the possession of a laminar expansion of the hyomandibula immediately posterior to the opercular condyle (Figs 6 and 17).

According to Lundberg [28], the presence of five infraorbitals is the plesiomorphic condition within catfishes; however, the majority of loricariids has five or six infraorbital bones [13,25, RRO, pers. obs.]. Thus, the presence of up to eight infraorbital platelets in *Hopliancistrus* is a derived condition among others loricariids. Seven to eight infraorbitals was also observed in *Ancistomus*, *Aphanotorulus*, *Baryancistrus*, *Hypancistrus*, *Leporacanthicus*, *Panaqolus*, *Panaque*, *Peckoltia*, *Peckoltichthys*, *Pseudacanthicus* and *Scobinancistrus*.

The distal portion of the posterohyal in Ancistrini is generally pointed and conical with the lateral wall forming a pouch [25]. In *Hopliancistrus*, the distal portion of the posterohyal is nearly flat, and the pouch absent. This condition is also present in *Ancistrus*, *Araichthys*, *Chaetostoma dorsale*, *Dekeyseria*, *Dolichancistrus*, *Exastilithoxus*, *Lasiancistrus*, *Lithoxus*, *Peckoltichthys* and *Pseudolithoxus* (Fig 19).

The anteroventral process of the compound pterotic in most Ancistrini is separated medially from its main body and connected only by a strut, leaving a gap between the main body of the compound pterotic and the strut [25]. However, the size of the gap is influenced by the width of the strut. In *H*. *tricornis* and *H*. *wolverine*, the strut is very narrow and bar-shaped, leaving a large gap between the connecting strut and the main body of the compound pterotic. In *H*. *munduruku*, *H*. *xavante* and *H*. *xikrin*, the strut is wide or continuous, forming a wall mesially between the compound pterotic and the anteroventral process, sometimes leaving a small gap posteriorly (Fig 6).

Armbruster [29] considered *Hopliancistrus* closely related to a clade comprising *Lithoxus*, *Exastilithoxus*, and the *Chaetostoma* group, which included *Chaetostoma*, *Cordylancistrus*, *Dolichancistrus*, and *Leptoancistrus*. This large clade is related to the *Ancistrus* group, which includes *Ancistrus*, *Lasiancistrus*, and *Pseudolithoxus*. Later on, Lujan et al. [30], based on molecular data, achieved results substantially divergent from previous hypotheses based on morphology. Ancistrini (sensu Armbruster [25,29]) was not recovered as a monophyletic group, and thus subdivided into seven clades. Ancistrini was restricted to the genera *Ancistrus*, *Lasiancistrus*, *Pseudolithoxus*, *Soromonichthys*, *Guyanancistrus*, *Dekeyseria*, *Neblinichthys*, *Paulasquama*, *Lithoxancistrus*, and *Hopliancistrus*. In the hypothesis of Lujan et al. [30], *Hopliancistrus* was recovered as sister group to *Corymbophanes kaiei*, forming a clade with *Guyanancistrus* and *Dekeyseria*.

A morphology-based cladistic analysis including *Hopliancistrus* is under preparation by the first author (RRO) and depicts a well-supported clade with *Pseudolithoxus* as sister group to two smalls clades, one composed by (*Araichthys* + *Hopliancistrus*) and other by (*Lasiancistrus* + *Ancistrus*). These results are similar to Roxo et al. [31], with a single difference that *Guyanancistrus brevispinis* was recovered as sister group to *Araichthys* + *Hopliancistrus*. The relationships of theses large clades are weakly supported in Armbruster’s [24,29] analyses, possibly because of the limited number of species used. As knowledge and understanding of morphological variation in loricariids improve we expect more congruent results on morphological and molecular approaches.

## Comparative examined material

*Acanthicus hystrix*: INPA 40452, 3 alc, 112.4–188.3 mm SL and 1 cs, 130.9 mm SL, Brazil, Pará, Anapu, Rio Xingu. *Ancistomus feldbergae*: INPA 28953, 7 alc, 29.6–131.1 mm SL and 2 cs 58.0–71.3 mm SL, paratypes, Brazil, Pará, Altamira, Rio Xingu. *A*. *sabaji*: ANSP 179211, 3, 34.3–95.8 mm SL, paratypes, Guyana, Rupununi, Rupununi River. INPA 43851, 1 skel, 163.8 mm SL, Brazil, Pará, Altamira, Rio Xingu. *A*. *snethlageae*: INPA 43774, 1 alc, 140.2 mm SL and 1 skel, 131.0 mm SL, Brazil, Pará, Itaituba, Rio Tapajós. *A*. *spilomma*: INPA 25714, 2 alc, 127.0–127.3 mm SL and 1 cs 124.4 mm SL, Brazil, Pará, Canaã dos Carajás, Rio Parauapebas. *Ancistrus dolichopterus*: INPA 38779, 7 alc, 13.8–88.3 mm SL and 1, cs 85.6 mm SL, Brazil, Amazonas, Santa Isabel do Rio Negro, Rio Negro. *A*. *hoplogenys*: INPA 53228, 5 alc, 26.0–141.9 mm SL and 1 cs, 107.4 mm SL, Brazil, Pará, Castanhal, Rio Apeú. *A*. *karajas*: INPA 37584, 7 alc, 23.4–42.4 mm SL and 3 cs, 26.2–36.2 mm SL, paratypes, Brazil, Pará, Parauapebas, Rio Parauapebas. *A*. *krenakarore*: INPA 37593, 57 alc, 19.0–77.0 mm SL and 6 cs, 19.0–46.5 mm SL, paratypes, Brazil, Pará, Rurópolis, Rio Itapacurá. *A*. *maximus*: INPA 37614, 21 alc, 59.1–199.9 mm SL and 2 cs, 82.9–123.0 mm SL, paratypes, Brazil, Roraima, Rorainópolis, Macoari stream, Rio Branco basin. *A*. *ranunculus*: INPA 43456, 3 alc, 97.2–118.0 mm SL and 2 skel, 111.9–119.9 mm CP, Brazil, Pará, Altamira, Rio Xingu. *Araichthys loro*: NUP 12261, 1 cs, 49.7 mm SL and NUP 12262, 1 cs, 47.3 mm SL, Brazil, Mato Grosso, Sapezal, Rio Juruena. *Baryancistrus chrysolomus*: INPA 43458, 4 alc, 37.7–179.3 mm SL and 2 skel, 208.7–213.4 mm SL, Brazil, Pará, Altamira, Rio Xingu. *B*. *xanthellus*: INPA 43385, 5 alc, 38.2–168.0 mm SL and 2 skel, 209.5–225.4 mm SL, Brazil, Pará, Altamira, Rio Xingu. *Chaetostoma dorsale*: MPUJ 7300, 2 alc, 72.5–73.1 mm SL and 1 cs, 65.5 mm SL, Colômbia, Tane, cuenca del Río Casanare. *C*. *jegui*: INPA 33840, 3 alc, 73.9–102.9 mm SL and 1 cs, 49.7 mm SL, Brazil, Roraima, Caracaraí, Rio Uraricoera. *C*. *thomsoni*: MPUJ 7864, 3 alc, 63.8–78.2 mm SL and 1 cs, 76.5 mm SL, Colômbia, Villeta, cuenca del Río Villeta. *Dekeyseria amazonica*: INPA 52823, 5 skel, 110.9–160.3 mm SL, Brazil, Amazonas, Iranduba, Lago Catalão. *D*. *picta*: INPA 53025, 22 alc, 60.5–135.6 mm SL and 1 skel, 119,4 mm SL, Brazil, Amazonas, Novo Airão, Rio Negro. *D*. *scaphirhyncha*: INPA 414, 8 alc, 71.0–179.7 mm SL and 1 cs 140.1 mm SL, Brazil, Amazonas. *Dolichancistrus fuesslii*: MPUJ 7250, 2 alc, 57.2–69.6 mm SL and 1 cs, 63.3 mm SL, Colômbia, Chámeza, cuenca del Río Cusiana, Meta. *Exastilithoxus* cf. *fimbriatus*: INPA 49712, 1 cs, 30.7 mm SL, Brazil, Amazonas, São Gabriel da Cachoeira, Rio Negro. *E*. *hoedemani*: INPA 38957, 2 alc, 29.9–47.5 mm SL and 1 cs, 37.3 mm SL, Brazil, Roraima, Amajari, Rio Auaris. *Guyanancistrus brevispinis*: INPA 3309, 11 alc, 67.2–101.9 mm SL and 1 cs 85.9 mm SL, Guiana Francesa, Sinnamary, Salto Mouche. *G*. *niger*: INPA 6262, 9 alc, 95.5–121.3 mm SL and 1 cs, 99.5 mm SL, Brazil, Pará, Almeirim, Rio Jari. *Hemiancistrus guahiborum*: INPA 51148, 33 alc, 73.4–128.3 mm SL and 2 cs, 67.9–81.8 mm SL, Brazil, Amazonas, Santa Isabel do Rio Negro, Rio Marauiá. *H*. *subviridis*: INPA 51149, 5 alc, 95.8–117.7 mm SL and 1 cs, 105.6 mm SL, Brazil, Amazonas, Santa Isabel do Rio Negro, Rio Marauiá. *Hypancistrus inspector*: INPA 49602, 5 alc, 70.0–86.4 mm SL and 1 cs 91.3 mm SL, Brazil, Amazonas, São Gabriel da Cachoeira, Rio Negro. *H*. *zebra*: INPA 26587, 3 alc, 29.0–44.9 mm SL and 1 cs 46.0 mm SL, Brazil, Pará, Altamira, Rio Xingu. *Hypancistrus* sp. “pão”: INPA 40520, 10 alc, 33.0–102.5 mm SL and 1 cs 90.8 mm SL, Brazil, Pará, Altamira, Rio Xingu. *Lasiancistrus schomburgkii*: INPA 28802, 13 alc., 26.6–58.2 mm SL and 1 cs, 80.0 mm SL, Brazil, Mato Grosso, Aripuanã, Rio Aripuanã. *L*. *tentaculatus*: MPUJ 9370, 1 alc, 63.1 mm SL and 1 cs, 55.7 mm SL. *Leporacanthicus galaxias*: INPA 6359, 272 alc, 28.0–129.7 mm SL and 1 cs, 106.3 mm SL and 2 skel, 119.4–142.6 mm SL, Brazil, Pará, Tucuruí, Rio Tocantins. *L*. *heterodon*: INPA 43386, 2 alc, 42.8–53.5 mm SL and 1 skel, 142.6 mm SL, Brazil, Pará, Altamira, Rio Xingu. *L*. *joselimai*: INPA 25856, 16 alc, 46.4–68.7 mm SL and 3 cs, 50.0–85.6 mm SL. *Lithoxus lithoides*: INPA 845, 49 alc, 20.9–46.3 mm SL and 1 cs, 46.7 mm SL, Brazil, Amazonas, Presidente Figueiredo, Rio Uatumã. *Megalancistrus parananus*: NUP 4801, 1 cs 106.6 mm SL, Brazil, Paraná, Foz do Iguaçu. *Oligancistrus javae*: INPA 48004, 6 alc, 45.9–59.5mm SL and 1 cs, 74.8 mm SL, Brazil, Tocantins, Araguatins, Rio Araguaia. *O*. *punctatissimus*: INPA 43217, 5 alc, 51.0–74.6 mm SL and 1 skel, 124,1 mm CP, Brazil, Pará, Porto de Moz, Rio Xingu. *O*. *tocantinensis*: INPA 2990, 138 alc, 16.2–92.9 mm SL and 2 cs, 83.4–86.9 mm SL, paratypes, Brazil, Pará, Tucuruí, Rio Tocantins. *O*. *zuanoni*: INPA 43853, 5 alc, 38.8–116.0 mm SL and 2 skel, 114,4–144,0 mm SL, Brazil, Pará, Altamira, Rio Xingu. *Panaqolus nix*: INPA 43900, 4 alc, 50.6–72.6 mm SL and 1 cs 81.0 mm SL, Brazil, Rôndonia, Guajará-Mirim. *P*. *purusiensis*: INPA 55450, 12 alc, 21.7–134.6 mm SL and 1 cs, 73.4 mm SL, Brazil, Amazonas, Atalaia do Norte, Rio Javari. *P*. *tankei*: INPA 43175, 39 alc, 24.0–83.4 mm SL and 1 cs 84.6 mm SL, Brazil, Pará, Senador José Porfírio, Rio Xingu. *Panaque Armbrusteri*: INPA 43848, 1 skel, 290.1 mm SL, Brasil, Pará, Altamira, Rio Xingu. *Paralithoxus planquettei*: INPA 4586, 4 alc, 35.9–40.4 mm SL and 2 cs, 40.2–44.4 mm SL, Guiana Francesa, Sinnamary. *P*. *stocki*: INPA 5537, 3 alc, 41.8–58.2 mm SL and 1 cs, 58.7 mm SL, Brazil, Pará, Oriximiná, Rio Trombetas, Cachoeira Porteira. *Parancistrus aurantiacus*: INPA 48007, 56 alc, 56.7–172.0 mm SL and 3 skel, 143.8–171.9 mm SL, Brazil, Pará, Piçarra, Rio Araguaia. *P*. *nudiventris*: INPA 31421, 2 alc, 140.3–161.7 mm SL and 1 skel, 149.8 mm SL, Brazil, Pará, Altamira, Rio Xingu. *Peckoltia brevis*: USNM 408221, 3, cs 53.8–91.6 mm SL, Bolivia, Beni, Ballivia province, Río Matos. *P*. *compta*: INPA 6782, 3 alc, 45.3–61.5 mm SL and 1 cs 45.3 mm SL, paratypes, Brazil, Pará, Itaituba, Rio Jamanxim. *P*. *vittata*: INPA 43852, 1 skel, 102.8 mm SL, Brazil, Pará, Altamira, Rio Xingu. *Peckoltichthys bachi*: ANSP 68651, 87.2 mm SL, holotype, Peru, Río Ucayali. INPA 28209, 1 cs 94.8 mm SL, Brazil, Amazonas, Pauiní. *Pseudacanthicus leopardus*: INPA 25865, 3 alc, 37.4–66.8 mm SL and 1 cs, 54.0 mm SL, Brazil, Amazonas, Barcelos, Rio Demení. *P*. *major*: INPA 6309, 10, 62.3–116.8 mm SL and 1 cs 98.9 mm SL, Brazil, Tocantins, Tucuruí, Rio Tocantins. *P*. *histrix*: INPA 31804, 10 alc, 71.0–109.9 mm SL and 1 cs, 71.0 mm SL, Brazil, Pará, Vitória do Xingu, Rio Xingu. *Pseudancistrus asurini*: INPA 43691, 1 alc, 108.8 mm SL and 1 skel, 128.6 mm SL, Brazil, Pará, Altamira, Rio Xingu. *P*. *genisetiger*: UFPB 4429, 2 alc, 68.0–130.0 mm SL and 2 cs, 67.3–75.0 mm SL, Brazil, Paraíba, Nazarezinho, Olho d'Água do Frade. *Pseudolithoxus anthrax*: ANSP 162175, 3, 50.7–112.7 mm SL, paratypes and ANSP 193381, 1 cs, 92.2 mm SL, Venezuela, Amazonas. *P*. *nicoi*: INPA 42945, 2 alc, 76.9–125.0 mm SL and 1 cs 109.5 mm SL, Brazil, Amazonas, São Gabriel da Cachoeira, Rio Negro. *P*. *kinja*: INPA 16270, 6 alc, 97.5–153.1 mm SL and 1 skel, 146.8 mm SL, paratypes, Brazil, Amazonas, Presidente Figueiredo, Rio Uatumã. *Scobinancistrus aureatus*: INPA 43693, 4 alc, 43.4–65.2 mm SL and 1 skel, 225.2 mm SL, Brazil, Pará, Altamira, Rio Xingu. *S*. *pariolispos*: INPA 43694, 3 alc, 33.2–44.4 mm SL and 1 skel, 174.4 mm SL, Brazil, Pará, Altamira, Rio Xingu. *Spectracanthicus murinus*: INPA 6984, 29 alc, 25.7–71.5 mm SL and 2 cs, 53.7–64.8 mm SL, Brazil, Pará, Itaituba, Rio Tapajós.

## Supporting information

S1 Table Material examined (Percents). (XLSX)
